# An unusual [4 + 2] fusion strategy to forge *meso-N*/*O*-heteroarene-fused (quinoidal) porphyrins with intense near-infrared Q-bands†
†Electronic supplementary information (ESI) available: Detailed information on experimental procedures, characterization data, computational calculations, crystallographic and spectroscopic data, and X-ray crystal structures (CIF). CCDC 1857684 (**Zn1a**), 1852362 (**3aa**), 1852363 (**4ah**), 1865973 (*syn*-**5aa**), 1852372 (*syn*-**5af**), 1857685 (*syn*-**5ah**) and 1852366 (*anti*-**5ah**). For ESI and crystallographic data in CIF or other electronic format see DOI: 10.1039/c9sc01596e


**DOI:** 10.1039/c9sc01596e

**Published:** 2019-06-17

**Authors:** Chengming Li, Lei Zhu, Wenbo Liang, Rongchuan Su, Jiangliang Yin, Yanmei Hu, Yu Lan, Di Wu, Jingsong You

**Affiliations:** a Key Laboratory of Green Chemistry and Technology of Ministry of Education , College of Chemistry , Sichuan University , 29 Wangjiang Road , Chengdu 610064 , P. R. China . Email: jsyou@scu.edu.cn; b School of Chemistry and Chemical Engineering , Chongqing Key Laboratory of Theoretical and Computational Chemistry , Chongqing University , Chongqing 400030 , P. R. China

## Abstract

Here we present a divergent synthesis of brand-new types of *meso-N*/*O*-heteroarene-fused (quinoidal) porphyrins through Rh-catalyzed β-C–H activation/annulation of 5,15-dioxoporphyrins and dioxime derivatives with alkynes, in which the synthetic disconnections are difficult to access through the commonly used intramolecular cyclization strategy. Using the *O*-methyl oxime as a traceless oxidizing directing group, the *meso-N*-embedded pyridine-fused *anti*-quinoidal porphyrin **3** and pyridinium-fused cation **4** are formed with controllable chemoselectivity and complete *anti*-selectivity. Replacing the exocyclic oxime with a carbonyl group delivers the pyran-fused porphyrin **5**, achieving structural conversion from a quinoidal conformation to a stable porphyrin macrocycle. Further oxidation of the expanded dimer **5ea** gives the oxonium **6**, which exhibits intense near-infrared (NIR) Q-bands up to 1300 nm. Theoretical studies demonstrate that the incorporation of a heteroatom at the *meso*-position enables more effective π-extension, resulting in a 22π aromatic (*vs.* 18π aromatic) character of pyran-fused porphyrins (*syn*/*anti*-**5aa**). Compared with the commercially available methylene blue (**MB**), *syn*-**5al** exhibits a better ability (*Φ*_Δ_ = 0.61) to sensitize singlet oxygen (^1^O_2_) when irradiated with a 680 nm laser beam, and has potential as a photodynamic therapy (PDT) photosensitizer in the body's therapeutic window (650–900 nm).

## Abstract 2

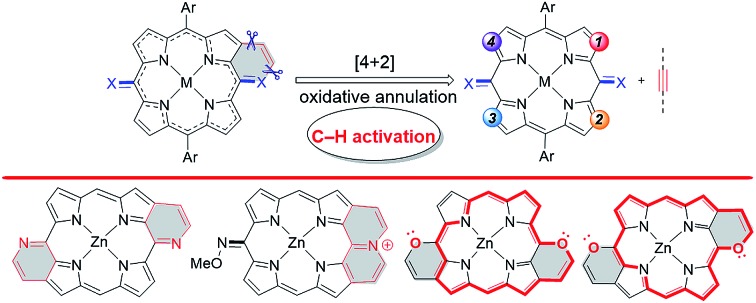
Divergent synthesis of *meso-N*/*O*-heteroarene-fused (quinoidal) porphyrins was established *via* rhodium-catalyzed β-C–H activation/annulation of quinoidal porphyrins with alkynes.

## Introduction

Much attention has been paid to structurally modified π-conjugated porphyrins with unique optical and electronic properties.^1^–^4^ Fusing an aromatic segment directly to the porphyrin peripheral framework is undoubtedly one of the most efficient methods to expand conjugated electronic systems,^5^–^8^ among which *meso*-heteroatom-containing heteroarene-fused porphyrins are an attractive research subject. The coplanarization of the lone pair of peripheral *meso*-heteroatoms with the π-electronic system of the porphyrin macrocycle enables more efficient π-extension.^6^,^7^ The commonly used synthetic strategies rely on the coupling reactions of *meso*/β-halide with arylamine, lithium diphenylphosphide, pyridine-2-thiol or 2-trimethylsilylphenyl zinc chloride, followed by intramolecular cyclization. Despite significant advances, these strategies generally involve the tedious multistep synthesis of porphyrin precursors, leading to a relatively low synthetic efficiency and limited structural diversity of fused porphyrins. Thus, developing straightforward access to these types of heteroarene-fused porphyrins is an appealing task in porphyrin chemistry.

**Scheme 1:**
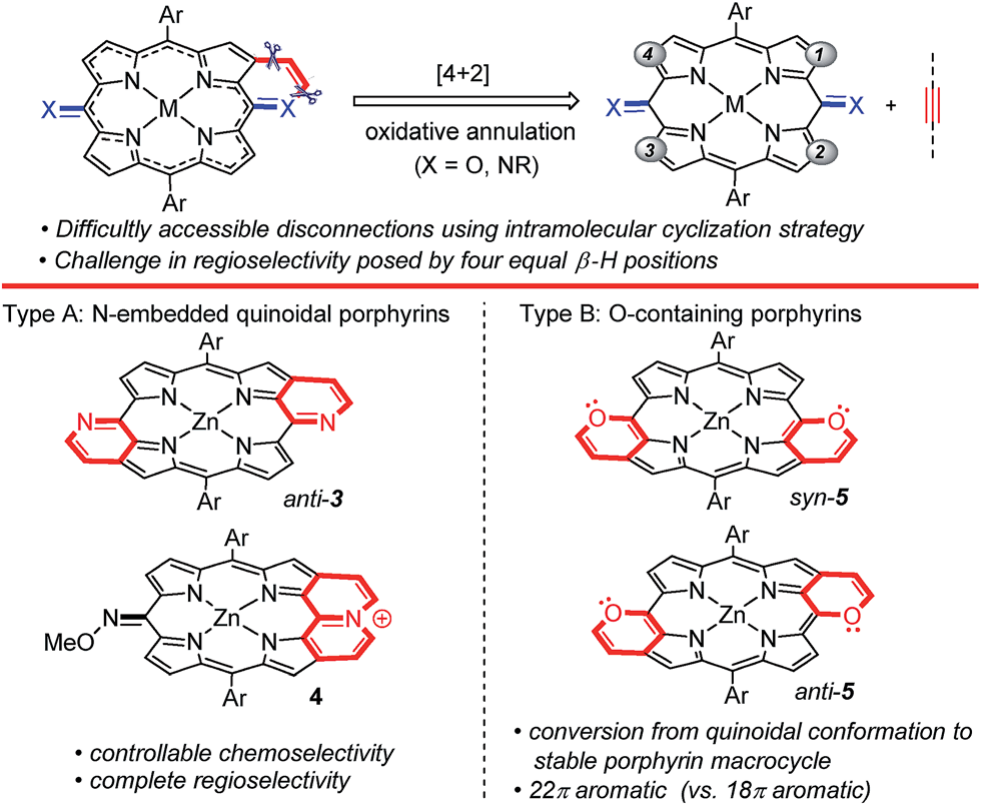
β-C–H activation/annulation of 5,15-dioxoporphyrins and their dioxime derivatives with alkynes for the synthesis of heteroarene-fused (quinoidal) porphyrins.

Transition metal-catalyzed annulations with alkynes through chelation-assisted C–H activation have been proved to be one of the most efficient accesses to π-conjugated heterocycles over the last decade.^9^,^10^ However, peripheral modification to construct heteroarene-fused porphyrins through this strategy remains undeveloped. Considering that chelating heteroatoms such as nitrogen and oxygen are able to serve as both the directing group and the merging functionality,^11^ we envisaged that *meso*-heteroatom-containing six-membered heteroarene-fused (quinoidal) porphyrins could be rapidly constructed by direct [4 + 2] oxidative annulation of 5,15-dioxoporphyrins and their dioxime derivatives with alkynes through β-H cleavage, in which the synthetic disconnections are difficult to access without a C–H activation/annulation strategy (Scheme 1).^12^

The regioselective control of the above [4 + 2] oxidative annulation strategy could be a challenging task owing to the four equal β-H positions. In this work, using the internally oxidative *ortho*-C–H activation strategy, we have established a rhodium-catalyzed oxidative [4 + 2] annulation of readily available *O*-methyl dioximes of 5,15-dioxoporphyrins with alkynes, affording the doubly pyridine-fused *anti*-quinoidal porphyrins **3** and pyridinium-fused cations **4** with controllable chemoselectivity and complete regioselectivity (Scheme 1, type A). When 5,15-dioxoporphyrins are employed as the substrate, the doubly pyran-fused porphyrins **5** are obtained rather than quinoidal products (Scheme 1, type B).

## Results and discussion

### Synthesis and X-ray crystallographic analysis of pyridine/pyridinium-fused quinoidal porphyrins

We initiated our work by using *O*-methyl dioxime of the 5,15-dioxoporphyrin **Zn1a** and diphenylacetylene as the substrates in the presence of [Cp*RhCl_2_]_2_ (5 mol%), AgSbF_6_ (20 mol%), and Ag_2_O (2 equiv.) in DCE (1.0 mL) at 120 °C for 24 h (Table S1†). To our delight, the doubly pyridine-fused *anti*-quinoidal porphyrin **3aa** was afforded as a major product in 28% yield without the *syn* isomer (Table S1,† entry 1). Meanwhile, we isolated another product with a much larger polarity, the pyridinium-fused cation **4aa** with the SbF_6_^–^ counter anion, in 12% yield. Further optimization of reaction conditions improved the yield of **4aa** to 55% along with less than 10% yield of **3aa** (Table S1,† entry 9). The optimal catalytic system, comprising [Cp*RhCl_2_]_2_ (5 mol%) and AgSbF_6_ (20 mol%) in tetrahydrofuran (0.5 mL) at 100 °C for 24 h, afforded **3aa** in 66% yield (Table S1,† entry 17).

**Scheme 2:**
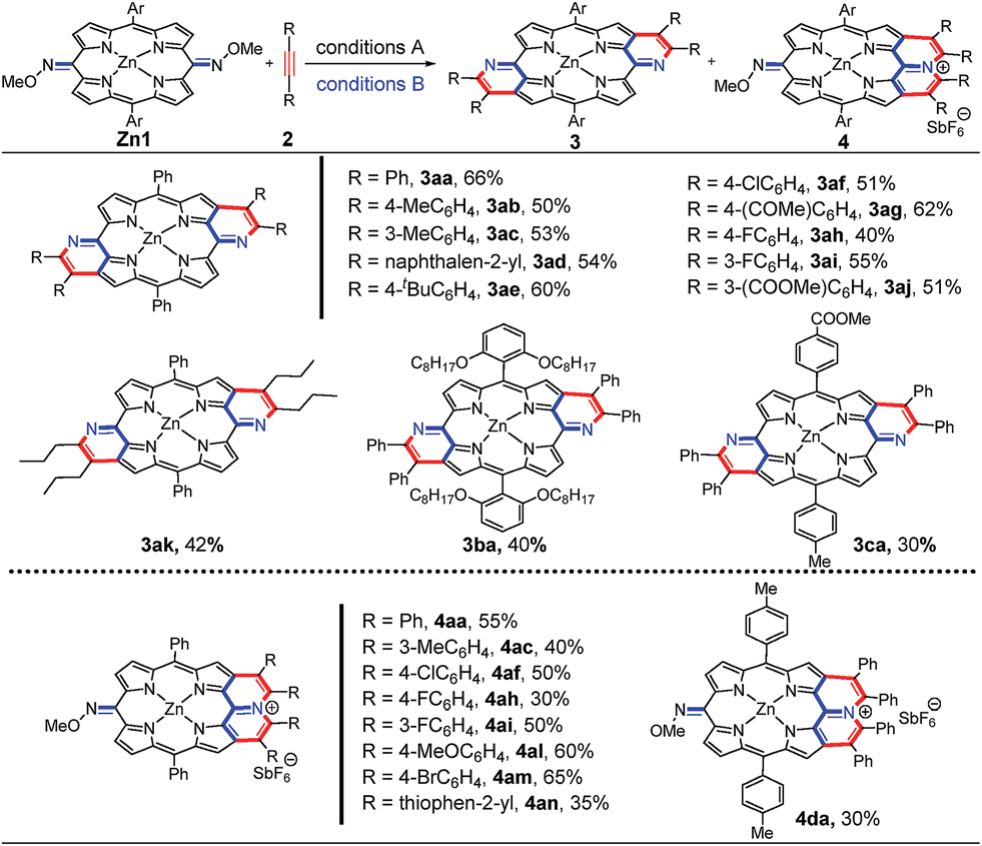
Substrate scope of reaction type A. Standard conditions A for **3**: **Zn1** (0.05 mmol), **2** (0.2 mmol), [Cp*RhCl_2_]_2_ (5 mol%), AgSbF_6_ (20 mol%), and THF (0.5 mL) at 100 °C under a N_2_ atmosphere for 24 h. Standard conditions B for **4**: **Zn1** (0.05 mmol), **2** (0.2 mmol), [Cp*RhCl_2_]_2_ (5 mol%), AgSbF_6_ (20 mol%), Ag_2_O (2.0 equiv.), NaSbF_6_ (2.0 equiv.), and DCE (0.5 mL) at 120 °C under a N_2_ atmosphere for 24 h.

With the optimized conditions for **3** and **4**, we explored the scope of internal alkynes and quinoidal porphyrins **Zn1** (Scheme 2). The annulation has relatively wide substrate scopes. Diaryl alkynes with either an electron-donating or electron-withdrawing group at the phenyl ring gave the desired products **3ab–3aj** in medium yields. The unsymmetrical **Zn1c** even gave the desired **3ca** with complete *anti*-selectivity. In addition, a family of pyridinium-fused **4** was assembled in the presence of the oxidant and NaSbF_6_. This protocol was compatible with both the electron-donating groups and electron-withdrawing halo substituent, delivering the desired products in medium yields. 1,2-Di(thiophen-2-yl)ethyne gave **4an** in 35% yield.

**Fig. 1:**
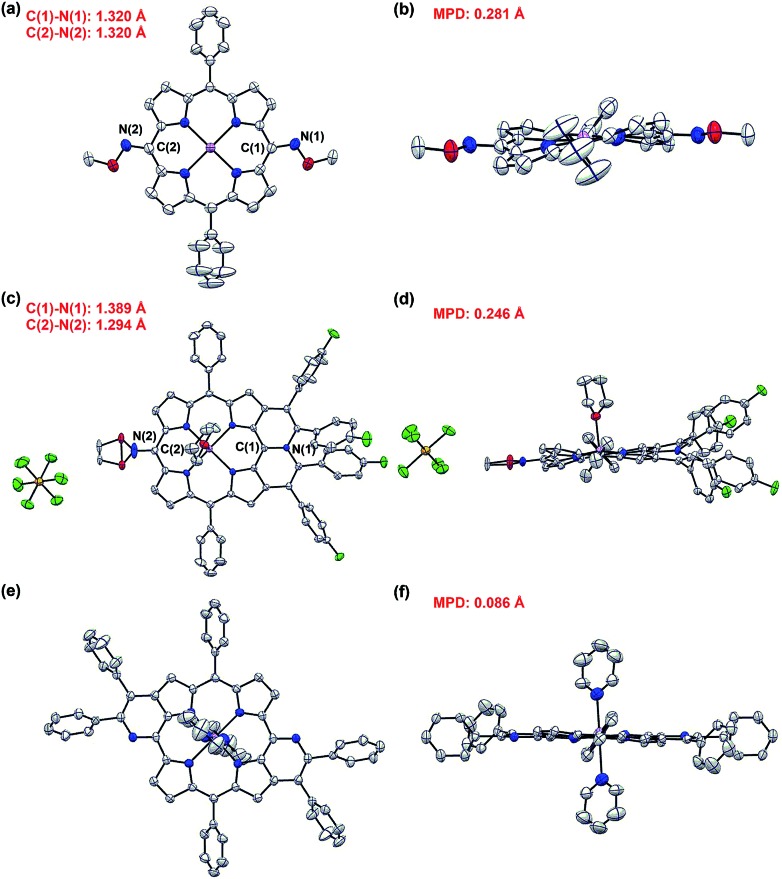
X-ray crystal structures of **Zn1a**, **4ah** and **3aa**. (a) Top view and (b) side view of **Zn1a**. (c) Top view and (d) side view of **4ah**. A splitting is observed due to the free rotation of the *O*-methyl oxime. (e) Top view and (f) side view of **3aa**. Solvent molecules (except axial ligands of Zn: tetrahydrofuran and pyridine) and all hydrogen atoms are omitted for clarity. The axial pyridine ligands of **3aa** are at 50% occupancy due to the crystallographically centrosymmetric structure. Thermal ellipsoids are shown at 50% probability.

The exact structures have been confirmed by single crystal X-ray analysis of **Zn1a**, **3aa** and **4ah**.^13^**Zn1a** exhibits a distorted configuration owing to its non-aromatic quinoidal conformation (Fig. 1a and b). In comparison, the conformation of **4ah** flattens slightly through π-conjugation between the pyridinium-fused moiety and quinoidal porphyrin framework (Fig. 1c and d). With the conjugative effect of two pyridine rings, **3aa** appears to be fairly coplanar (Fig. 1e and f). As a result, the mean plane deviation (MPD) values of **Zn1a**, **4ah** and **3aa** exhibit a gradually flattening trend.^14^ The C1–N1 double bond length of **4ah** is much longer than that of **Zn1a**, suggesting a weakened quinoidal character through the fusion of pyridinium rings.^12^

### Synthesis and characterization of doubly pyran-fused porphyrins

Although a great number of peripherally fused porphyrins have been synthesized, *meso-O*-containing heteroarene-fused porphyrins have not been reported yet. In this work, we attempted the reaction of the 5,15-dioxoporphyrin **Zn2a** and diphenylacetylene at 120 °C in the presence of [Cp*RhCl_2_]_2_ (5 mol%), AgSbF_6_ (20 mol%), and Ag_2_O (2 equiv.) in DCE (1.0 mL) (Table S2†). The doubly pyran-fused porphyrin **5aa** was isolated in 33% yield (Table S2,† entry 1). A mixture of *syn*- and *anti*-isomers was indicated by the ^1^H NMR spectrum, which clearly exhibits proportional signals for the three pairs of β protons. After extensive optimization of reaction conditions, the yield of **5aa** was improved to 60% (*syn* : *anti* = *ca.* 2 : 1) (Table S2,† entry 6). Unexpectedly, the single crystals of *syn*-**5aa** were obtained by recrystallization,^13^ which confirms the structural conversion from a quinoidal conformation to the stable porphyrin macrocycle.

**Scheme 3:**
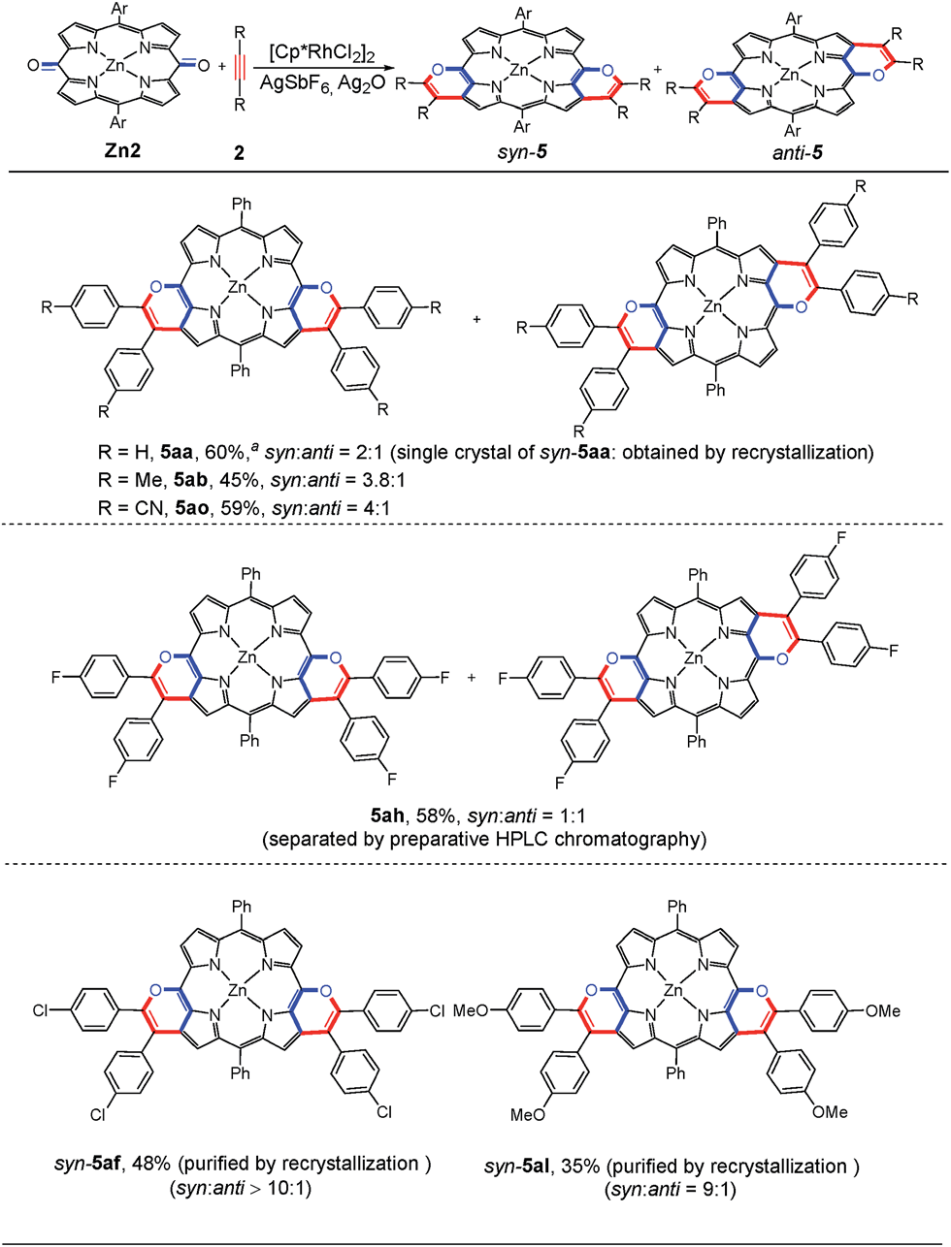
Substrate scope of reaction type B. Standard conditions: **Zn2** (0.05 mmol), **2** (0.2 mmol), [Cp*RhCl_2_]_2_ (5 mol%), AgSbF_6_ (20 mol%), Ag_2_O (2.0 equiv.), and DCE (0.5 mL) at 120 °C under a N_2_ atmosphere for 24 h. ^*a*^1,4-Dioxane was used as the solvent.

Subsequently, we investigated the scope of the substrates. As shown in Scheme 3, this annulation of **Zn2** with a variety of alkynes typically gives a mixture of *syn*- and *anti*-isomers. **5aa**, **5ab** and **5ao** were obtained with a relatively low regioselectivity ranging from 2 : 1 to 4 : 1 (*syn* : *anti*). Very interestingly, when the diaryl alkynes bear the chloro or methoxyl group, the target products **5af** and **5al** were formed with a large ratio of *syn*- and *anti*-isomers (up to >10/1). From these observations, we speculated that after the first annulation with the alkyne, the electron-rich character of the same side pyrrole ring could be further enhanced *via* the p–π conjugation effect between the chloro or methoxyl group and the phenyl ring. Thus, the second β-H cleavage tends to occur at the same side pyrrole ring. As a result, the *syn*-configuration is dominant in the [4 + 2] annulation process. Notably, a mixture of **5af** could be further purified by recrystallization to give a pure *syn*-**5af**, which was confirmed by ^1^H NMR and single crystal X-ray analysis (Table S10†). Recrystallization of **5al** could also afford pure *syn*-**5al**.

**Fig. 2:**
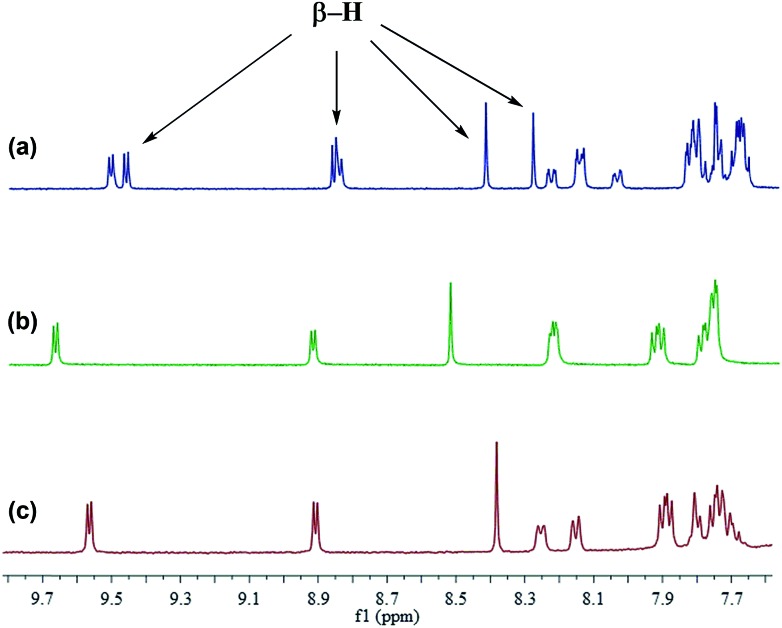
^1^H NMR spectra (400 MHz) of (a) the mixture of *anti* and *syn* isomers of **5ah**, (b) *anti*-**5ah**, and (c) *syn*-**5ah** in CDCl_3_.

In consideration of the presence of *syn*- and *anti*-configurations, we were naturally interested in their different optical and electronic properties. The *syn*- and *anti*-isomers of **5ah** were separated and purified by preparative HPLC chromatography (Fig. S1–S3†). Their characterization was then carried out by ^1^H NMR and IR spectroscopy. As shown in Fig. 2, the ^1^H NMR spectrum demonstrates that the mixture of **5ah** contains three pairs of β-H signals in the downfield region. It is worth indicating that the ^1^H NMR spectra of *syn*-**5ah** and *anti*-**5ah** cannot be simply overlapped with that of the mixture of **5ah**, which is probably due to the intermolecular π–π stacking interactions. The concentration-dependent ^1^H NMR spectral shifts of **5ah** in CDCl_3_ solution display such interactions (Fig. S4†). The IR spectra of the *syn*- and *anti*-configurations are also different (Fig. S5 and S6†).

**Fig. 3:**
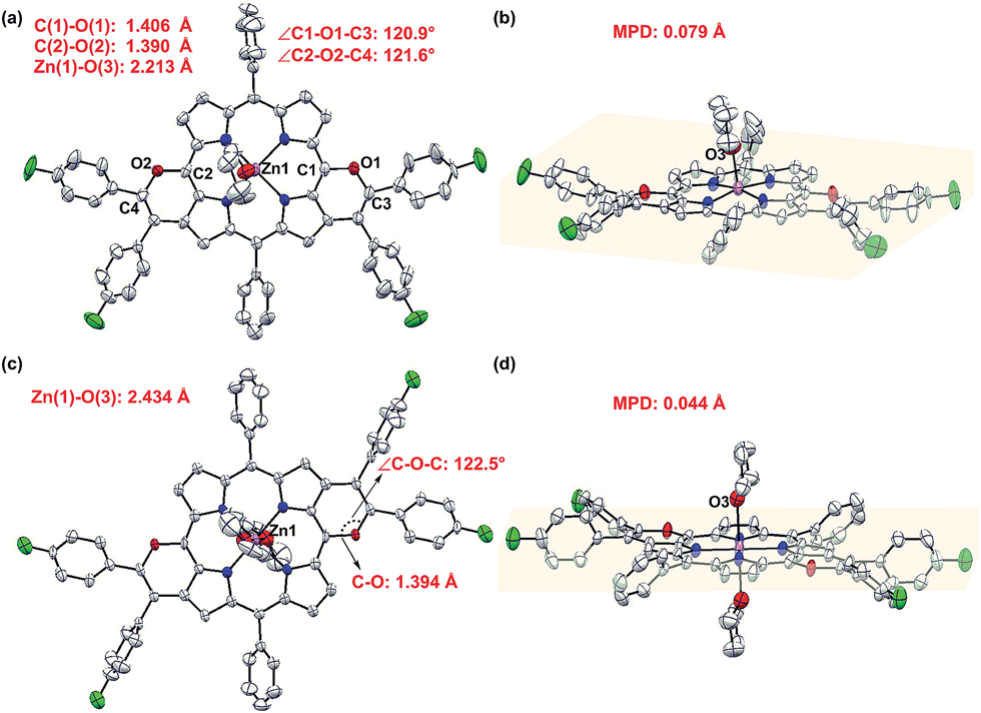
X-ray crystal structures of *syn*-**5ah** and *anti*-**5ah**. (a) Top view and (b) side view of *syn*-**5ah**. (c) Top view and (d) side view of *anti*-**5ah**. Thermal ellipsoids are shown at 50% probability. Solvent molecules (except axial ligands of Zn: tetrahydrofuran) and all hydrogen atoms are omitted for clarity. THF ligands of *anti*-**5ah** are at 50% occupancy due to the crystallographically centrosymmetric structure.

Single crystal X-ray diffractometry of *syn*-**5ah** or *anti*-**5ah** confirmed the spectroscopically derived connectivity of the two regioisomers and proved them both to be essentially planar, with only minor differences in the mean plane deviation of their C_24_N_4_O_2_Zn chromophores (Fig. 3).^14^ Slight differences in the metric parameters of the frameworks of both derivatives highlight the small structural effects of the two different orientations of two pyran rings, although they are, as shown above, electronically most distinct from each other.

### Theoretical calculations on aromatic delocalization

**Scheme 4:**
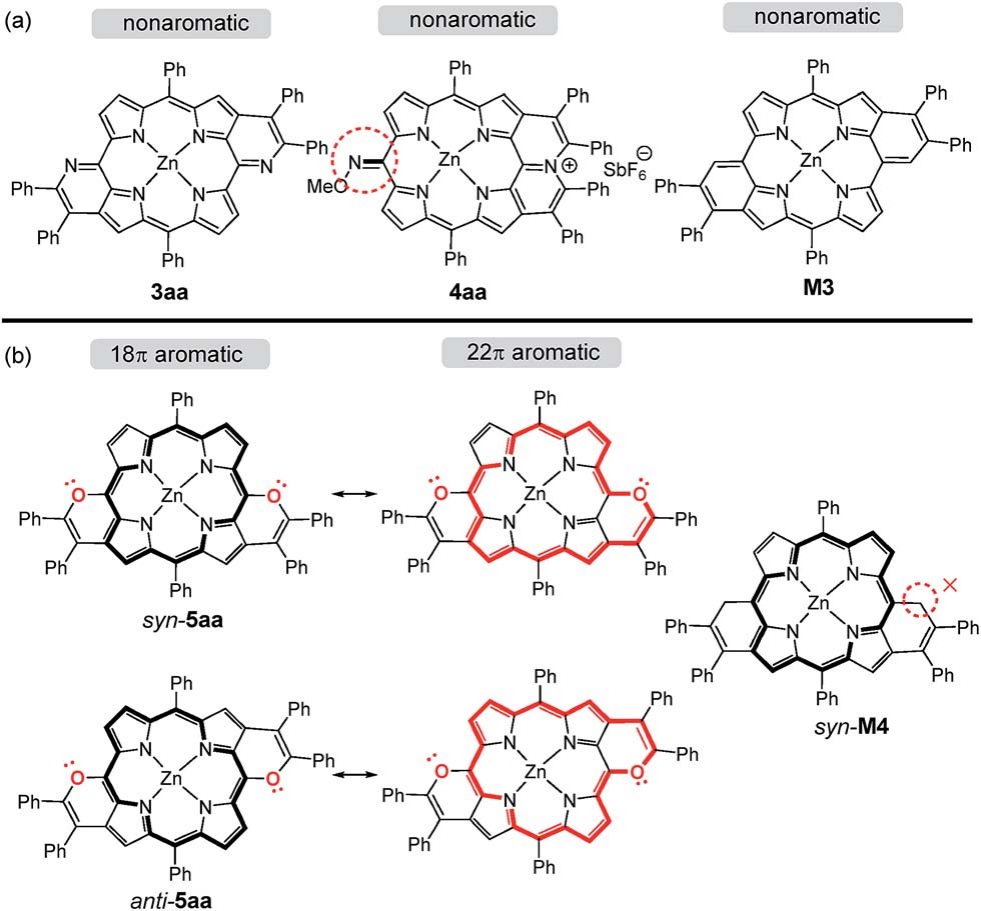
The aromaticity of **3aa**, **4aa**, simulated **M3** and *syn*-**M4**, and the resonance structures of *syn*-**5aa** and *anti*-**5aa**.

**Fig. 4:**
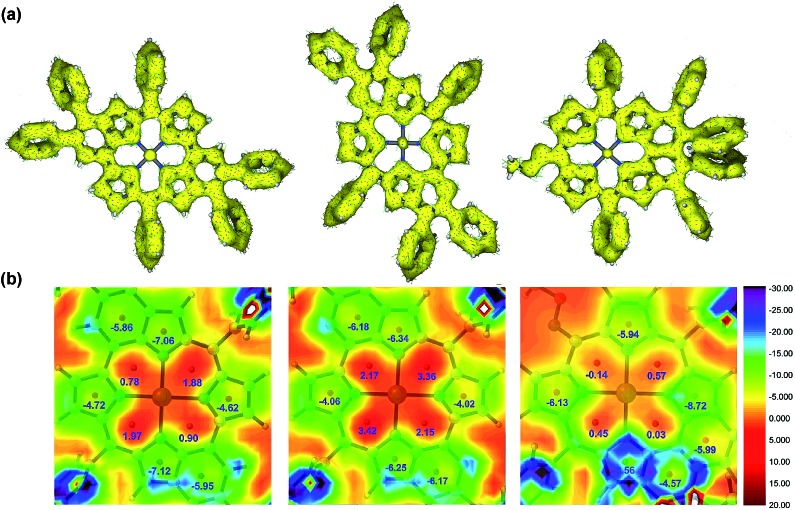
(a) ACID plots of **M3** (left), **3aa** (middle), and **4aa** (right). (b) NICS(1) value distribution diagrams of **M3** (left), **3aa** (middle), and **4aa** (right). The given values represent the calculated NICS(1) values of each ring.

To gain insight into the changes of the aromatic contribution caused by the peripherally fused-heteroarenes, we calculated the anisotropy of the induced current density (ACID) and the nucleus-independent chemical shift (NICS) of porphyrins **3aa**, **4aa**, and *syn*- and *anti*-**5aa**. In the meantime, the *anti*-quinoidal porphyrin **M3** and porphyrin *syn*-**M4** were simulated and calculated as references (Scheme 4). **M3** is assigned to be nonaromatic due to Clar's sextet of peripheral benzenes, which is similar to the reported quinoidal porphyrin.^15^ The calculated NICS(1) values of **3aa** demonstrate the local aromaticity of two pyridine rings, resulting in its nonaromatic character being similar to that of **M3** (Fig. 4). The approximate zero NICS(1) values of **4aa** reveal the nonaromatic character.

**Fig. 5:**
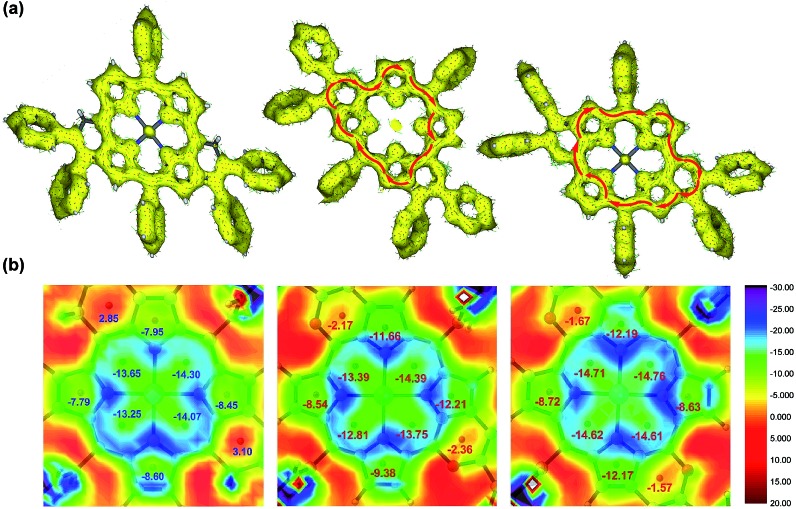
(a) ACID plots of *syn*-**M4** (left), *syn*-**5aa** (middle), and *anti*-**5aa** (right). (b) NICS(1) value distribution diagrams of *syn*-**M4** (left), *syn*-**5aa** (middle), and *anti*-**5aa** (right). The given values represent the calculated NICS(1) values of each ring.

Differing by the appearance of a disrupted node at the exocyclic methene bonds of *syn*-**M4**, both *syn*- and *anti*-**5aa** exhibit a strong clockwise current density flow, which can be drawn as a 22π conjugation pathway (Fig. 5). The calculated NICS(1) values in the inner core region of *anti*-**5aa** are more negative than those of *syn*-**5aa**, suggesting the enhanced aromatic contribution by peripheral *anti* fusion. These results further account for the more downfield shifted β-H signals of *anti*-**5ah** than of *syn*-**5ah** in the ^1^H NMR spectra shown in Fig. 2.5*b* Notably, the two pyrrole rings adjacent to the fused pyran moieties show more negatively shifted NICS(1) values than the others. These results demonstrate that the weak aromatic contribution of fused pyran moieties is non-negligible and finally enhances the 22π-electron conjugated circuit through the p–π conjugation effect between the *meso*-oxygen atom and the porphyrin macrocycle (Scheme 4).

### Synthesis of the pyrylium-fused porphyrin dimer

**Scheme 5:**
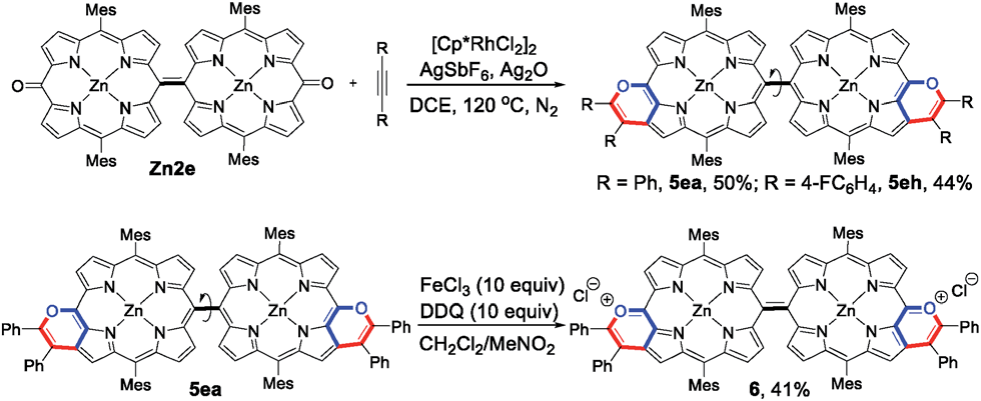
Synthesis of the porphyrin dimers **5ea** and **5eh** and oxonium dication **6**.

As an extension, the 5,15-dioxoporphyrin dimer **Zn2e** was prepared^16^ and could undergo annulation with alkynes, delivering the doubly pyran-fused porphyrin dimers **5ea** and **5eh** in 50% and 44% yields, respectively (Scheme 5). Notably, **5ea** and **5eh** displayed a single configuration due to the rotation of the exocyclic C–C linkage. Treatment of **5ea** with FeCl_3_ and 2,3-dichloro-5,6-dicyano-1,4-benzoquinone (DDQ) afforded a sole product, in which the two pyran rings were oxidized to deliver the doubly pyrylium-fused porphyrin dimer **6**. The ESI mass spectrum of **6** exhibited its parent ion peak at *m*/*z* 799.2404 (calcd for C_104_H_78_N_8_O_2_Zn_2_, *m*/*z* 799.2410 [M]^+^), corresponding to its quinoidal oxonium dication.

### Optical and electronic properties

**Fig. 6:**
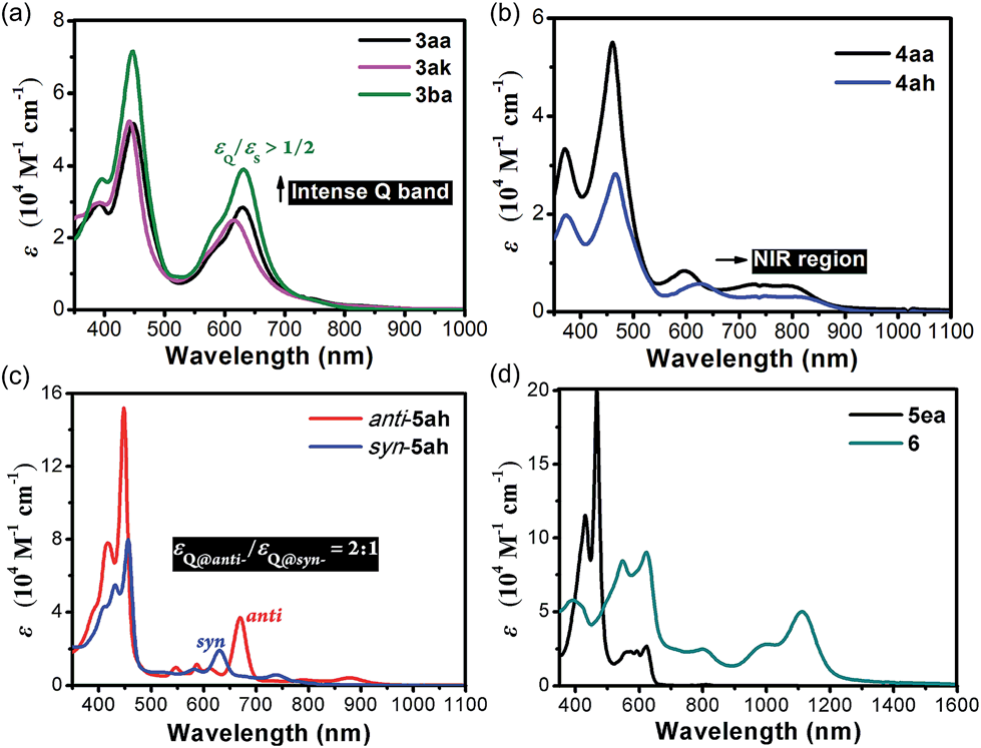
The UV-vis-NIR absorption spectra of **3** (a) and **4** (b), *syn*-/*anti*-**5ah** (c), and **5ea** and **6** (d) in CH_2_Cl_2_ solution (4.0 × 10^–5^ M for **3** and **4**; 1.0 × 10^–5^ M for **5ah**, **5ea** and **6**) *ε* = extinction coefficient.

The absorption and emission spectra were measured in chromatographically pure CH_2_Cl_2_ (Fig. 6 and Tables S3 and S4†). **3** show quite intense Q-band absorption with a ratio of *ε*_Q_/*ε*_S_ up to over 1/2. The cations **4** have significantly red-shifted Q bands expanding up to 900 nm. Through the fusion of pyran rings, *syn*-**5ah** and *anti*-**5ah** show red-shifted Q-band absorption spectra, with a weak absorption tail up to 900 nm (Fig. 6c), indicating that their conjugated π-electron systems are largely perturbed by peripheral moieties. The intensity of the Q-band absorption of *anti*-**5ah** is about twice that of *syn*-**5ah**. **5ea** shows broader and enhanced Q bands (Fig. 6d). After further oxidation, the oxonium dication **6** exhibits Soret bands at 548 and 622 nm with an intense red-shifted Q band centered at 1113 nm, which demonstrates a great ability to absorb both the visible and near-infrared light up to approximately 1300 nm.

**Fig. 7:**
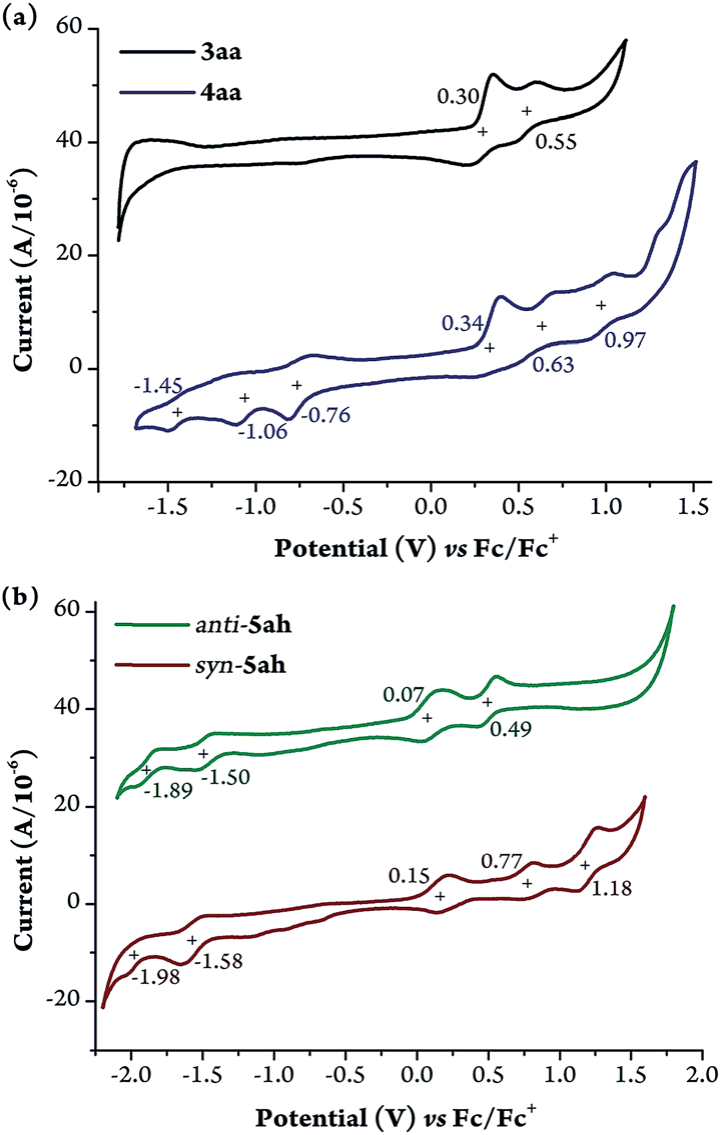
Cyclic voltammograms of **3aa**, **4aa**, and *syn*- and *anti*-**5ah**. (a) **3aa** and **4aa** in CH_3_CN. (b) *syn*- and *anti*-**5ah** in CH_2_Cl_2_. *n*-Bu_4_NPF_6_ (0.1 M) was chosen as the supporting electrolyte, and Ag/AgCl was chosen as the reference electrolyte. The ferrocene/ferrocenium ion couple was employed as an external reference. Scan rate: 50 mV s^–1^.

Cyclic voltammetric studies were then performed under N_2_ (Fig. 7 and Table S5; for details, see the ESI†). **3aa** showed two quasi-reversible oxidation waves without any reduction wave (Fig. 7a). In contrast, **4aa** exhibited three positive oxidation potentials and three reduction waves. Both *syn* and *anti*-**5ah** displayed reversible oxidation waves and two quasi-reversible reduction waves (Fig. 7b). The smaller HOMO–LUMO gap for *anti*-**5ah** (1.57 eV *vs.* 1.73 eV) just accounts for the more red-shifted Q-band absorption of *anti*-**5ah** than of *syn*-**5ah** (Fig. 6).

### Singlet oxygen quantum yields (*Φ*_Δ_) in the red/NIR Q-band region

Photodynamic therapy (PDT), a combination of a photosensitizer, light, and molecular oxygen (^3^O_2_), has been proved to be an emerging and noninvasive therapeutic technique for cancers and other benign diseases.^17^,^18^ Considering that the pyridine/pyran-fused porphyrins exhibit enhanced Q-band absorption in the red/NIR region, we were interested in their potential as the photosensitizer in the body's therapeutic window (650–900 nm). After serial screening, *syn*-**5af** and *syn*-**5al** exhibited a great ability to sensitize ^1^O_2_ (for details, see ESI Fig. S7–S11†). While the *Φ*_Δ_ value of methylene blue (**MB**, as the standard) was found to be 0.52 in DMF, the *Φ*_Δ_ values of *syn*-**5af** and *syn*-**5al** were calculated to be 0.54 and 0.63, respectively. Furthermore, *syn*-**5al** exhibits a great ability to sensitize ^1^O_2_ with a *Φ*_Δ_ value of 0.61 when irradiated with a 680 nm laser beam.

## Conclusions

In summary, we have established a rhodium-catalyzed [4 + 2] annulation strategy to rapidly construct pyridine-fused *anti*-quinoidal porphyrins **3**, pyridinium-fused cations **4**, and doubly pyran-fused porphyrins **5**, which exhibit controllable chemoselectivity and regioselectivity. Further oxidation of the expanded porphyrin dimer **5ea** delivers the oxonium dication **6** with intense near-infrared absorption up to 1300 nm. Theoretical investigation based on the ACID and NICS(1) values reveals the 22π aromatic (*vs.* 18π aromatic) character of pyran-fused porphyrin (*syn*/*anti*-**5aa**) caused by the π/p–π conjugated effect between the macrocyclic core and *meso*-heteroarene. Compared with the commercially available **MB**, *syn*-**5al** exhibits a better ability to sensitize ^1^O_2_ when irradiated with a 680 nm laser beam, and exhibits potential as a PDT photosensitizer in the body's therapeutic window. The straightforward access for discovering organic functional molecules developed herein has well exemplified the charm of C–H activation.

## Conflicts of interest

There are no conflicts to declare.

## Supplementary Material

Supplementary information

Crystal structure data
