# Supplementary material for: An unusual [4 + 2] fusion strategy to forge meso-N/O-heteroarene-fused (quinoidal) porphyrins with intense near-infrared Q-bands
Source: Chem Sci. 2019 Jun 17;10(30):7274–80. doi: 10.1039/c9sc01596e (PMC6686728; doi:10.1039/c9sc01596e)

Electronic Supplementary Material (ESI) for Chemical Science.
This journal is © The Royal Society of Chemistry 2019

Supporting Information

Unusual [4+2] Fusion Strategy to Forge *meso-N/O*-Heteroarene-Fused (Quinoidal) Porphyrins with Intense Near-Infrared Q-Bands

Chengming Li, Lei Zhu, Wenbo Liang, Rongchuan Su, Jiangliang Yin, Yanmei Hu, Yu Lan, Di Wu, and Jingsong You**

[†]Key laboratory of Green Chemistry and Technology of Ministry of Education, College of Chemistry, Sichuan University, 29 Wangjiang Road, Chengdu 610064, P. R. China

[‡]School of Chemistry and Chemical Engineering, and Chongqing Key Laboratory of Theoretical and Computational Chemistry, Chongqing University, Chongqing 400030, P. R. China

E-mail: *jsyou@scu.edu.cn; wood@scu.edu.cn*

Table of contents

I. General remarks..S3

II. Optimization of the oxidative annulation of *O*-methyl dioximes of 5,15-dioxoporphyrins **Zn1** with alkynes...S3

III. General procedure for the synthesis of doubly pyridine-fused *anti*-quinoidal porphyrins **3**..S5

IV. General procedure for the synthesis of pyridinium-fused cations **4**...........S5

V. Optimization of the oxidative annulation of 5,15-dioxoporphyrins **Zn2** with alkynes..S6

VI. General procedure for the preparation of **Zn1** and **Zn2**............................S7

VII. Preparation and characterization of the doubly pyridine-fused anti-quinoidal porphyrins **3** and pyridinium-fused porphyrin cations **4**………S12

VIII. Preparation and characterization of the doubly pyran-fused porphyrins **5** and pyrylium-fused porphyrin dimer **6**...S23

IX. HPLC chromatograph for separation of *syn*-**5ah** and *anti*-**5ah**................S30

X. The concentration-dependent ^{1}H NMR spectra of the mixture of **5ah**…...S31

XI. FI-IR spectra of *syn*-**5ah** and *anti*-**5ah**..S32

XII. DFT calculation detail...S32

XIII. Optical properties of the representative products.................................S41

XIV. Cyclic voltammogram measurements...S42

XV. Quantification of singlet oxygen generation...S43

XVI. Single crystal X-ray structures of **Zn1a**, **3aa**, **4ah**, *syn*-**5aa**, *syn*-**5af**, *syn*- and *anti*-**5ah**..S46

XVII. References...S53

XVIII. Copies of NMR spectra...S55

I. General remarks

NMR spectra were recorded on a Varian Inova 400, or a Bruker AV II-400 spectrometer. The ^{1}H NMR (400 MHz) chemical shifts were recorded relative to $CDCl_3$, CD_2Cl_2, TMS or DMSO-d_6 as the internal reference ($CDCl_3$: δ_H = 7.26 ppm; CD_2Cl_2: δ_H = 5.32 ppm; TMS: δ_H = 0.00 ppm ; DMSO-d_6: δ_H = 2.50 ppm). The solubility of the product proved too low in common organic solvents to allow the ^{13}C NMR spectrum to be recorded. High-resolution mass spectra (HRMS) were obtained with a Shimadzu LCMS-IT-TOF (ESI). IR spectra were obtained with NEXUS670FT-IR. X-Ray single-crystal diffraction data were collected on an Oxford Xcalibur E single crystal diffractometer or an Agilent Technologies Gemini plus single crystal diffraction. UV/Vis spectra were measured on a HITACHI U-2910. Fluorescence spectra and absolute quantum yields were collected on a Horiba Jobin Yvon-Edison Fluoromax-4 fluorescence spectrometer with a calibrated integrating sphere system.

Unless otherwise noted, all reagents were obtained from commercial suppliers and used without further purification. $[Cp^*RhCl_2]_2$[1] and alkynes were prepared according to the literature procedures. The solvents were purified and dried using an Innovative Technology PS-MD-5 Solvent Purification System. $RhCl_3 \cdot xH_2O$ were purchased from Shanxi Kaida Chemical Engineering (China) CO., Ltd. $AgSbF_6$ was purchased from Alfa Aesar.

II. Optimization of the oxidative annulation of *O*-methyl dioximes of 5,15-dioxoporphyrins Zn1 with alkynes

A schlenk tube with a magnetic stir bar was charged with metal complex (2.5 μmol, 5.0 mol %), $AgSbF_6$ (10 μmol, 20 mol %), oxidant, additive, *O*-methyl dioxime of 5,15-dioxoporphyrin **Zn1a** (27.8 mg, 0.05 mmol), diphenylacetylene (35.6 mg, 0.2 mmol), and solvent under N_2 atmosphere. The resulting solution was stirred at room temperature for 10 min and then at the indicated temperature for 24 h. After being cooled down, the reaction mixture was filtered through a Celite pad, and then washed with 30 mL of CH_2Cl_2. The

solvent of the filtrate was removed under reduced pressure. The purification was performed by column chromatography on neutral alumina (petroleum ether/ethyl acetate = 8:1 to 4:1) to provide **3aa** and further by silica gel (petroleum ether/ethyl acetate = 1:1 to 1:3, v/v) to provide **4aa**, respectively.

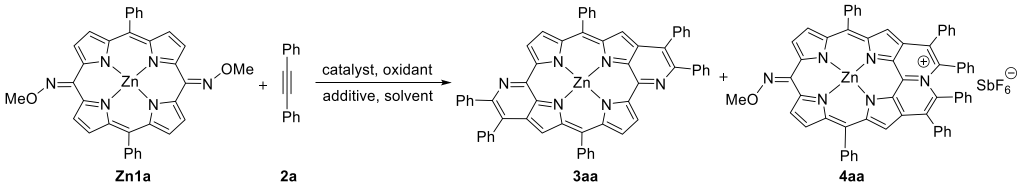

Table S1. Optimization for the synthesis of doubly pyridine-fused *anti*-quinoidal porphyrins **3** and pyridinium-fused cations **4**[a]

Entry	Catalyst	Oxidant (equiv)	Additive (equiv)	Solvent (mL)	Yield of **3aa** (%)[b]	Yield of **4aa** (%)[b]
1	$[Cp^*RhCl_2]_2$	Ag_2O (2.0)	-	DCE (1.0)	28	12
2[c]	$[Cp^*RhCl_2]_2$	Ag_2O (2.0)	-	DCE (1.0)	ND	ND
3	$[Cp^*RhCl_2]_2$	Ag_2O (2.0)	$NaSbF_6$ (2.0)	DCE (1.0)	15	37
4	$[Cp^*RhCl_2]_2$	Ag_2O (2.0)	$Zn(OTf)_2$ (2.0)	DCE (1.0)	26	trace
5	$[Cp^*RhCl_2]_2$	Ag_2O (2.0)	$NaSbF_6$ (2.0)	1,4-dioxane (1.0)	21	17
6	$[Cp^*RhCl_2]_2$	Ag_2O (2.0)	$NaSbF_6$ (2.0)	THF (1.0)	35	trace
7	$[Cp^*RhCl_2]_2$	Ag_2O (2.0)	$NaSbF_6$ (2.0)	toluene (1.0)	33	trace
8	$[Cp^*RhCl_2]_2$	Ag_2O (2.0)	$NaSbF_6$ (2.0)	DMF (1.0)	18	trace
**9**	**$[Cp^*RhCl_2]_2$**	**Ag_2O (2.0)**	**$NaSbF_6$ (2.0)**	**DCE (0.5)**	**<10**	**55**
10	$[Cp^*RhCl_2]_2$	Ag_2O (3.0)	$NaSbF_6$ (2.0)	DCE (0.5)	<10	55
11	$[Cp^*RhCl_2]_2$	Ag_2O (1.0)	$NaSbF_6$ (2.0)	DCE (0.5)	<10	41
12	$[Cp^*RhCl_2]_2$	Ag_2CO_3 (2.0)	$NaSbF_6$ (2.0)	DCE (0.5)	13	45
13	$[Cp^*RhCl_2]_2$	AgOAc (2.0)	$NaSbF_6$ (2.0)	DCE (0.5)	21	trace
14	$[Cp^*IrCl_2]_2$	Ag_2O (2.0)	$NaSbF_6$ (2.0)	DCE (0.5)	ND	ND
15	$[Cp^*RhCl_2]_2$	-	-	THF (1.0)	52	-
16	$[Cp^*RhCl_2]_2$	-	-	THF (0.5)	52	-
**17[d]**	**$[Cp^*RhCl_2]_2$**	**-**	**-**	**THF (0.5)**	**66**	**-**
18[e]	$[Cp^*RhCl_2]_2$	-	-	THF (0.5)	ND	-

[a]Reaction conditions: **Zn1a** (0.05 mmol), **2a** (0.2 mmol), catalyst (5 mol %), $AgSbF_6$ (20 mol %), oxidant, additive, and solvent at 120 ^oC under N_2 for 24 h. [b]Isolated yield. [c]$AgSbF_6$ was not added. [d]The reaction was carried out at 100 ^oC. [e]The reaction was carried out at 80 ^oC.

III. General procedure for the synthesis of doubly pyridine-fused *anti*-quinoidal porphyrins 3

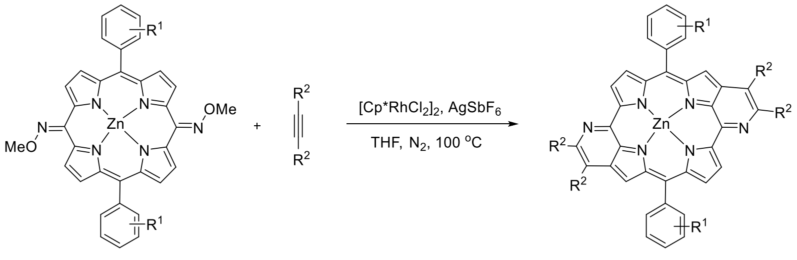

A schlenk tube with a magnetic stir bar was charged with $[Cp^*RhCl_2]_2$ (1.5 mg, 2.5 μmol), $AgSbF_6$ (3.4 mg, 10 μmol), *O*-methyl dioximes of 5,15-dioxoporphyrins **Zn1** (0.05 mmol), alkyne (0.2 mmol), and THF (0.5 mL) under N_2 atmosphere. The resulting solution was stirred at room temperature for 10 min and then at 100 °C for 24 h. After being cooled down, the reaction mixture was filtered through a Celite pad, and then washed with 30 mL of CH_2Cl_2. The solvent of the filtrate was removed under reduced pressure. The purification was performed by column chromatography on neutral alumina to provide the desired product **3**.

IV. General procedure for the synthesis of pyridinium-fused cations 4

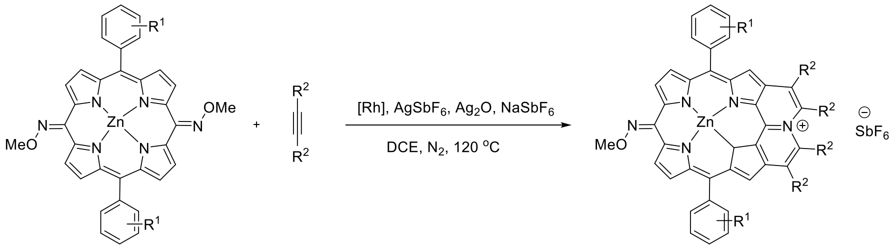

A schlenk tube with a magnetic stir bar was charged with $[Cp^*RhCl_2]_2$ (1.5 mg, 2.5 μmol), $AgSbF_6$ (3.4 mg, 10 μmol), *O*-methyl dioximes of 5,15-dioxoporphyrins **Zn1** (0.05 mmol), alkyne (0.2 mmol), Ag_2O (23.2 mg, 0.1 mmol), $NaSbF_6$ (25.9 mg, 0.1 mmol), and DCE (0.5 mL) under N_2 atmosphere. The resulting solution was stirred at room temperature for 10 min and then at 120 °C for 24 h. After being cooled down, the reaction mixture was filtered through a Celite pad, and then washed with 30 mL of CH_2Cl_2. The solvent of the filtrate was removed under reduced pressure. The purification was

performed by column chromatography on silica gel to provide the desired product **4**.

V. Optimization of the oxidative annulation of 5,15-dioxoporphyrins Zn2 with alkynes

A schlenk tube with a magnetic stir bar was charged with metal complex (2.5 μmol, 5.0 mol %), $AgSbF_6$ (10 μmol, 20 mol %), oxidant, additive, 5,15-dioxoporphyrin **Zn2a** (27.8 mg, 0.05 mmol), diphenylacetylene (35.6 mg, 0.2 mmol), and solvent under N_2 atmosphere. The resulting solution was stirred at room temperature for 10 min and then at the indicated temperature for 24 h. After being cooled down, the reaction mixture was filtered through a Celite pad, and then washed with 30 mL of CH_2Cl_2. The solvent of the filtrate was removed under reduced pressure. The purification was performed by column chromatography on neutral alumina (petroleum ether/ethyl acetate = 10:1 to 5:1, v/v) to provide **5aa** (a mixture of *syn-* and *anti*-isomers).

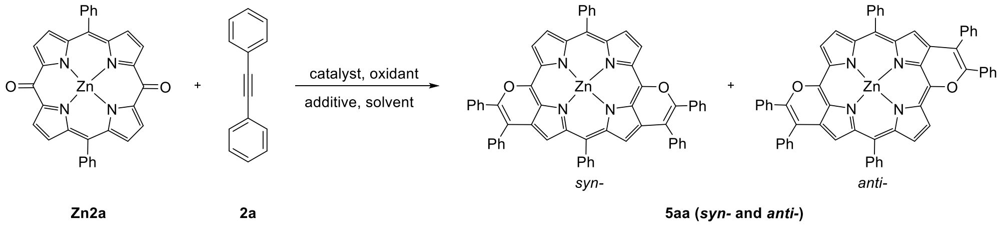

Table S2. Optimization for the synthesis of doubly pyran-fused porphyrin[a]

Entry	Catalyst	Oxidant (equiv)	Additive (equiv)	Solvent (mL)	Yield[b] (%)
1	$[Cp^*RhCl_2]_2$	Ag_2O (2.0)	-	DCE (1.0)	33
2[c]	$[Cp^*RhCl_2]_2$	Ag_2O (2.0)	-	DCE (1.0)	ND
**3**	**$[Cp^*RhCl_2]_2$**	**Ag_2O (2.0)**	**-**	**DCE (0.5)**	**46**
4[d]	$[Cp^*RhCl_2]_2$	Ag_2O (2.0)	-	DCE (0.5)	35
5	$[Cp^*RhCl_2]_2$	Ag_2O (2.0)	-	DMF (0.5)	trace
**6**	**$[Cp^*RhCl_2]_2$**	**Ag_2O (2.0)**	**-**	**1,4-dioxane (0.5)**	**60**
7	$[Cp^*RhCl_2]_2$	Ag_2O (2.0)	-	THF (0.5)	< 10
8	$[Cp^*RhCl_2]_2$	Ag_2O (2.0)	-	toluene (0.5)	22
9	$[Cp^*RhCl_2]_2$	AgOAc (2.0)	-	DCE (0.5)	trace
10	$[Cp^*RhCl_2]_2$	$AgNO_3$ (2.0)	-	DCE (0.5)	ND
11	$[Cp^*RhCl_2]_2$	Ag_2CO_3 (2.0)	-	DCE (0.5)	42
12	$[Cp^*RhCl_2]_2$	Ag_2O (2.0)	PivOH (2.0)	DCE (0.5)	<10
13	$[Cp^*RhCl_2]_2$	Ag_2O (2.0)	NaOAc (2.0)	DCE (0.5)	28

14	$[Cp^*RhCl_2]_2$	Ag_2O (2.0)	$NaSbF_6$ (2.0)	DCE (0.5)	18
15	$[Cp^*RhCl_2]_2$	Ag_2O (3.0)	-	DCE (0.5)	46
16	$[Cp^*IrCl_2]_2$	Ag_2O (2.0)	-	DCE (0.5)	ND

[a]Reaction conditions: **Zn2a** (0.05 mmol), **2a** (0.2 mmol), $[Cp^*RhCl_2]_2$ (5 mol %), $AgSbF_6$ (20 mol %), oxidant, additive, and solvent at 120 °C under N_2 for 24 h. [b]Isolated yield. [c]$AgSbF_6$ was not added. [d]The reaction was carried out at 140 °C.

VI. General procedure for the preparation of Zn1 and Zn2

H₂DPP 1, 5,15-dioxoporphyrin **Zn2a**, **Zn2d**, and 5,15-dioxoporphyrin dimer **Zn2e** were prepared according to the literature procedures.[2-4] 5,15-Diarylporphyrin was prepared according to the literatures.[5] For the preparation of **H_2DPP 2**, *O*-methyl dioximes of 5,15-dioxoporphyrins **Zn1** and 5,15-dioxoporphyrin **Zn2**, the following procedure was used.

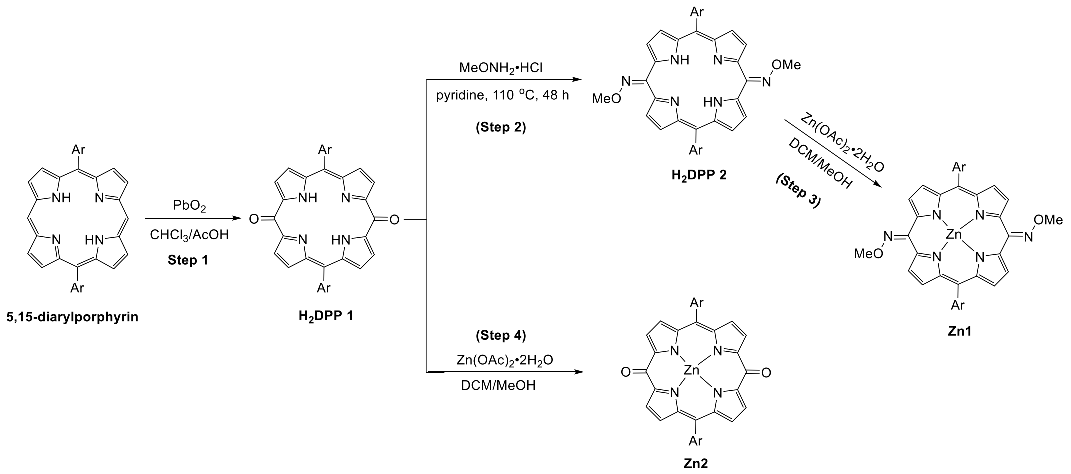

Step 1[2]: 5,15-Diarylporphyrin (0.24 mmol) was dissolved in a mixture of $CHCl_3$ (14 mL) and AcOH (2.9 mL). A solution of PbO_2 (0.57 g, 2.4 mmol) was added and the reaction mixture was stirred for 5 h open to air, whereafter the mixture was slowly poured into a saturated aqueous sodium bicarbonate solution (45 mL) to quench the AcOH. The aqueous layer was extracted twice with CH_2Cl_2 (2 × 40 mL), and the two organic fractions were also passed through the Celite. The combined organic filtrates were evaporated and the residue was purified by column chromatography on silica gel to afford the desired product **H_2DPP 1** as a black solid.

Step 2[6]: A suspension of **H_2DPP 1** (0.10 mmol) above and $MeONH_2$·HCl

(83.5 mg, 1.0 mmol) in pyridine (2.0 mL) was stirred at 110 ^oC for 48 h. Then pyridine was removed under reduce pressure and CH_2Cl_2 (30 mL) was added to dilute it. The mixture was washed with water and dried over anhydrous $MgSO_4$. The purification was performed by column chromatography on silica gel to provide the desired product **H_2DPP 2**.

Step 3[5]: A suspension of **H_2DPP 2** above and $Zn(OAc)_2{\cdot}2H_2O$ (10 equiv) in a mixture of CH_2Cl_2 (12 mL) and MeOH (6 mL) was stirred at 23 ^oC for 3 h. The reaction was quenched with water (30 mL), and the mixture was extracted with CH_2Cl_2 (3 × 30 mL). The combined extracts were washed with water and dried over anhydrous $MgSO_4$. The solvent was removed under reduce pressure to give the product *O*-methyl dioximes of 5,15-dioxoporphyrins **Zn1**. The yield of step 3 was near 100%.

Step 4[5]: A suspension of **H_2DPP 1** above and $Zn(OAc)_2{\cdot}2H_2O$ (10 equiv) in a mixture of CH_2Cl_2 (24 mL) and MeOH (12 mL) was stirred at 23 ^oC for 3 h. The reaction was quenched with water (30 mL), and the mixture was extracted with CH_2Cl_2 (3 × 30 mL). The combined extracts were washed with water and dried over anhydrous $MgSO_4$. The solvent was removed under reduce pressure to give the product 5,15-dioxoporphyrin **Zn2**.

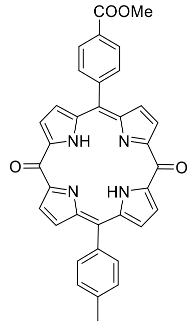

H_2DPP 1c: Following the synthetic procedure step 1. 5-(4-methoxycarbonylphenyl)-15-(4-methylphenyl)porphyrin[7] (128.3 mg, 0.24 mmol) was used. Purification via column chromatography on silica gel (petroleum ether/ CH_2Cl_2 = 1:1 to 1:3, v/v) afforded **H_2DPP 1c** as a black solid (80.1 mg, 59% yield). ^{1}H NMR ($CDCl_3$, 400 MHz): δ (ppm) 13.95 (d, J = 14.0 Hz, 2H), 8.15 (d, J = 8.4 Hz, 2H), 7.52 (d, J = 8.4 Hz, 2H), 7.26-7.32 (m, 4H),

7.15 (d, J = 4.4 Hz, 4H), 6.48 (d, J = 48.4 Hz, 4H), 3.99 (s, 3H), 2.46 (s, 3H). HRMS (ESI$^+$): calcd for $C_{35}H_{25}N_4O_4$ [M+H]$^+$ 565.1870, found 565.1872.

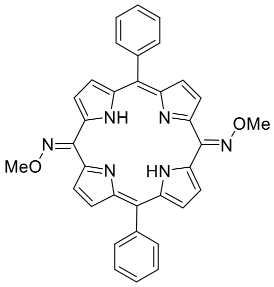

H_2DPP 2a: Following the synthetic procedure step 2. **H_2DPP 1a** (49.2 mg, 0.10 mmol) was used. Purification via column chromatography on silica gel (petroleum ether/ethyl acetate = 8:1, v/v) afforded **H_2DPP 2a** as a red solid (50.4 mg, 91% yield). ^{1}H NMR ($CDCl_3$, 400 MHz): δ (ppm) 12.41 (s, 2H), 7.42-7.46 (m, 12H), 7.15 (d, J = 3.6 Hz, 2H), 6.77 (d, J = 3.6 Hz, 2H), 6.32 (s, 2H), 4.20 (s, 6H). HRMS (ESI$^+$): calcd for $C_{34}H_{27}N_6O_2$ [M+H]$^+$ 551.2190, found 551.2187.

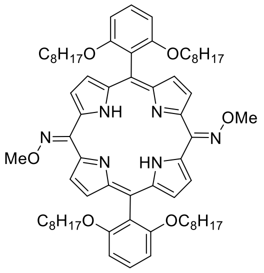

H_2DPP 2b: Following the synthetic procedure step 2. **H_2DPP 1b** (100.5 mg, 0.10 mmol) was used. Purification via column chromatography on silica gel (petroleum ether/ethyl acetate = 40:1, v/v) afforded **H_2DPP 2b** as a red solid (82.9 mg, 78% yield). ^{1}H NMR ($CDCl_3$, 400 MHz): δ (ppm) 12.47 (s, 2H), 7.42 (d, J = 4.4 Hz, 2H), 7.33 (t, J = 8.4 Hz, 2H), 7.13 (d, J = 4.8 Hz, 2H), 6.75 (d, J = 4.4 Hz, 2H), 6.62 (d, J = 8.4 Hz, 4H), 6.27-6.29 (m, 2H), 4.16 (s, 6H), 3.86-3.90 (m, 8H), 1.10-1.26 (m, 48H), 0.80 (t, J = 7.2 Hz, 12H). HRMS (ESI$^+$): calcd for $C_{66}H_{91}N_6O_6$ [M+H]$^+$ 1063.6995, found 1063.6990.

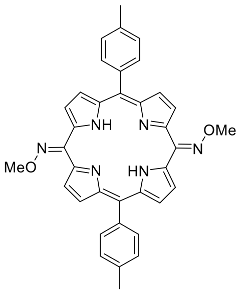

H_2DPP 2d: Following the synthetic procedure step 2. **H_2DPP 1d** (52.1 mg, 0.10 mmol) was used. Purification via column chromatography on silica gel (petroleum ether/ethyl acetate = 8:1, v/v) afforded **H_2DPP 2d** as a red solid (50.1 mg, 87% yield). ^{1}H NMR ($CDCl_3$, 400 MHz): *δ* (ppm) 12.39 (s, 2H), 7.40-7.42 (m, 2H), 7.33-7.35 (m, 4H), 7.24 (s, 2H), 7.13 (d, *J* = 4.4, 2H), 6.81 (d, *J* = 4.8, 2H), 6.35 (m, 2H), 4.20 (s, 6H), 2.46 (s, 6H). HRMS (ESI$^+$): calcd for $C_{36}H_{31}N_6O_2$ $[M+H]^+$ 579.2503, found 579.2505.

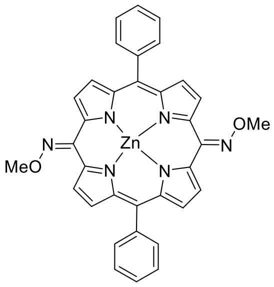

Zn1a: Following the synthetic procedure step 3. *O*-methyl dioxime of 5,15-dioxoporphyrin **Zn1a** was prepared as a black solid (55.1 mg, 98%). ^{1}H NMR ($CDCl_3$, 400 MHz): *δ* (ppm) 7.41-7.48 (m, 10H), 7.31-7.34 (m, 2H), 7.04 (d, *J* = 4.4 Hz, 1H), 6.95 (d, *J* = 4.0 Hz, 1H), 6.68 (t, *J* = 4.0 Hz, 2H), 6.62 (d, *J* = 4.0 Hz, 2H), 4.13 (d, *J* = 6.8 Hz, 6H). HRMS (ESI$^+$): calcd for $C_{34}H_{25}N_6O_2Zn$ $[M+H]^+$ 613.1325, found 613.1323. Single crystals were grown by slow diffusion of *n*-hexane into a solution of **Zn1a** in dichloromethane.

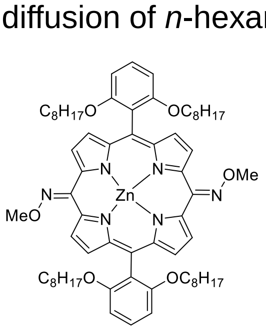

Zn1b: Following the synthetic procedure step 3. *O*-methyl dioxime of 5,15-dioxoporphyrin **Zn1b** was prepared as a black solid (85.2 mg, 97%). The

^{1}H NMR was not recorded because the exist of isomers. HRMS (ESI$^+$): calcd for $C_{66}H_{89}N_6O_6Zn$ [M+H]$^+$ 1125.6130, found 1125.6124.

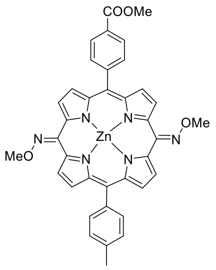

Zn1c: Following the synthetic procedure step 2, **H$_2$DPP 1c** (56.5 mg, 0.10 mmol) was used. Purification via column chromatography on silica gel (petroleum ether/ethyl acetate = 4:1, v/v) afforded **H$_2$DPP 2c** as a red solid (38.1 mg, 61% yield). Then following the procedure step 3, *O*-methyl dioxime of 5,15-dioxoporphyrin **Zn1c** was prepared as a black solid (40.7 mg, 97%). ^{1}H NMR (DMSO-d_6, 400 MHz): δ (ppm) 8.11 (d, J = 8.0 Hz, 2H), 7.53 (d, J = 8.0 Hz, 2H), 7.30-7.35 (m, 4H), 7.24 (d, J = 8.0 Hz, 2H), 6.94-7.05 (m, 2H), 6.72 (d, J = 4.4 Hz, 1H), 6.66 (t, J = 8.4 Hz, 1H), 6.60 (t, J = 9.6 Hz, 1H), 6.53 (d, J = 4.4 Hz, 1H), 4.12-4.14 (m, 6H), 3.98 (s, 3H), 2.46 (s, 3H). HRMS (ESI$^+$): calcd for $C_{37}H_{29}N_6O_4Zn$ [M+H]$^+$ 685.1536, found 685.1529.

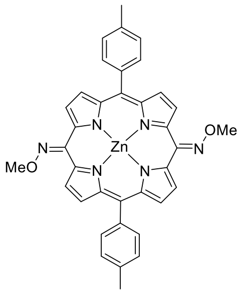

Zn1d: Following the synthetic procedure step 3. *O*-methyl dioxime of 5,15-dioxoporphyrin **Zn1d** was prepared as a black solid (85.2 mg, 97%). ^{1}H NMR (DMSO-d_6, 400 MHz): δ (ppm) 7.26-7.33 (m, 10H), 6.98 (s, 1H), 6.87 (s, 1H), 6.57 (s, 2H), 6.49 (s, 2H), 4.07 (s, 6H), 2.43 (s, 6H). HRMS (ESI$^+$): calcd for $C_{36}H_{29}N_6O_2Zn$ [M+H]$^+$ 641.1638, found 641.1635.

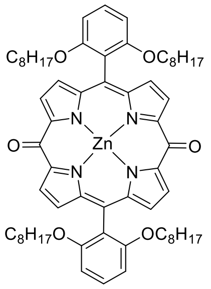

Zn2b: Following the synthetic procedure step 1, 5,15-Bis(2, 6-dioctoxyphenyl)porphyrin (234.1 mg, 0.24 mmol) was used. Purification via column chromatography on silica gel (petroleum ether/ CH_2Cl_2 = 3:1 to 1:1, v/v) afforded **H_2DPP 1b** as a brown solid (115.0 mg, 48% yield). Then following the procedure step 4, 5,15-dioxoporphyrin **Zn2b** was prepared as a black solid (119.8 mg, 98%). ^{1}H NMR ($CDCl_3$, 400 MHz): δ (ppm) 7.26-7.30 (m, 2H), 6.87-6.91 (m, 4H), 6.52-6.56 (m, 4H), 6.22-6.28 (m, 4H), 3.85-3.88 (m, 8H), 1.19-1.25 (m, 48H), 0.80-0.84 (t, J = 6.6 Hz, 12H). HRMS (ESI$^+$): calcd for $C_{64}H_{83}N_4O_6Zn$ $[M+H]^+$ 1067.5599, found 1067.5602.

VII. Preparation and characterization of the doubly pyridine-fused *anti*-quinoidal porphyrins 3 and pyridinium-fused porphyrin cations 4

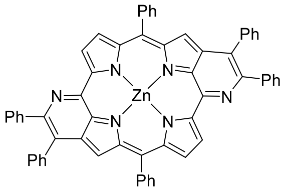

3aa: Following the general procedure. *O*-methyl dioxime of 5,15-dioxoporphyrin **Zn1a** (30.7 mg, 0.05 mmol) and diphenylacetylene (35.6 mg, 0.2 mmol) were used. Purification via column chromatography on neutral alumina (petroleum ether/ethyl acetate = 8:1 to 4:1) afforded **3aa** as a dark green solid (30.1 mg, 66% yield). ^{1}H NMR (DMSO-d_6, 400 MHz): δ (ppm) 7.61 (d, J = 4.8 Hz, 2H), 7.51-7.55 (m, 10H), 7.26-7.32 (m, 10H), 7.17-7.20 (m, 10H), 6.78 (d, J = 4.8 Hz, 2H), 6.16 (s, 2H). HRMS (ESI$^+$): calcd for $C_{60}H_{37}N_6Zn$ $[M+H]^+$ 905.2366; found 905.2360. Single crystals were grown by slow diffusion of *n*-hexane into a solution of **3aa** (in the presence of 1% pyridine) in dichloromethane.

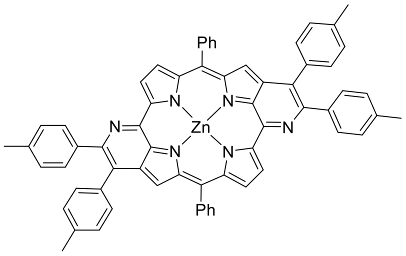

3ab: Following the general procedure. *O*-methyl dioxime of 5,15-dioxoporphyrin **Zn1a** (30.7 mg, 0.05 mmol) and 1,2-di-*p*-tolylethyne (41.3 mg, 0.2 mmol) were used. Purification via column chromatography on neutral alumina (petroleum ether/ethyl acetate = 8:1 to 4:1) afforded **3ab** as a dark green solid (24.1 mg, 50% yield). ^{1}H NMR (DMSO-d_6, 400 MHz): δ (ppm) 7.60 (d, J = 4.8 Hz, 2H), 7.52-7.55 (m, 10H), 7.20 (d, J = 8.0 Hz, 4H), 7.07 (m, 8H), 7.01 (d, J = 8.0 Hz, 4H), 6.77 (d, J = 4.4 Hz, 2H), 6.16 (s, 2H), 2.26 (d, J = 9.6 Hz, 12H). HRMS (ESI$^+$): calcd for $C_{64}H_{45}N_6Zn$ $[M+H]^+$ 961.2992; found 961.2991.

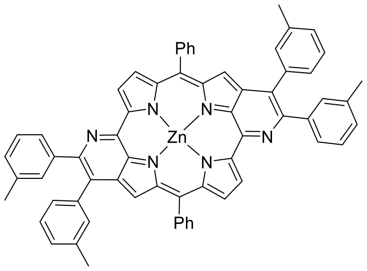

3ac: Following the general procedure. *O*-methyl dioxime of 5,15-dioxoporphyrin **Zn1a** (30.7 mg, 0.05 mmol) and 1,2-di-*m*-tolylethyne (41.3 mg, 0.2 mmol) were used. Purification via column chromatography on neutral alumina (petroleum ether/ethyl acetate = 8:1 to 4:1) afforded **3ac** as a dark green solid (25.4 mg, 53% yield). ^{1}H NMR (DMSO-d_6, 400 MHz): δ (ppm) 7.62 (d, J = 4.8 Hz, 2H), 7.51-7.55 (m, 10H), 7.14-7.22 (m, 4H), 6.99-7.08 (m, 10H), 6.95 (d, J = 7.6 Hz, 2H), 6.80 (d, J = 4.8 Hz, 2H), 6.15 (s, 2H), 2.20 (s, 6H), 2.17 (s, 6H). HRMS (ESI$^+$): calcd for $C_{64}H_{45}N_6Zn$ $[M+H]^+$ 961.2992; found 961.2991.

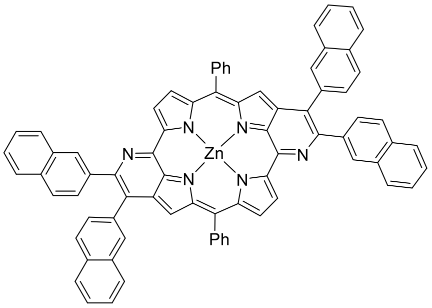

3ad: Following the general procedure. *O*-methyl dioxime of 5,15-dioxoporphyrin **Zn1a** (30.7 mg, 0.05 mmol) and 1, 2-di(naphthalene-2-yl)ethyne (55.7 mg, 0.2 mmol) were used. Purification via column chromatography on neutral alumina (petroleum ether/ethyl acetate = 8:1 to 4:1) afforded **3ad** as a dark green solid (30.0 mg, 54% yield). ^{1}H NMR (DMSO-d_6, 400 MHz): δ (ppm) 7.97 (s, 2H), 7.85 (d, J = 8.8 Hz, 4H), 7.75-7.78 (m, 6H), 7.69-7.71 (m, 4H), 7.65 (s, 1H), 7.63 (s, 1H), 7.55-7.57 (m, 4H), 7.46-7.51 (m, 10H), 7.38-7.42 (m, 6H), 7.30 (d, J = 8.4 Hz, 2H), 6.83 (d, J = 4.8 Hz, 2H), 6.25 (s, 2H). HRMS (ESI$^+$): calcd for $C_{76}H_{45}N_6Zn$ [M+H]$^+$ 1105.2992; found 1105.2992.

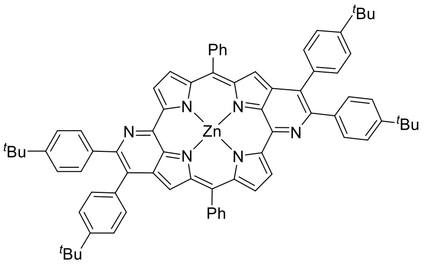

3ae: Following the general procedure. *O*-methyl dioxime of 5,15-dioxoporphyrin **Zn1a** (30.7 mg, 0.05 mmol) and 1,2-bis(4-*tert*-butylphenyl)ethyne (58.1 mg, 0.2 mmol) were used. Purification via column chromatography on neutral alumina (petroleum ether/ethyl acetate = 8:1 to 4:1) afforded **3ae** as a dark green solid (33.9 mg, 60% yield). ^{1}H NMR ($CDCl_3$, 400 MHz): δ (ppm) 7.70 (d, J = 4.8 Hz, 2H), 7.37-7.47 (m, 10H), 7.24 (d, J = 8.4 Hz, 4H), 7.19 (d, J = 8.4 Hz, 4H), 7.06-7.13 (m, 8H), 6.74 (d, J = 4.4 Hz, 2H), 6.40 (s, 2H), 1.29 (s, 18H), 1.24 (s, 18H). HRMS (ESI$^+$): calcd for $C_{76}H_{69}N_6Zn$ [M+H]$^+$ 1129.4870; found 1129.4872.

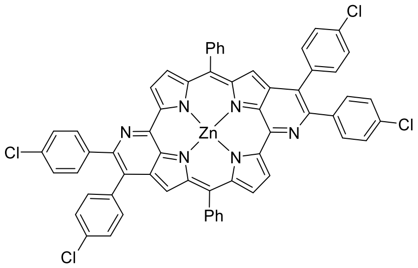

3af: Following the general procedure. *O*-methyl dioxime of 5,15-dioxoporphyrin **Zn1a** (30.7 mg, 0.05 mmol) and 1, 2-bis(4-chlorophenyl)ethyne (49.4 mg, 0.2 mmol) were used. Purification via column chromatography on neutral alumina (petroleum ether/ethyl acetate = 8:1 to 4:1) afforded **3af** as a dark green solid (26.5 mg, 51% yield). ^{1}H NMR (DMSO-d_6, 400 MHz): δ (ppm) 7.65 (d, J = 4.8 Hz, 2H), 7.42-7.46 (m, 10H), 7.27 (d, J = 7.2 Hz, 4H), 7.12-7.21 (m, 16H), 6.78 (d, J = 4.8 Hz, 2H), 6.28 (s, 2H). HRMS (ESI$^+$): calcd for $C_{60}H_{33}Cl_4N_6Zn$ [M+H]$^+$ 1041.0807 (^{35}Cl), 1043.0777 (^{37}Cl); found 1041.0811, 1043.0771.

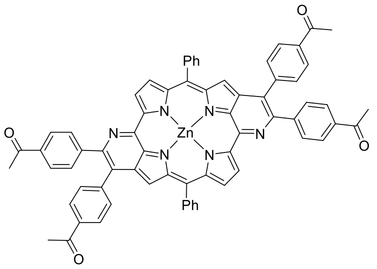

3ag: Following the general procedure. **Zn1a** (30.7mg, 0.05mmol) and 1,1'-(4,4'-(ethyne-1,2-diyl)bis(4,1-phenylene))diethanone (52.5 mg, 0.2 mmol) were used. Purification via column chromatography on neutral alumina (petroleum ether/ethyl acetate = 1:1 to 1:3) afforded **3ag** as a dark green solid (33.3 mg, 62% yield). The solubility of the product proved too low in common organic solvents to allow the ^{1}H NMR spectrum to be recorded. HRMS (ESI$^+$): calcd for $C_{68}H_{45}O_4N_6Zn$ [M+H]$^+$ 1073.2788; found 1073.2800.

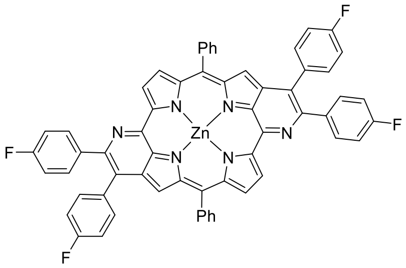

3ah: Following the general procedure. *O*-methyl dioxime of 5,15-dioxoporphyrin **Zn1a** (30.7 mg, 0.05 mmol) and 1, 2-bis(4-fluorophenyl)ethyne (42.8 mg, 0.2 mmol) were used. Purification via column chromatography on neutral alumina (petroleum ether/ethyl acetate = 8:1 to 4:1) afforded **3ah** as a dark green solid (19.5 mg, 40% yield). ^{1}H NMR (DMSO-d_6, 400 MHz): δ (ppm) 7.59 (d, J = 4.8 Hz, 2H), 7.51-7.54 (m, 10H), 7.30-7.34 (m, 4H), 7.20-7.23 (m, 4H), 7.12-7.17 (m, 4H), 7.04-7.08 (m, 4H), 6.77 (d, J = 4.8 Hz, 2H), 6.13 (s, 2H). HRMS (ESI$^+$): calcd for $C_{60}H_{33}F_4N_6Zn$ $[M+H]^+$ 977.1989; found 977.1993.

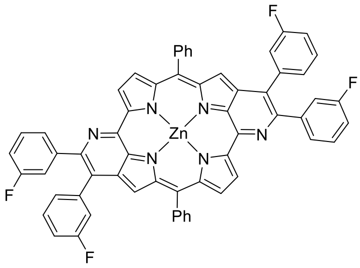

3ai: Following the general procedure. *O*-methyl dioxime of 5,15-dioxoporphyrin **Zn1a** (30.7 mg, 0.05 mmol) and 1, 2-bis(3-fluorophenyl)ethyne (42.8 mg, 0.2 mmol) were used. Purification via column chromatography on neutral alumina (petroleum ether/ethyl acetate = 8:1 to 4:1) afforded **3ai** as a dark green solid (26.9 mg, 55% yield). ^{1}H NMR (DMSO-d_6, 400 MHz): δ (ppm) 7.52-7.60 (m, 12H), 7.32-7.38 (m, 2H), 7.22-7.28 (m, 2H), 7.10-7.16 (m, 6H), 7.01-7.07 (m, 6H), 6.75-6.78 (m, 2H), 6.13 (s, 2H). HRMS (ESI$^+$): calcd for $C_{60}H_{33}F_4N_6Zn$ $[M+H]^+$ 977.1989; found 977.1994.

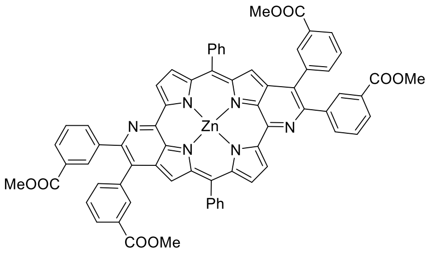

3aj: Following the general procedure. *O*-methyl dioxime of 5,15-dioxoporphyrin **Zn1a** (30.7 mg, 0.05 mmol) and 1, 2-bis(3-(methoxycarbonyl)phenyl)ethyne (58.9 mg, 0.2 mmol) were used. Purification via column chromatography on

neutral alumina (petroleum ether/ethyl acetate = 3:1 to 1:1) afforded **3aj** as a dark green solid (29.1 mg, 51% yield). ^{1}H NMR (DMSO-d_6, 400 MHz): δ (ppm) 7.97 (t, J = 3.6 Hz, 2H), 7.84-7.87 (m, 2H), 7.78-7.80 (m, 4H), 7.60 (d, J = 4.4 Hz, 2H), 7.50-7.54 (m, 12H), 7.43-7.45 (m, 4H), 7.33 (t, J = 7.8 Hz, 2H), 6.81 (d, J = 4.8 Hz, 2H), 6.13 (s, 2H), 3.80 (s, 6H), 3.78 (s, 6H). HRMS (ESI$^+$): calcd for $C_{68}H_{45}O_8N_6Zn$ $[M+H]^+$ 1137.2585; found 1137.2578.

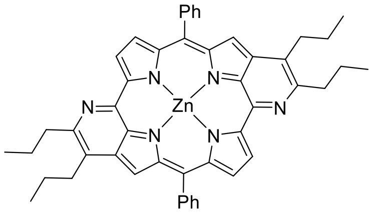

3ak: Following the general procedure. *O*-methyl dioxime of 5,15-dioxoporphyrin **Zn1a** (30.7 mg, 0.05 mmol) and 4-octyne (22.1 mg, 0.2 mmol) were used. Purification via column chromatography on neutral alumina (petroleum ether/ethyl acetate = 8:1 to 4:1) afforded **3ak** as a dark green solid (16.2 mg, 42% yield). ^{1}H NMR (DMSO-d_6, 400 MHz): δ (ppm) 7.55-7.59 (m, 12H), 6.74 (d, J = 4.8 Hz, 2H), 6.30 (s, 2H), 2.66-2.74 (m, 8H), 1.72-1.77 (m, 4H), 1.50-1.55 (m, 4H), 0.96 (t, J = 7.4 Hz, 6H), 0.87 (t, J = 7.4 Hz, 6H). HRMS (ESI$^+$): calcd for $C_{48}H_{45}N_6Zn$ $[M+H]^+$ 769.2992; found 769.2994.

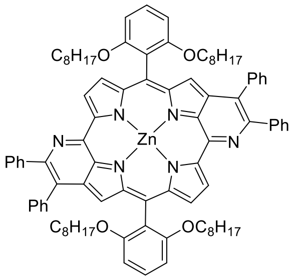

3ba: Following the general procedure. *O*-methyl dioxime of 5,15-dioxoporphyrin **Zn1b** (56.3 mg, 0.05 mmol) and diphenylacetylene (35.6 mg, 0.2 mmol) were used. Purification via column chromatography on neutral alumina (petroleum ether/ethyl acetate = 20:1 to 10:1) afforded **3ba** as a dark green solid (28.3 mg, 40% yield). ^{1}H NMR ($CDCl_3$, 400 MHz): δ (ppm) 7.70 (d, J = 4.8 Hz, 2H), 7.37-7.39 (m, 4H), 7.30 (t, J = 8.2 Hz, 2H), 7.20-7.22 (m, 10H), 7.16-7.19 (m, 6H), 6.82 (d, J = 4.8 Hz, 2H), 6.61 (d, J = 8.4 Hz, 4H), 6.41 (s,

2H), 3.86-3.96 (m, 8H), 1.25-1.26 (m, 8H), 1.08-1.12 (m, 40H), 0.72 (t, *J* = 6.8 Hz, 12H). HRMS (ESI$^+$): calcd for $C_{92}H_{101}O_4N_6Zn$ $[M+H]^+$ 1439.6990; found 1439.6998.

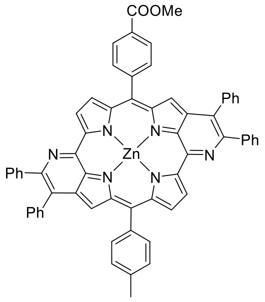

3ca: Following the general procedure. *O*-methyl dioxime of 5,15-dioxoporphyrin **Zn1c** (34.3 mg, 0.05 mmol) and diphenylacetylene (35.6 mg, 0.2 mmol) were used. Purification via column chromatography on neutral alumina (petroleum ether/ethyl acetate = 8:1 to 4:1) afforded **3ca** as a dark green solid (14.7 mg, 30% yield). ^{1}H NMR (DMSO-d_6, 400 MHz): δ (ppm) 8.08 (d, *J* = 8.4 Hz, 2H), 7.77 (t, *J* = 4.6 Hz, 2H), 7.54 (t, *J* = 7.4 Hz, 2H), 7.34-7.38 (m, 6H), 7.17-7.24 (m, 18H), 6.87 (d, *J* = 4.8 Hz, 1H), 6.73 (d, *J* = 4.8 Hz, 1H), 6.48 (s, 1H), 6.32 (s, 1H), 3.99 (s, 3H), 2.46 (s, 3H). HRMS (ESI$^+$): calcd for $C_{63}H_{41}O_2N_6Zn$ $[M+H]^+$ 977.2577; found 977.2586.

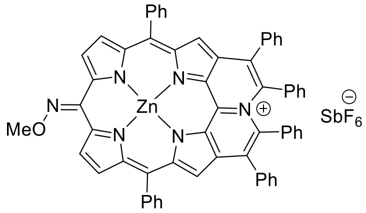

4aa: Following the general procedure. *O*-methyl dioxime of 5,15-dioxoporphyrin **Zn1a** (30.7 mg, 0.05 mmol) and diphenylacetylene (35.6 mg, 0.2 mmol) were used. Purification via column chromatography on silica gel (petroleum ether/ethyl acetate = 1:1 to 1:3) afforded **4aa** as a black solid (32.2 mg, 55% yield). ^{1}H NMR (DMSO-d_6, 400 MHz): δ (ppm) 7.82 (s, 2H), 7.59-7.66 (m, 10H), 7.10-7.18 (m, 8H), 6.99 (s, 4H), 6.86-6.89 (m, 2H), 6.75-6.79 (m, 4H), 6.39-6.48 (m, 6H), 4.45 (s, 3H). HRMS (ESI$^+$): calcd for $C_{61}H_{39}ON_6Zn$ $[M]^+$ 935.2471; found 935.2473.

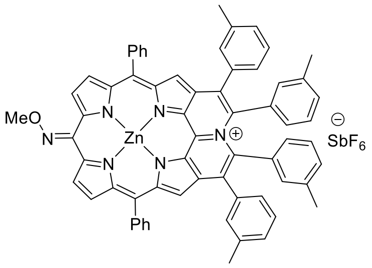

4ac: Following the general procedure. *O*-methyl dioxime of 5,15-dioxoporphyrin **Zn1a** (30.7 mg, 0.05 mmol) and 1,2-di-*m*-tolylethyne (41.3 mg, 0.2 mmol) were used. Purification via column chromatography on silica gel (petroleum ether/ethyl acetate = 1:1 to 1:3) afforded **4ac** as a black solid (24.5 mg, 40% yield). ^{1}H NMR (DMSO-d_6, 400 MHz): δ (ppm) 7.81 (d, J = 4.8 Hz, 1H), 7.65 (d, J = 4.8 Hz, 1H), 7.58-7.61 (m, 10H), 6.40-7.19 (m, 20H), 4.44 (s, 3H), 1.72-2.19 (m, 12H). HRMS (ESI$^+$): calcd for $C_{65}H_{47}ON_6Zn$ $[M]^+$ 991.3097; found 991.3098.

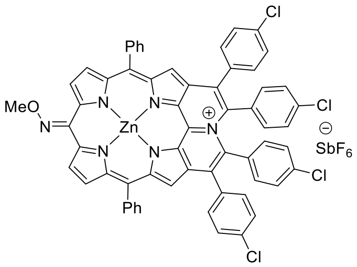

4af: Following the general procedure. *O*-methyl dioxime of 5,15-dioxoporphyrin **Zn1a** (30.7 mg, 0.05 mmol) and 1, 2-bis(4-chlorophenyl)ethyne (49.4 mg, 0.2 mmol) were used. Purification via column chromatography on silica gel (petroleum ether/ethyl acetate = 1:1 to 1:3) afforded **4af** as a black solid (32.8 mg, 50% yield). ^{1}H NMR (DMSO-d_6, 400 MHz): δ (ppm) 7.81 (d, J = 4.8 Hz, 1H), 7.66 (d, J = 5.2 Hz, 1H), 7.58-7.63 (m, 10H), 7.30 (d, J = 0.8 Hz, 2H), 7.28 (d, J = 1.2 Hz, 2H), 7.12 (d, J = 4.8 Hz, 1H), 7.09 (d, J = 5.2 Hz, 1H), 7.03-7.06 (m, 4H), 6.91-6.94 (m, 4H), 6.54-6.59 (t, J = 8.4 Hz, 4H), 6.46 (s, 1H), 6.38 (s, 1H), 4.45 (s, 3H). HRMS (ESI$^+$): calcd for $C_{61}H_{35}Cl_4ON_6Zn$ $[M]^+$ 1071.0912 (^{35}Cl), 1073.0883 (^{37}Cl); found 1071.0912, 1073.0877.

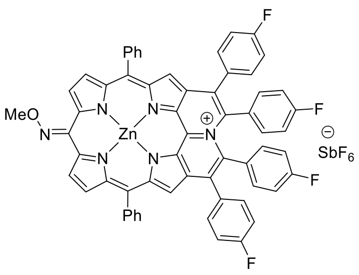

4ah: Following the general procedure. *O*-methyl dioxime of 5,15-dioxoporphyrin **Zn1a** (30.7 mg, 0.05 mmol) and 1, 2-bis(4-fluorophenyl)ethyne (42.8 mg, 0.2 mmol) were used. Purification via column chromatography on silica gel (petroleum ether/ethyl acetate = 1:1 to 1:3) afforded **4ah** as a black solid (18.6 mg, 30% yield). ^{1}H NMR (DMSO-d_6, 400 MHz): δ (ppm) 7.87 (d, J = 4.8 Hz, 1H), 7.70 (d, J = 4.8 Hz, 1H), 7.56-7.64 (m, 10H), 7.16 (d, J = 4.8 Hz, 1H), 7.13 (d, J = 4.8 Hz, 1H), 7.03-7.07 (m, 8H), 6.70-6.75 (m, 4H), 6.55-6.60 (m, 4H), 6.50 (s, 1H), 6.42 (s, 1H), 6.47 (s, 3H). HRMS (ESI$^+$): calcd for $C_{61}H_{35}F_4ON_6Zn$ [M]$^+$ 1007.2094; found 1007.2099. Single crystals were grown by slow diffusion of *n*-hexane into a solution of **4ah** (in the presence of 1% tetrahydrofuran) in dichloromethane.

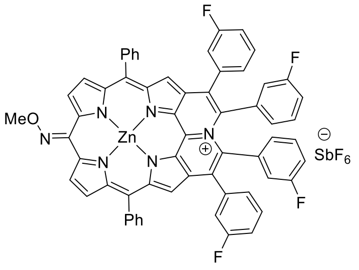

4ai: Following the general procedure. *O*-methyl dioxime of 5,15-dioxoporphyrin **Zn1a** (30.7 mg, 0.05 mmol) and 1, 2-bis(4-fluorophenyl)ethyne (42.8 mg, 0.2 mmol) were used. Purification via column chromatography on silica gel (petroleum ether/ethyl acetate = 1:1 to 1:3) afforded **4ai** as a black solid (31.1 mg, 50% yield). ^{1}H NMR (DMSO-d_6, 400 MHz): δ (ppm) 7.85 (d, J = 4.8 Hz, 1H), 7.69 (d, J = 4.8 Hz, 1H), 7.59-7.64 (m, 10H), 6.66-7.35 (m, 18H), 6.50 (s, 1H), 6.42 (s, 1H), 4.46 (s, 3H). HRMS (ESI$^+$): calcd for $C_{61}H_{35}F_4ON_6Zn$ [M]$^+$ 1007.2094; found 1007.2088.

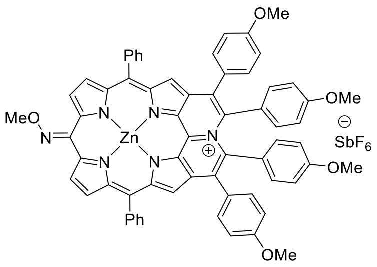

4al: Following the general procedure. *O*-methyl dioxime of 5,15-dioxoporphyrin **Zn1a** (30.7 mg, 0.05 mmol) and 1, 2-bis(4-methoxyphenyl)ethyne (47.7 mg, 0.2 mmol) were used. Purification via column chromatography on silica gel (petroleum ether/ethyl acetate = 1:1 to 1:3) afforded **4al** as a black solid (39.1 mg, 60% yield). ^{1}H NMR (DMSO-d_6, 400 MHz): δ (ppm) 7.85 (d, J = 4.4 Hz, 1H), 7.68 (d, J = 4.8 Hz, 1H), 7.57-7.62 (m, 10H), 7.13 (d, J = 4.8 Hz, 1H), 7.10 (d, J = 4.4 Hz, 1H), 6.88-6.91 (m, 4H), 6.76 (d, J = 8.0 Hz, 4H), 6.52 (s, 1H), 6.44 (s, 1H), 6.35-6.41 (m, 8H), 4.45 (s, 3H), 3.67 (s, 6H), 3.61 (d, J = 2.8 Hz, 6H). HRMS (ESI$^+$): calcd for $C_{65}H_{47}O_5N_6Zn$ $[M]^+$ 1055.2894; found 1055.2899.

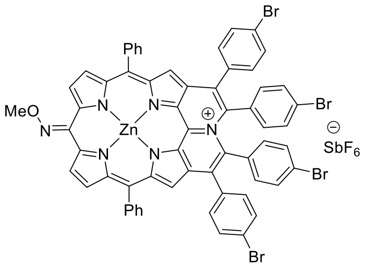

4am: Following the general procedure. *O*-methyl dioxime of 5,15-dioxoporphyrin **Zn1a** (30.7 mg, 0.05 mmol) and 1, 2-bis(4-bromophenyl)ethyne (67.2 mg, 0.2 mmol) were used. Purification via column chromatography on silica gel (petroleum ether/ethyl acetate = 1:1 to 1:3) afforded **4am** as a black solid (48.4 mg, 65% yield). ^{1}H NMR (DMSO-d_6, 400 MHz): δ (ppm) 7.82 (d, J = 4.8 Hz, 1H), 7.66 (d, J = 4.8 Hz, 1H), 7.58-7.63 (m, 10H), 7.43 (d, J = 7.6 Hz, 4H), 7.12 (d, J = 4.8 Hz, 1H), 7.10 (d, J = 4.4 Hz, 1H), 7.04-7.07 (m, 4H), 6.97-7.00 (m, 4H), 6.49 (t, J = 8.4 Hz, 4H), 6.46 (s, 1H), 6.38 (s, 1H), 4.45 (s, 3H). HRMS (ESI$^+$): calcd for $C_{61}H_{35}Br_4ON_6Zn$ $[M]^+$ 1246.8892 (^{79}Br), 1248.8871 (^{81}Br); found 1246.8886, 1248.8863.

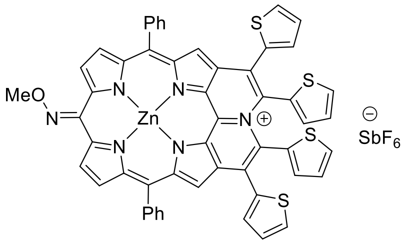

4an: Following the general procedure. *O*-methyl dioxime of 5,15-dioxoporphyrin **Zn1a** (30.7 mg, 0.05 mmol) and 1,2-di(thiophen-2-yl)ethyne (38.1 mg, 0.2 mmol) were used. Purification via column chromatography on silica gel (petroleum ether/ethyl acetate = 1:1 to 1:2) afforded **4an** as a black solid (20.9 mg, 35% yield). ^{1}H NMR (DMSO-d_6, 400 MHz): δ (ppm) 7.83 (d, J = 4.8 Hz, 1H), 7.68 (d, J = 4.8 Hz, 1H), 7.61-7.64 (m, 10H), 7.54-7.57 (m, 2H), 7.45-7.49 (m, 2H), 7.16 (d, J = 4.8 Hz, 1H), 7.13 (d, J = 5.2 Hz, 1H), 6.99-7.04 (m, 4H), 6.65-6.69 (m, 3H), 6.60 (s, 1H), 6.34-6.37 (m, 2H), 4.46 (s, 3H). HRMS (ESI$^+$): calcd for $C_{53}H_{31}S_4ON_6Zn$ [M]$^+$ 959.0728; found 959.0729.

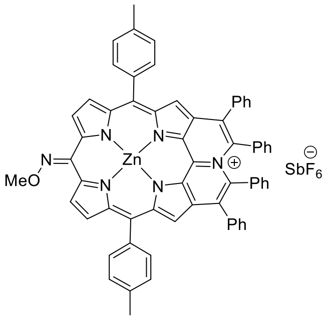

4da: Following the general procedure. *O*-methyl dioxime of 5,15-dioxoporphyrin **Zn1d** (30.7 mg, 0.05 mmol) and diphenylacetylene (35.6 mg, 0.2 mmol) were used. Purification via column chromatography on silica gel (petroleum ether/ethyl acetate = 1:1 to 1:3) afforded **4da** as a black solid (18.0 mg, 30% yield). ^{1}H NMR (DMSO-d_6, 400 MHz): δ (ppm) 7.81 (d, J = 4.8 Hz, 1H), 7.65 (d, J = 5.2 Hz, 1H), 7.48 (d, J = 7.6 Hz, 4H), 7.38 (d, J = 8.0 Hz, 4H), 7.11-7.19 (m, 10H), 6.97-7.00 (m, 4H), 6.85-6.88 (m, 2H), 6.74-6.79 (m, 4H), 6.52 (s, 1H), 6.43-6.47 (m, 3H), 4.45 (s, 3H), 2.44 (d, J = 2.4 Hz, 6H). HRMS (ESI$^+$): calcd for $C_{63}H_{43}ON_6Zn$ [M]$^+$ 963.2784; found 963.2781.

VIII. Preparation and characterization of the doubly pyran-fused porphyrins 5 and pyrylium-fused porphyrin dimer 6

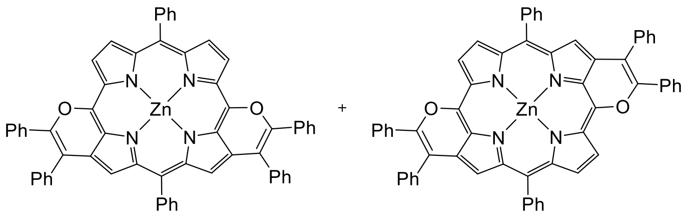

5aa: A schlenk tube with a magnetic stir bar was charged with $[Cp^{*}RhCl_2]_2$ (1.5 mg, 2.5 μmol), $AgSbF_6$ (3.4 mg, 10 μmol), Ag_2O (23.2 mg, 0.1 mmol), 5,15-dioxoporphyrin **Zn2a** (27.8 mg, 0.05 mmol), diphenylacetylene (35.6 mg, 0.2 mmol), and dioxane (0.5 mL) under N_2 atmosphere. The resulting solution was stirred at room temperature for 10 min and then at 120 ^oC for 24 h. After being cooled down, the reaction mixture was filtered through a Celite pad, and then washed with 30 mL of CH_2Cl_2. The solvent of the filtrate was removed under reduced pressure and the residue was absorbed to small amounts of silica gel. Purification via column chromatography on neutral Al_2O_3 (petroleum ether/ethyl acetate = 6:1, v/v) afforded **5aa** as a dark green solid of a mixture of *syn*- and *anti*-configurations in a ratio of ca. 2:1 (27.2 mg, 60% yield). HRMS (ESI^+): calcd for $C_{60}H_{37}N_4O_2Zn$ $[M+H]^+$ 909.2202; found 909.2198. Slow diffusion of *n*-hexane into a solution of **5aa** in dichloromethane (in the presence of 1% tetrahydrofuran) gave the single crystals of *syn*-configuration.

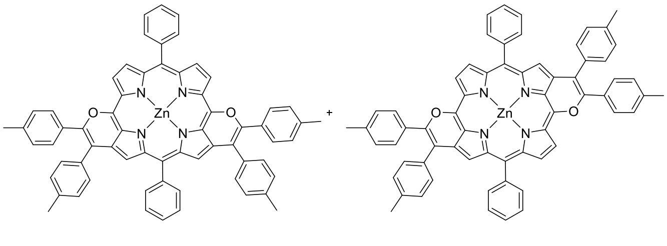

5ab: A schlenk tube with a magnetic stir bar was charged with $[Cp^{*}RhCl_2]_2$ (1.5 mg, 2.5 μmol), $AgSbF_6$ (3.4 mg, 10 μmol), Ag_2O (23.2 mg, 0.1 mmol), 5,15-dioxoporphyrin **Zn2a** (27.8 mg, 0.05 mmol), 1,2-di-*p*-tolylethyne (41.3 mg, 0.2 mmol), and DCE (0.5 mL) under N_2 atmosphere. The resulting solution was stirred at room temperature for 10 min and then at 120 ^oC for 24 h. After being

cooled down, the reaction mixture was filtered through a Celite pad, and then washed with 30 mL of CH_2Cl_2. The solvent of the filtrate was removed under reduced pressure and the residue was absorbed to small amounts of silica gel. Purification via column chromatography on neutral Al_2O_3 (petroleum ether/ethyl acetate = 6:1, v/v) afforded **5ab** as a dark green solid of a mixture of *syn*- and *anti*-configurations in a ratio of 3.8:1 (21.7 mg, 45% yield). HRMS (ESI^+): calcd for $C_{64}H_{45}N_4O_2Zn$ $[M+H]^+$ 965.2828; found 965.2831.

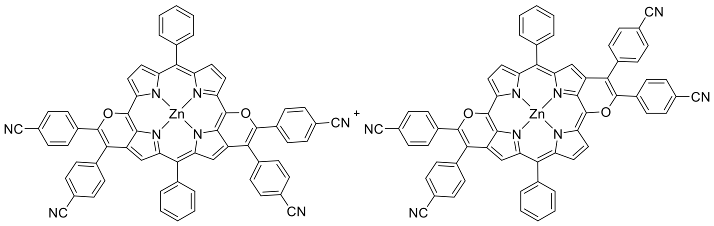

5ao: A schlenk tube with a magnetic stir bar was charged with $[Cp^*RhCl_2]_2$ (1.5 mg, 2.5 μmol), $AgSbF_6$ (3.4 mg, 10 μmol), Ag_2O (23.2 mg, 0.1 mmol), 5,15-dioxoporphyrin **Zn2a** (27.8 mg, 0.05 mmol), 4,4'-(Ethyne-1,2-diyl)dibenzonitrile (45.7 mg, 0.2 mmol), and DCE (0.5 mL) under N_2 atmosphere. The resulting solution was stirred at room temperature for 10 min and then at 120 ^oC for 24 h. After being cooled down, the reaction mixture was filtered through a Celite pad, and then washed with 30 mL of CH_2Cl_2. The solvent of the filtrate was removed under reduced pressure and the residue was absorbed to small amounts of silica gel. Purification via column chromatography on neutral Al_2O_3 (petroleum ether/ethyl acetate = 6:1 to 1:1 v/v) afforded **5ao** as a dark green solid of a mixture of *syn*- and *anti*-configurations in a ratio of 4:1 (29.8 mg, 59% yield). HRMS (ESI^+): calcd for $C_{64}H_{33}N_8O_2Zn$ $[M+H]^+$ 1009.2012; found 1009.2021.

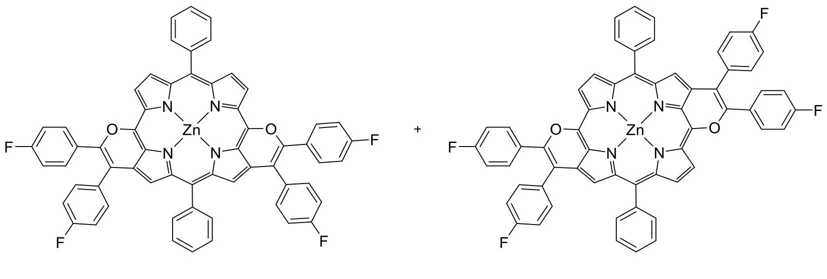

5ah: A schlenk tube with a magnetic stir bar was charged with $[Cp^*RhCl_2]_2$ (1.5

mg, 2.5 μmol), $AgSbF_6$ (3.4 mg, 10 μmol), Ag_2O (23.2 mg, 0.1 mmol), 5,15-dioxoporphyrin **Zn2b** (53.4 mg, 0.05 mmol), 1, 2-bis(4-fluorophenyl)ethyne (42.8 mg, 0.2 mmol), and DCE (0.5 mL) under N_2 atmosphere. The resulting solution was stirred at room temperature for 10 min and then at 120 ^oC for 24 h. After being cooled down, the reaction mixture was filtered through a Celite pad, and then washed with 30 mL of CH_2Cl_2. The solvent of the filtrate was removed under reduced pressure and the residue was absorbed to small amounts of silica gel. Purification via column chromatography on neutral Al_2O_3 (petroleum ether/ethyl acetate = 4:1, v/v) afforded **5ah** as a dark green solid of a mixture of *syn*- and *anti*-configurations in a ratio of ca. 1:1 (30.1 mg, 58% yield). HRMS (ESI$^+$): calcd for $C_{60}H_{33}F_4N_4O_2Zn$ $[M+H]^+$ 981.1826; found 981.1819.

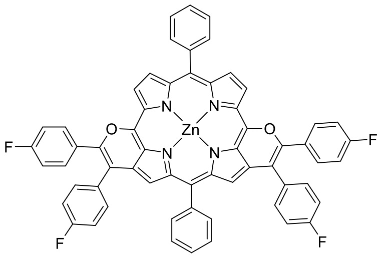

syn-**5ah**: HPLC chromatograph was performed with the mixture of **5ah** to afford single *syn* isomer using CH_2Cl_2 (0.1% DEA)/*n*-Hexane as eluent. ^1H NMR ($CDCl_3$, 400 MHz): *δ* (ppm) 9.56 (d, *J* = 4.4 Hz, 2H), 8.91 (d, *J* = 4.4 Hz, 2H), 8.38 (s, 2H), 8.25 (d, *J* = 7.2 Hz, 2H), 8.15 (d, *J* = 6.8 Hz, 2H), 7.87-7.91 (m, 4H), 7.80 (d, *J* = 6.4 Hz, 2H), 7.70-7.76 (m, 6H), 7.18-7.24 (m, 10H). HRMS (ESI$^+$): calcd for $C_{60}H_{33}F_4N_4O_2Zn$ $[M+H]^+$ 981.1826; found 981.1821. Slow diffusion of *n*-hexane into a solution of *syn*-**5ah** in dichloromethane (in the presence of 1% tetrahydrofuran) gave the single crystals for X-ray diffraction analysis.

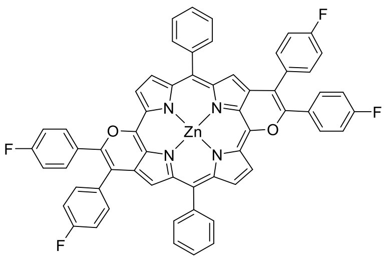

anti-**5ah**: HPLC chromatograph was performed with the mixture of **5ah** to afford single *anti* isomer using CH_2Cl_2 (0.1% DEA)/*n*-Hexane as eluent. ^{1}H NMR ($CDCl_3$, 400 MHz): δ (ppm) 9.68 (d, J = 4.4 Hz, 2H), 8.93 (d, J = 4.4 Hz, 2H), 8.53 (s, 2H), 8.22-8.24 (m, 4H), 7.91-7.95 (m, 4H), 7.76-7.81 (m, 10H), 7.19-7.28 (m, 8H). HRMS (ESI$^+$): calcd for $C_{60}H_{33}F_4N_4O_2Zn$ $[M+H]^+$ 981.1826; found 981.1823. Slow diffusion of *n*-hexane into a solution of *anti*-**5ah** in dichloromethane (in the presence of 1% tetrahydrofuran) gave the single crystals for X-ray diffraction analysis.

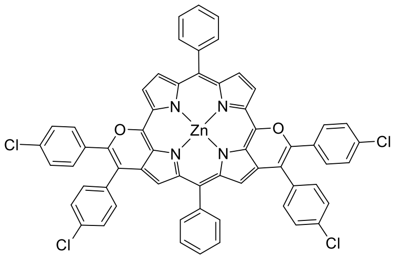

syn-**5af**: A schlenk tube with a magnetic stir bar was charged with $[Cp^*RhCl_2]_2$ (1.5 mg, 2.5 µmol), $AgSbF_6$ (3.4 mg, 10 µmol), Ag_2O (23.2 mg, 0.1 mmol), 5,15-dioxoporphyrin **Zn2a** (27.8 mg, 0.05 mmol), 1,2-bis(4-chlorophenyl)ethyne (49.4 mg, 0.2 mmol), and DCE (0.5 mL) under N_2 atmosphere. The resulting solution was stirred at room temperature for 10 min and then at 120 ^oC for 24 h. After being cooled down, the reaction mixture was filtered through a Celite pad, and then washed with 30 mL of CH_2Cl_2. The solvent of the filtrate was removed under reduced pressure and the residue was absorbed to small amounts of silica gel. Purification via column chromatography on neutral Al_2O_3 (petroleum ether/ethyl acetate = 6:1 to 2:1, v/v) afforded crude product of a mixture of *syn*- and *anti*-configurations in a ratio of above 10:1, which was further purified by re-crystallization from

petroleum ether/ ethyl acetate to give pure *syn*-**5af** (25.1 mg, 48% yield) as a dark green solid . ^{1}H NMR ($CDCl_3$, 400 MHz): δ (ppm) 9.58 (d, J = 4.4 Hz, 2H), 8.94 (d, J = 4.4 Hz, 2H), 8.41 (s, 2H), 8.27 (d, J = 5.2 Hz, 2H), 8.14 (d, J = 6.8 Hz, 2H), 7.80-7.85 (m, 6H), 7.71-7.74 (m, 6H), 7.48-7.54 (m, 10H). HRMS (ESI$^+$): calcd for $C_{60}H_{32}Cl_4N_4NaO_2Zn$ [M+Na]$^+$ 1067.0463 (^{35}Cl), 1069.0434 (^{37}Cl); found 1067.0462, 1069.0425. Slow diffusion of *n*-hexane into a solution of *syn*-**5af** in dichloromethane (in the presence of 1% pyridine) gave the single crystals for X-ray diffraction analysis.

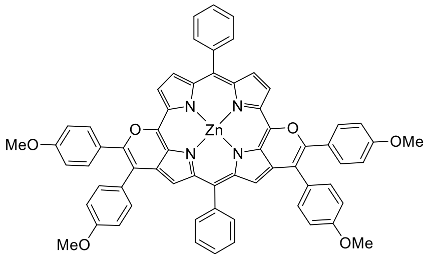

syn-**5al**: A schlenk tube with a magnetic stir bar was charged with $[Cp^*RhCl_2]_2$ (1.5 mg, 2.5 µmol), $AgSbF_6$ (3.4 mg, 10 µmol), Ag_2O (23.2 mg, 0.1 mmol), 5,15-dioxoporphyrin **Zn2a** (27.8 mg, 0.05 mmol), 1,2-bis(4-methoxyphenyl)ethyne (47.7 mg, 0.2 mmol), and DCE (0.5 mL) under N_2 atmosphere. The resulting solution was stirred at room temperature for 10 min and then at 120 ^{o}C for 24 h. After being cooled down, the reaction mixture was filtered through a Celite pad, and then washed with 30 mL of CH_2Cl_2. The solvent of the filtrate was removed under reduced pressure and the residue was absorbed to small amounts of silica gel. Purification via column chromatography on neutral Al_2O_3 (petroleum ether/ethyl acetate = 1:1 to 1:3, v/v) afforded crude product of a mixture of *syn*- and *anti*-configurations in a ratio of ca. 9:1, which was further purified by re-crystallization from petroleum ether/CH_2Cl_2 to give pure *syn*-**5al** (18.3 mg, 35% yield) as a dark green solid . ^{1}H NMR ($CDCl_3$, 400 MHz): δ (ppm) 9.58 (d, J = 4.0 Hz, 2H), 8.89 (d, J = 4.4 Hz, 2H), 8.39 (s, 2H), 8.27 (d, J = 6.8 Hz, 2H), 8.16 (d, J = 6.4 Hz, 2H), 7.86 (d, J = 8.0 Hz, 4H), 7.80 (d, J = 5.2 Hz, 4H), 7.70-7.72 (m, 6H), 7.07 (d, J = 4.0 Hz, 4H), 7.01 (d, J = 8.8 Hz, 4H), 3.94 (s, 6H), 3.91 (s, 6H). HRMS

(ESI$^+$): calcd for $C_{64}H_{45}N_4O_6Zn$ $[M+H]^+$ 1029.2625; found 1029.2624.

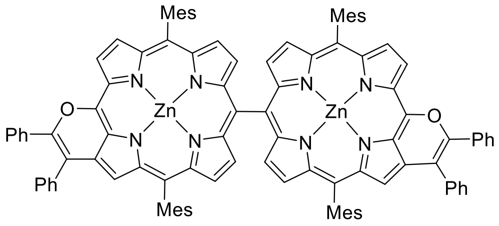

5ea: A schlenk tube with a magnetic stir bar was charged with $[Cp^*RhCl_2]_2$ (1.5 mg, 2.5 μmol), $AgSbF_6$ (3.4 mg, 10 μmol), Ag_2O (23.2 mg, 0.1 mmol), 5,15-dioxoporphyrin dimer **Zn2e** (62.4 mg, 0.05 mmol), diphenylacetylene (35.6 mg, 0.2 mmol), and DCE (0.5 mL) under N_2 atmosphere. The resulting solution was stirred at room temperature for 10 min and then at 120 ^oC for 24 h. After being cooled down, the reaction mixture was filtered through a Celite pad, and then washed with 30 mL of CH_2Cl_2. The solvent of the filtrate was removed under reduced pressure and the residue was absorbed to small amounts of silica gel. Purification via column chromatography on neutral Al_2O_3 (petroleum ether/ethyl acetate = 6:1, v/v) afforded **5ea** as a dark green solid (40.1 mg, 50% yield). ^1H NMR ($CDCl_3$, 400 MHz): δ (ppm) 9.70 (d, J = 4.4 Hz, 2H), 8.75 (d, J = 4.4 Hz, 2H), 8.39 (s, 2H), 8.38 (d, J = 4.4 Hz, 2H), 8.30 (d, J = 4.8 Hz, 2H), 8.03 (d, J = 4.8 Hz, 2H), 7.99 (d, J = 4.8 Hz, 2H), 7.95-7.97 (m, 4H), 7.85-7.87 (m, 4H), 7.48-7.59 (m, 12H), 7.20 (s, 4H), 7.15 (s, 4H), 2.54 (s, 6H), 2.49 (s, 6H), 1.89-1.94 (m, 24H). HRMS (ESI$^+$): calcd for $C_{104}H_{78}N_8O_2Zn_2$ $[M]^+$ 1598.4831; found 1598.4846.

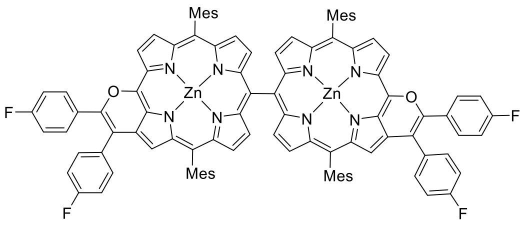

5eh: A schlenk tube with a magnetic stir bar was charged with $[Cp^*RhCl_2]_2$ (1.5 mg, 2.5 μmol), $AgSbF_6$ (3.4 mg, 10 μmol), Ag_2O (23.2 mg, 0.1 mmol), 5,15-dioxoporphyrin dimer **Zn2e** (62.4 mg, 0.05 mmol), diphenylacetylene (42.8 mg, 0.2 mmol), and DCE (0.5 mL) under N_2 atmosphere. The resulting solution was stirred at room temperature for 10 min and then at 120 ^oC for 24 h.

After being cooled down, the reaction mixture was filtered through a Celite pad, and then washed with 30 mL of CH_2Cl_2. The solvent of the filtrate was removed under reduced pressure and the residue was absorbed to small amounts of silica gel. Purification via column chromatography on neutral Al_2O_3 (petroleum ether/ethyl acetate = 6:1, v/v) afforded **5eh** as a dark green solid (36.8 mg, 44% yield). ^{1}H NMR ($CDCl_3$, 400 MHz): δ (ppm) 9.67 (d, J = 4.4 Hz, 2H), 8.76 (d, J = 4.4 Hz, 2H), 8.39 (d, J = 4.8 Hz, 2H), 8.32 (s, 2H), 8.31 (d, J = 4.8 Hz, 2H), 8.03 (d, J = 4.8 Hz, 2H), 7.99 (d, J = 4.8 Hz, 2H), 7.91-7.95 (m, 4H), 7.79-7.82 (m, 4H), 7.22-7.30 (m, 8H), 7.21 (s, 4H), 7.17 (s, 4H), 2.54 (s, 6H), 2.50 (s, 6H), 1.88-1.93 (m, 24H). HRMS (ESI$^+$): calcd for $C_{104}H_{74}F_4N_8O_2Zn_2$ $[M+H]^+$ 1671.4527; found 1671.4532.

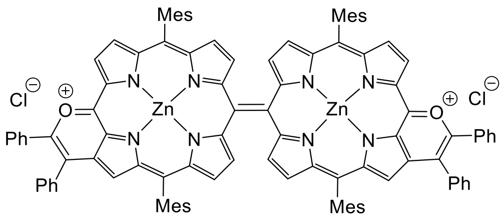

6: A flask containing **5ea** (10.0 mg, 6 μmol) was purged with argon, and then charged with dry CH_2Cl_2 (0.6 mL). A solution of $FeCl_3$ (9.5 mg, 60 μmol) and DDQ (14 mg, 60 μmol) in $MeNO_2$ (0.2 mL) was added slowly to the mixture. The mixture was stirred at room temperature for 2 h. The reaction was quenched by addition of saturated aqueous $NaHCO_3$ solution. The organic phase was separated and washed with brine, and dried over anhydrous Na_2SO_4. After removal of the solvent in vacuo, the residue was separated by silica gel chromatography eluting with petroleum ether/ethyl acetate = 1:2 (v/v) to give **6** as a dark solid (4.1 mg, 41% yield). ^{1}H NMR (CD_2Cl_2, 400 MHz): δ (ppm) 7.26-7.31 (m, 16H), 7.19-7.20 (m, 4H), 7.10-7.14 (m, 3H), 7.05-7.07 (m, 2H), 6.91-6.97 (m, 9H), 6.45 (s, 2H), 6.30 (s, 2H), 5.95 (s, 2H), 5.80 (s, 2H), 2.08-2.34 (m, 36H). HRMS (ESI$^+$): calcd for $C_{104}H_{78}N_8O_2Zn_2$ $[M]^{2+}$ 799.2410; found 799.2404.

IX. HPLC chromatograph for separation of *syn*-5ah and *anti*-5ah

Normal phase HPLC was performed firstly using different eluents such as CH_2Cl_2 /CH_3OH, ethyl ester/CH_3OH and CH_2Cl_2/*n*-hexane, and only one peak was observed in all the condition above. When CH_2Cl_2/*n*-hexane/DEA (60/40/0.1, *v*: *v*: *v*) were chosen as eluent, the mixture of **5ah** showed two peaks with peak area in a ratio of 42.3%:57.7%. The two isomers were further isolated at preparative column by using CH_2Cl_2 (0.1% DEA)/*n*-hexane as eluent.

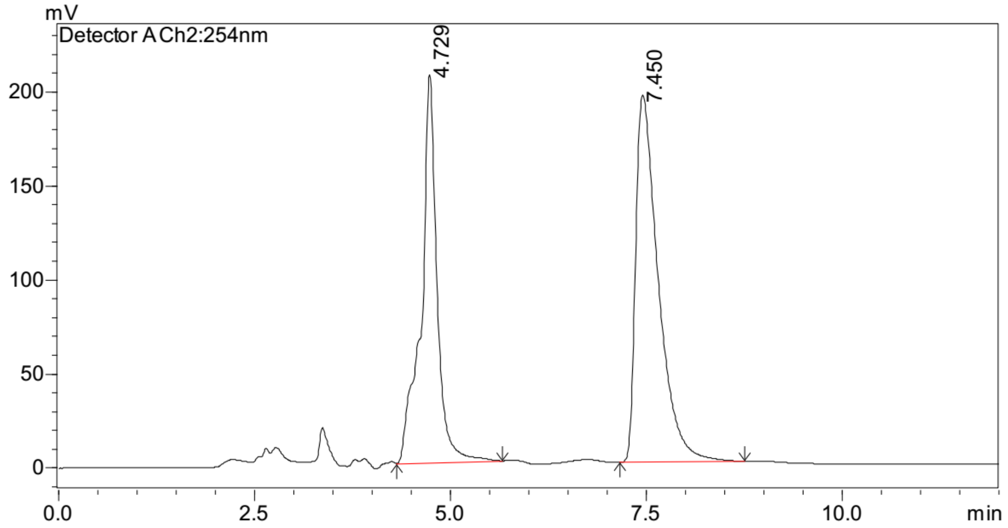

Figure S1. HPLC analysis of *syn*- and *anti*-**5ah** (CH_2Cl_2 (0.1% DEA)/*n*-hexane gradient).

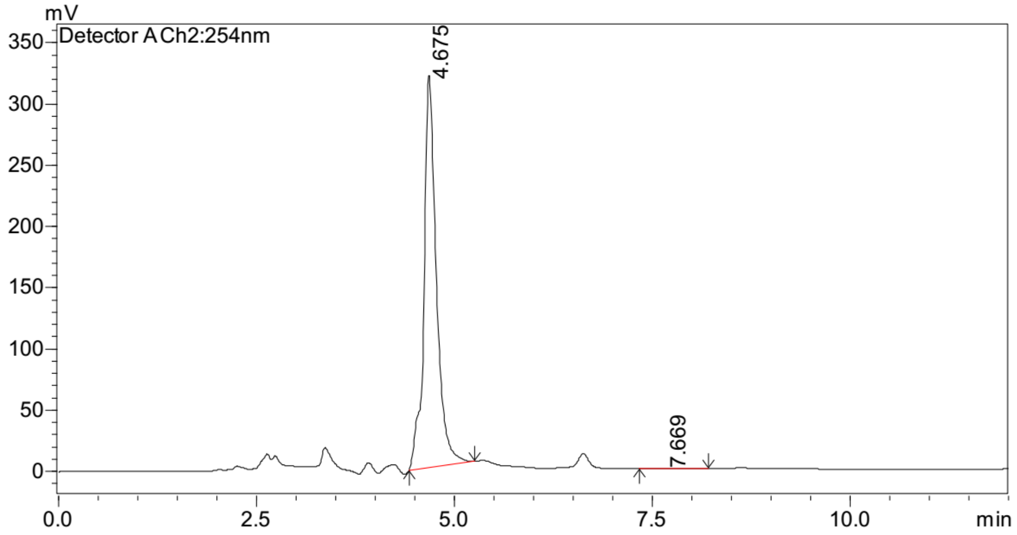

Figure S2. HPLC analysis of isolated *syn*-**5ah** (CH_2Cl_2 (0.1% DEA)/*n*-hexane gradient).

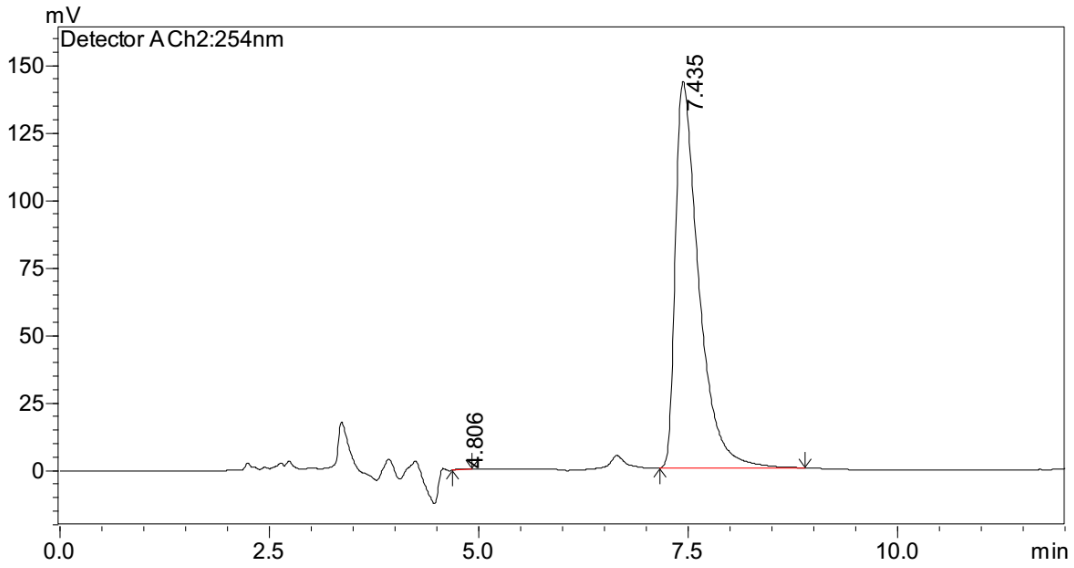

Figure S3. HPLC analysis of isolated *anti*-**5ah** (CH_2Cl_2 (0.1% DEA)/*n*-hexane gradient).

X. The concentration-dependent ^{1}H NMR spectra of the mixture of 5ah

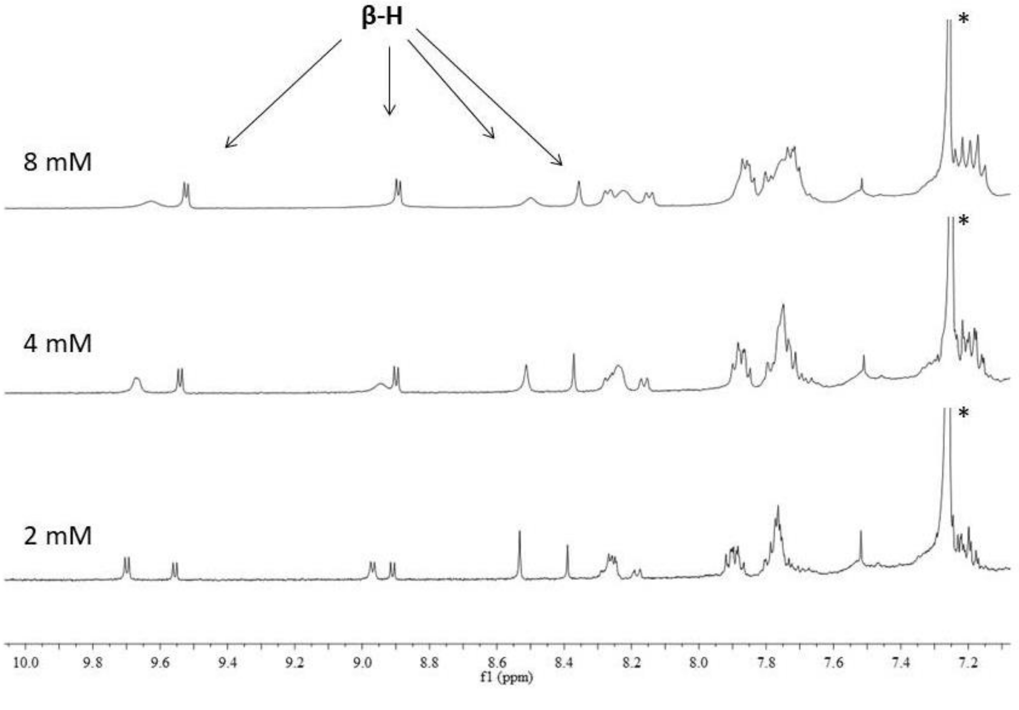

Figure S4. The concentration-dependent ^{1}H NMR spectra of **5ah** in $CDCl_3$ solution. * represent the peak of $CDCl_3$.

XI. FI-IR spectra of *syn*-5ah and *anti*-5ah

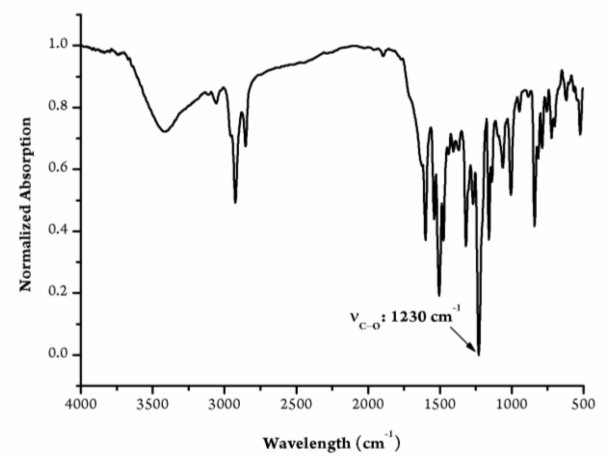

Figure S5. Normalized FI-IR spectrum of *syn*-**5ah**.

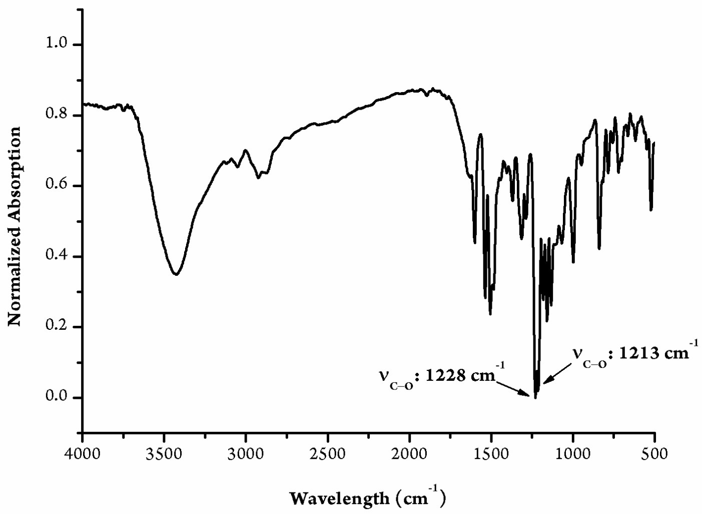

Figure S6. Normalized FI-IR spectrum of *anti*-**5ah**.

XII. DFT calculation detail

All the DFT calculations were carried out with the GAUSSIAN 09[8] series of programs. DFT method B3-LYP[9] with a standard 6–31G(d) basis set (lanl2dz[10] basis set for Zn atoms) was used for geometry optimizations. Harmonic vibrational frequency calculations were performed for all of the stationary points to confirm them as a local minima. The NICS(1) values and isosurface plots of ACID were carried out using the optimized structures.

Geometries for all the optimized compounds:

M3

C 0.45038100 0.51658200 0.12756400
C 1.89429500 0.48355100 0.03456500
C 2.33089800 1.76741200 0.19420500
C 1.16296700 2.59590500 0.39246100
N 0.02691800 1.77714900 0.34014500
H 2.50725900 -0.38899200 -0.14285300

H	3.35238000	2.12015100	0.16619800
C	-0.43569300	-0.62896500	-0.00284300
C	-1.85589400	-0.54671500	0.03747100
C	-2.65712400	-1.72929500	-0.14079300
C	-3.99643900	-1.29901600	-0.02345100
H	-4.88419100	-1.90848400	-0.09712200
C	-5.11095100	0.93010500	0.37573600
C	-5.10403200	2.30842600	0.57042800
C	-6.27065900	3.13436400	0.78646800
C	-4.39057600	4.38615100	0.84702700
H	-7.29181700	2.78116500	0.81867200
C	-3.50435600	5.53140700	0.97955000
C	-2.08601500	5.45666000	0.88863500
C	-1.28485300	6.64187000	1.04914100
C	0.01919200	4.81923000	0.72583100
C	1.16929700	3.97563400	0.57543600
N	-2.64442300	0.54190800	0.24253400
N	-3.96814700	3.12770500	0.62004100
N	-1.29761600	4.36726200	0.68722500
C	-5.83356200	4.41710600	0.95339600
C	0.05514200	6.20766500	0.95573900
C	-3.96095600	0.08908400	0.20850200
H	-6.44606400	5.28833700	1.13864500
H	0.94351400	6.81215900	1.05959800
Zn	-1.97081500	2.45376400	0.47100000
C	2.50544500	4.65462400	0.62530800
C	3.35015800	4.48767400	1.73379600
C	2.93384300	5.46823700	-0.43584200
C	4.59512900	5.12130400	1.78165400
H	3.02334000	3.86628300	2.56348300
C	4.18279800	6.09300500	-0.39269900
H	2.28804400	5.60149500	-1.29979000
C	5.01620300	5.92366000	0.71734400
H	5.23402200	4.98714200	2.65088700
H	4.50363700	6.71247600	-1.22649500
H	5.98557800	6.41414200	0.75265700
C	-6.44671200	0.25017900	0.33139400
C	-6.85813700	-0.58804300	1.38022000
C	-7.30803500	0.43981600	-0.76080400
C	-8.10630600	-1.21460000	1.34107100
H	-6.19920800	-0.74118300	2.23072500
C	-8.55269500	-0.19471200	-0.80434400
H	-6.99481500	1.08076700	-1.58085400
C	-8.95653200	-1.02178700	0.24777300
H	-8.41254200	-1.85483800	2.16449800
H	-9.20504800	-0.04145700	-1.66039100
H	-9.92560400	-1.51314800	0.21594400
C	-3.26688400	7.98448800	1.37576200
C	-1.86377000	7.91498300	1.29502800
C	-0.97271200	9.09193200	1.49688100
C	-1.02072000	9.84125200	2.68510500
C	-0.04076800	9.46134500	0.51111800
C	-0.16472100	10.92648600	2.88094700
H	-1.73087600	9.56633700	3.45981200
C	0.80928200	10.55351800	0.70207300
H	0.00496200	8.89604000	-0.41617100
C	0.75177400	11.28971500	1.88907900
H	-0.21536800	11.49001600	3.80935600
H	1.51593100	10.82858000	-0.07726900
H	1.41467800	12.13790700	2.04024000
C	-4.01177300	9.26186400	1.59111000
C	-4.96823300	9.36039600	2.61706500
C	-3.81806700	10.37588500	0.75584300
C	-5.70152200	10.53468200	2.80854400
H	-5.12508000	8.51341300	3.28119500
C	-4.55044800	11.54949200	0.94440400
H	-3.09269000	10.31810600	-0.05059500
C	-5.49482400	11.63540700	1.97243500
H	-6.42977500	10.58966600	3.61420600
H	-4.38592200	12.39708500	0.28350100
H	-6.06379800	12.55001700	2.11910500
C	-2.07627000	-3.00887600	-0.34677300
C	-0.67157600	-3.08593400	-0.37393200
C	0.07442400	-4.35729200	-0.61948500
C	1.08364400	-4.77040900	0.26797200
C	-0.16940100	-5.14319100	-1.75922500
C	1.82051600	-5.93402400	0.02985300
H	1.28045000	-4.18132500	1.16092300
C	0.56663600	-6.30484700	-2.00002200
H	-0.93644600	-4.83652200	-2.46455500
C	1.56411700	-6.70703400	-1.10596600
H	2.59088600	-6.23748200	0.73478900
H	0.36325000	-6.89456000	-2.89061500
H	2.13591800	-7.61235600	-1.29358300
C	-2.96399600	-4.19784100	-0.48012400
C	-2.84670700	-5.29139000	0.39535100
C	-3.96106400	-4.23883600	-1.47068900
C	-3.69871200	-6.39154500	0.28335400

H	-2.08533000	-5.27445300	1.16998400
C	-4.80762600	-5.34389200	-1.58979900
H	-4.06071500	-3.40474900	-2.16050500
C	-4.68086200	-6.42427800	-0.71174000
H	-3.59387100	-7.22492400	0.97371300
H	-5.56563900	-5.35962200	-2.36920900
H	-5.34116700	-7.28319700	-0.80133000
C	0.09703400	-1.91026000	-0.20271600
C	-4.03526400	6.80639000	1.22084700
H	1.17390500	-2.02211500	-0.26227300
H	-5.11243800	6.92036000	1.26888400

3aa

C	0.39315100	0.49327900	-0.02610800
C	1.82993000	0.40388300	-0.15267100
C	2.31043100	1.67095500	0.00775900
C	1.17157100	2.54139000	0.24306800
N	0.00743100	1.75745900	0.20667100
H	2.37276400	-0.50857600	-0.35211300
H	3.34100700	1.99349700	-0.04320000
C	-0.52760200	-0.63005500	-0.14410100
C	-1.93720300	-0.50465800	-0.10042200
C	-2.74362800	-1.68497500	-0.19632000
C	-4.08138400	-1.23864800	-0.06284800
H	-4.97557800	-1.84372600	-0.05765100
C	-5.16182000	1.03000100	0.26599500
C	-5.11594700	2.39961000	0.48335800
C	-6.26005900	3.28163700	0.63337500
C	-4.33781100	4.44272100	0.78449400
H	-7.29498100	2.97179800	0.58789200
C	-3.41577900	5.56027600	0.93960000
C	-2.00884900	5.44633200	0.82622600
C	-1.19858700	6.60819700	1.04029300
C	0.07972300	4.78418800	0.63268000
C	1.21791500	3.91456700	0.44690300
N	-2.70847600	0.60249100	0.06283500
N	-3.94928900	3.17468300	0.58296400
N	-1.23972600	4.35403900	0.57429300
C	-5.77957500	4.54470200	0.82206300
C	0.14018100	6.15988200	0.92589100
C	-4.02475000	0.15998300	0.08579700
H	-6.32626100	5.46564600	0.96371600
H	1.03776300	6.74539400	1.05600800
Zn	-1.97226900	2.47301600	0.35116400
C	2.56612500	4.56477700	0.49029200
C	3.44980500	4.30796000	1.55083100
C	2.96877600	5.44292100	-0.52958300
C	4.70713800	4.91673900	1.59194900
H	3.14315500	3.63782400	2.34950900
C	4.23074000	6.04058600	-0.49460700
H	2.29389400	5.64520300	-1.35698700
C	5.10253200	5.78185900	0.56783500
H	5.37612400	4.71354300	2.42426100
H	4.53213200	6.70839700	-1.29765500
H	6.08216300	6.25178400	0.59733500
C	-6.51207800	0.37872700	0.22020900
C	-7.22782500	0.13702200	1.40286600
C	-7.07923300	-0.00098500	-1.00655300
C	-8.48548600	-0.47140400	1.35988500
H	-6.79245600	0.41990700	2.35771900
C	-8.34036900	-0.60105500	-1.04958900
H	-6.53353800	0.18492500	-1.92797600
C	-9.04625500	-0.83952500	0.13365100
H	-9.02545800	-0.65598200	2.28506400
H	-8.77035600	-0.88126000	-2.00787100
H	-10.02591100	-1.30914000	0.09995400
C	-3.22083000	7.86304900	1.38534600
C	-1.80613900	7.85024000	1.34173000
C	-0.95215000	9.03034900	1.64671400
C	-1.06504100	9.70562000	2.87447900
C	0.01718900	9.46867800	0.72746000
C	-0.23595400	10.78811100	3.17216400
H	-1.80587200	9.37611900	3.59743100
C	0.84017400	10.55885600	1.02098200
H	0.11207700	8.96152000	-0.22936000
C	0.71777100	11.22189100	2.24551500
H	-0.33662700	11.29480300	4.12873400
H	1.57621300	10.88950300	0.29214700
H	1.35951900	12.06839700	2.47618800
C	-4.02181500	9.10361300	1.62259300
C	-5.10827700	9.06722300	2.51313200
C	-3.75778600	10.30138500	0.93852300
C	-5.89275900	10.20099900	2.73243900
H	-5.33211000	8.13862900	3.03012100
C	-4.54847700	11.43360100	1.14901600
H	-2.93671000	10.34728600	0.22938500
C	-5.61536100	11.39074800	2.05116100
H	-6.72329000	10.15445800	3.43287100

H	-4.33159600	12.34879900	0.60346400
H	-6.22780300	12.27347700	2.21760700
C	-2.13013500	-2.95167100	-0.34466600
C	-0.71682700	-2.95992400	-0.42103900
N	-3.96893900	6.75154200	1.20681900
N	0.02933700	-1.83828800	-0.30635600
C	0.08428200	-4.20249300	-0.64726700
C	1.24704100	-4.42525600	0.10993600
C	-0.25750900	-5.13739400	-1.63810100
C	2.03173100	-5.56068300	-0.09996300
H	1.52986000	-3.69616800	0.86357100
C	0.53233400	-6.26868400	-1.85606600
H	-1.13924900	-4.97560200	-2.25071900
C	1.67669500	-6.48909900	-1.08411400
H	2.92234400	-5.71923500	0.50355900
H	0.25383800	-6.97624700	-2.63326800
H	2.28850100	-7.37217100	-1.25108800
C	-2.97414900	-4.17745700	-0.35297700
C	-2.77925700	-5.19544700	0.59663400
C	-4.01667100	-4.32134300	-1.28536900
C	-3.60010900	-6.32414400	0.61105300
H	-1.98145200	-5.09701400	1.32741500
C	-4.83177500	-5.45602600	-1.27775500
H	-4.17627200	-3.54600400	-2.03044800
C	-4.62754700	-6.46127100	-0.32808100
H	-3.43553100	-7.09857200	1.35610300
H	-5.62592500	-5.55352100	-2.01401900
H	-5.26318000	-7.34304700	-0.31899900

4aa

C	0.48262600	0.39330600	0.05960800
C	1.89939600	0.28258000	-0.00920700
C	2.39464100	1.54174000	0.34925400
C	1.26183700	2.35333700	0.62829300
N	0.09240600	1.63670400	0.42954800
H	3.42763100	1.85458000	0.37595000
C	-0.35056600	-0.71202500	-0.21344900
C	-1.75517900	-0.65886100	-0.21998600
C	-2.53635900	-1.83685900	-0.38787700
C	-3.87296100	-1.41470200	-0.33465900
H	-4.75615900	-2.03310600	-0.38911100
C	-4.99003500	0.85192500	-0.03952700
C	-4.95988400	2.21973200	0.21565000
C	-6.10621700	3.10115400	0.20834600
C	-4.22208300	4.26380800	0.60738900
H	-7.12620300	2.79717100	0.02196300
C	-3.34235300	5.42026600	0.87188200
C	-1.89707900	5.34404000	1.13394300
C	-1.07826900	6.37433900	1.71382900
C	0.16831300	4.55659600	1.19632600
C	1.28693200	3.72185800	1.04838400
N	-2.53442400	0.44247700	-0.07019400
N	-3.81351300	2.99589300	0.47166100
N	-1.15182800	4.25211700	0.84555500
C	-5.65333600	4.36580100	0.44673900
C	0.19904600	5.88208000	1.75443100
C	-3.84065600	-0.01012300	-0.15051400
H	-6.22495000	5.27921900	0.50285600
H	1.07301200	6.37738800	2.15182900
Zn	-1.85142900	2.33915500	0.40637400
C	2.62703000	4.30487300	1.34928900
C	3.42749100	3.76833100	2.37386500
C	3.11132100	5.39851500	0.60945200
C	4.67568200	4.32374900	2.66050800
H	3.05843300	2.93143600	2.96021300
C	4.36992200	5.93728200	0.88390900
H	2.50768900	5.81101400	-0.19423900
C	5.15242200	5.40552900	1.91318500
H	5.27528900	3.91168900	3.46759600
H	4.73752300	6.77259500	0.29424900
H	6.12782000	5.83125700	2.13190400
C	-6.32759900	0.20791600	-0.21495800
C	-7.23644500	0.15392900	0.85536000
C	-6.69515200	-0.35420100	-1.44965900
C	-8.48457100	-0.45259700	0.69469600
H	-6.95654500	0.57397600	1.81776700
C	-7.95024300	-0.94409300	-1.61243700
H	-6.00368000	-0.31040700	-2.28687300
C	-8.84641000	-0.99852500	-0.54011000
H	-9.17290700	-0.49680200	1.53430700
H	-8.22796800	-1.35991700	-2.57722400
H	-9.82031400	-1.46330500	-0.66657800
C	-1.88218000	-3.10524300	-0.38304000
C	-0.49846500	-3.14377900	-0.37269500
N	-4.04674800	6.52682700	0.83264500
N	0.26009200	-1.93821600	-0.47070100
C	0.23517000	-4.39451000	-0.02378600
C	0.97471000	-4.42452800	1.17190500

C	0.11716200	-5.56925500	-0.77983400
C	1.58859000	-5.60362600	1.59672200
H	1.05905300	-3.52527800	1.77784900
C	0.73582600	-6.74589600	-0.35676300
H	-0.44935800	-5.55890300	-1.70514800
C	1.47242600	-6.76821700	0.83150600
H	2.14975500	-5.61415800	2.52745300
H	0.64146100	-7.64680900	-0.95675100
H	1.94948400	-7.68732000	1.16076200
C	-2.73235400	-4.33087800	-0.26910200
C	-2.82474000	-5.04608200	0.93441200
C	-3.50944400	-4.73696600	-1.36515000
C	-3.66906500	-6.15391100	1.03464300
H	-2.23212000	-4.73740100	1.79109300
C	-4.34868200	-5.85024000	-1.26576700
H	-3.44911200	-4.18599200	-2.30093500
C	-4.43031200	-6.56121800	-0.06523800
H	-3.73044500	-6.69976000	1.97233200
H	-4.93717100	-6.15988500	-2.12542600
H	-5.08337400	-7.42609200	0.01354400
O	-3.36848600	7.68054300	1.01938700
C	-4.26593700	8.80575500	0.91598200
H	-5.03688600	8.74022500	1.68838200
H	-3.63604700	9.68241300	1.07293400
H	-4.71827200	8.82976800	-0.07903500
C	2.47330800	-0.90846700	-0.55700100
C	1.64689100	-1.97883700	-0.83476800
C	2.09030100	-3.09107800	-1.72427400
C	3.19218600	-3.90290200	-1.41981400
C	1.44985600	-3.24920900	-2.96625400
C	3.63798000	-4.85905200	-2.33272700
H	3.69429600	-3.79345300	-0.46425200
C	1.89916900	-4.20363200	-3.87994100
H	0.60507800	-2.61408200	-3.22310700
C	2.99470200	-5.01331700	-3.56439000
H	4.48927400	-5.48524600	-2.08024000
H	1.39943000	-4.30868200	-4.83936600
H	3.34645600	-5.75662700	-4.27469100
C	3.92465200	-0.88149500	-0.91906900
C	4.89158900	-0.90206500	0.09807500
C	4.34163800	-0.76590500	-2.25363700
C	6.25191100	-0.82929800	-0.21499400
H	4.57860900	-0.98613000	1.13634100
C	5.70083700	-0.68774600	-2.56482900
H	3.60174600	-0.74266900	-3.04891400
C	6.65961100	-0.72272700	-1.54762800
H	6.99040000	-0.85662900	0.58195300
H	6.01022600	-0.60135200	-3.60299500
H	7.71682900	-0.66582400	-1.79204100
H	-1.41628800	7.33634300	2.05895200

***syn*-M4**

C	0.45366100	0.41667400	0.18434700
C	1.89517500	0.38426000	0.14545500
C	2.33344500	1.66539300	0.34744300
C	1.16253600	2.49392400	0.51909600
N	0.03669800	1.70533800	0.41107900
H	2.50912800	-0.49066000	-0.02307400
H	3.35721400	2.01223600	0.36758600
C	-0.40477700	-0.69954000	0.00721600
C	-1.79843200	-0.64999900	0.01823700
C	-2.61574600	-1.83509300	-0.18399800
C	-3.92306300	-1.41906300	-0.09884700
H	-4.81207300	-2.02436300	-0.19787300
C	-5.04853500	0.82788700	0.27955800
C	-5.02366900	2.22582800	0.48902000
C	-6.18649300	3.06785200	0.66225800
C	-4.28509100	4.28615200	0.80180800
H	-7.21120500	2.72619800	0.65822200
C	-3.48407700	5.41106500	0.99708800
C	-2.06489500	5.41376000	0.99674400
C	-1.22547700	6.56578100	1.21805900
C	0.04089700	4.71998900	0.87709500
C	1.17586400	3.88903100	0.74274600
N	-2.60054000	0.44151500	0.21259800
N	-3.88511100	2.99628000	0.58089100
N	-1.27297700	4.31037800	0.79302000
C	-5.73653500	4.35352800	0.84674100
C	0.07329600	6.13854200	1.14810200
C	-3.90591500	0.00598600	0.14611100
H	0.96690800	6.73303900	1.27756000
Zn	-1.92125400	2.36833500	0.49827000
C	2.51965500	4.54915500	0.84970100
C	3.32326800	4.35953400	1.98563300
C	3.00016000	5.37067500	-0.18298200
C	4.57339000	4.97646000	2.08835100
H	2.96006300	3.73018300	2.79416300
C	4.25200200	5.98473200	-0.08398400

H	2.38952900	5.52129100	-1.06963100
C	5.04240000	5.79056400	1.05288100
H	5.17882100	4.82176600	2.97812300
H	4.60906400	6.61332700	-0.89611000
H	6.01513400	6.26953200	1.13115000
C	-6.39117900	0.16492300	0.18815500
C	-6.86682200	-0.64550900	1.23197600
C	-7.20036700	0.33699500	-0.94691300
C	-8.11641400	-1.26565900	1.14463500
H	-6.25245500	-0.78471600	2.11802400
C	-8.44963200	-0.28342700	-1.03740400
H	-6.84141900	0.95683900	-1.76495800
C	-8.91205600	-1.08706700	0.00904200
H	-8.46755600	-1.88736800	1.96459600
H	-9.05934700	-0.14054800	-1.92638400
H	-9.88360900	-1.56993200	-0.05973200
C	-2.03187400	-3.15049200	-0.40314100
C	-0.67439500	-3.25988000	-0.41963600
C	0.04874500	-4.53709300	-0.68314300
C	1.03629000	-5.00528100	0.20325500
C	-0.19881300	-5.28213600	-1.85041500
C	1.73755000	-6.18510100	-0.05728000
H	1.24346700	-4.45644100	1.11889000
C	0.50649000	-6.45726200	-2.11601700
H	-0.94744100	-4.93242100	-2.55501300
C	1.47644500	-6.91607600	-1.21965800
H	2.48636500	-6.53303600	0.65015700
H	0.29867100	-7.01374000	-3.02664000
H	2.02416500	-7.83213200	-1.42561000
C	-2.96207700	-4.31144600	-0.55222600
C	-2.95786300	-5.36240900	0.37851800
C	-3.88569800	-4.35676900	-1.61013300
C	-3.84406300	-6.43468600	0.25059500
H	-2.25475800	-5.33577200	1.20652300
C	-4.76858200	-5.43133000	-1.74315100
H	-3.90517800	-3.54862500	-2.33726100
C	-4.75134400	-6.47454200	-0.81218300
H	-3.82495300	-7.23886400	0.98210000
H	-5.47052200	-5.45211700	-2.57329000
H	-5.44037500	-7.30933000	-0.91290700
H	-1.56491300	7.57554600	1.40784400
C	-6.44710500	5.60151500	1.08489100
C	-5.71603000	6.73582400	1.26989700
C	-7.93993500	5.55405700	1.15410400
C	-8.61775000	5.89982700	2.33383800
C	-8.69488700	5.13000400	0.04752900
C	-10.01142300	5.83496400	2.40349300
H	-8.04622200	6.22024500	3.20058900
C	-10.08920000	5.07098000	0.11310300
H	-8.18537100	4.85353300	-0.87234000
C	-10.75296700	5.42342000	1.29227000
H	-10.51729000	6.10626200	3.32686600
H	-10.65627100	4.74828000	-0.75675700
H	-11.83766000	5.37449700	1.34526700
C	-6.31979000	8.08484500	1.46783200
C	-5.95411600	8.88720700	2.56454900
C	-7.23306500	8.61333500	0.53757600
C	-6.49683500	10.16291200	2.73660000
H	-5.25753200	8.50331300	3.30619400
C	-7.77022700	9.89114000	0.70380200
H	-7.51915700	8.01627200	-0.32318100
C	-7.40684000	10.67150600	1.80558000
H	-6.20891400	10.75781600	3.59999300
H	-8.47260200	10.27763400	-0.03063400
H	-7.82677600	11.66555900	1.93622100
C	0.23083700	-2.05885600	-0.22124300
H	0.89322600	-2.00741400	-1.09916900
H	0.91125800	-2.28069900	0.61449800
C	-4.19929700	6.72798000	1.23897300
H	-3.83933300	7.16144800	2.18380700
H	-3.88846000	7.45675300	0.47429000

syn-**5aa**

C	0.26290500	0.32954100	0.16709700
C	1.69514000	0.27732800	0.13252700
C	2.13580200	1.55976300	0.34761900
C	0.97166900	2.39546900	0.51953700
N	-0.15984500	1.61138100	0.39950000
H	2.28884800	-0.60874200	-0.04618100
H	3.16149300	1.90055400	0.37344800
C	-0.63351500	-0.75247100	-0.01906600
C	-2.02375900	-0.71196600	-0.03395400
C	-2.79884300	-1.90864200	-0.25347200
C	-4.12334400	-1.51448500	-0.19432800
H	-4.99876700	-2.13581400	-0.31290800
C	-5.27969400	0.72977400	0.17106700
C	-5.25101800	2.12869400	0.39054200
C	-6.39466000	2.98934900	0.56642500

C	-4.47576800	4.16027700	0.72735700
H	-7.42664500	2.67050200	0.55536100
C	-3.66662700	5.27062400	0.94578500
C	-2.25029500	5.31937700	0.97075300
C	-1.43190000	6.47211300	1.20905000
C	-0.15137000	4.62582300	0.87598100
C	0.98356900	3.79079500	0.75114600
N	-2.82841500	0.36421800	0.14852600
N	-4.09073700	2.88053000	0.49650000
N	-1.46630400	4.21413000	0.77103300
C	-5.91352700	4.26992200	0.77029400
C	-0.12922000	6.04175100	1.15453600
C	-4.13358600	-0.09389100	0.05361800
H	0.76086600	6.63807400	1.29916500
Zn	-2.11979900	2.27606200	0.45352200
C	2.32679600	4.44681300	0.87758600
C	3.12846500	4.22609700	2.00950100
C	2.80987700	5.29505800	-0.13224700
C	4.37865700	4.83924200	2.13069600
H	2.76331900	3.57599200	2.80046000
C	4.06219800	5.90466200	-0.01494700
H	2.20123000	5.46923600	-1.01584600
C	4.85028200	5.67987900	1.11786800
H	4.98224700	4.66084100	3.01727800
H	4.42148700	6.55366800	-0.80983400
H	5.82335100	6.15560000	1.21047300
C	-6.62128900	0.07261000	0.05606300
C	-7.09635100	-0.78090000	1.06601200
C	-7.43509400	0.29328400	-1.06797800
C	-8.34715300	-1.39498100	0.95666200
H	-6.48022200	-0.95720700	1.94414800
C	-8.68572200	-0.32058600	-1.18049900
H	-7.07762100	0.94607800	-1.86057400
C	-9.14643800	-1.16728500	-0.16765000
H	-8.69710700	-2.04943400	1.75136600
H	-9.29782100	-0.13965400	-2.06095200
H	-10.11917300	-1.64510400	-0.25356300
C	-2.11263100	-3.16275500	-0.44116100
C	-0.73973700	-3.13321800	-0.43571200
C	0.19724800	-4.26146700	-0.64116100
C	1.36936100	-4.34428100	0.13323000
C	-0.03254500	-5.23911100	-1.62473900
C	2.27445500	-5.38922400	-0.05627600
H	1.56351200	-3.58941500	0.88907100

C	0.87692300	-6.28047600	-1.81471300
H	-0.91870100	-5.17847300	-2.24752300
C	2.03123400	-6.36293500	-1.02999200
H	3.17030500	-5.44229200	0.55720000
H	0.68542300	-7.02470100	-2.58345800
H	2.73742400	-7.17556200	-1.17977300
C	-2.90617600	-4.41868900	-0.56948900
C	-2.77637200	-5.45666600	0.36771300
C	-3.84265500	-4.56276900	-1.60719800
C	-3.54883000	-6.61514600	0.26068700
H	-2.06639100	-5.35173200	1.18369600
C	-4.61426300	-5.72252000	-1.71606700
H	-3.95908200	-3.76457700	-2.33601500
C	-4.46908700	-6.75366300	-0.78286000
H	-3.43406900	-7.40800400	0.99568100
H	-5.32911900	-5.81889200	-2.52955400
H	-5.07131400	-7.65486000	-0.86537900
O	-0.03672200	-1.97299400	-0.22601900
H	-1.79362400	7.47460800	1.39255400
C	-6.50986500	5.55487400	1.03720200
C	-5.65828400	6.61820000	1.20791500
O	-4.29374800	6.47303100	1.16996100
C	-7.99117800	5.66168700	1.17046800
C	-8.58334400	6.07533800	2.37471800
C	-8.82614700	5.29836400	0.10027700
C	-9.97276400	6.14061300	2.50067700
H	-7.94921600	6.34484000	3.21508400
C	-10.21604300	5.36675100	0.22441900
H	-8.38145500	4.96945900	-0.83564400
C	-10.79441500	5.78953700	1.42526900
H	-10.41296500	6.46270700	3.44112000
H	-10.84622100	5.08818400	-0.61666000
H	-11.87581300	5.83967800	1.52369900
C	-6.01055100	8.03627700	1.44866900
C	-5.26402200	8.79526500	2.36885700
C	-7.05494000	8.66254300	0.74667600
C	-5.56887700	10.13789700	2.59580400
H	-4.44885400	8.32533100	2.91071800
C	-7.35397700	10.00693900	0.97222000
H	-7.62695700	8.09946800	0.01680200
C	-6.61622200	10.74915200	1.89951200
H	-4.98754700	10.70654300	3.31719800
H	-8.16131400	10.47637300	0.41601200
H	-6.85205100	11.79575700	2.07413600

anti-**5aa**

C	0.34972400	0.43935100	0.04588100
C	1.77831200	0.34161200	-0.06330900
C	2.26744900	1.61199800	0.09399400
C	1.13748100	2.49217200	0.30547800
N	-0.02095200	1.74338800	0.26659800
H	2.33449600	-0.56694500	-0.25035100
H	3.30259100	1.92175000	0.05608400
C	-0.58295400	-0.61250100	-0.06427500
C	-1.97639200	-0.52754100	-0.00597100
C	-2.79715500	-1.70060000	-0.15669100
C	-4.10580500	-1.26254200	-0.03907100
H	-5.00461800	-1.85822200	-0.09957500
C	-5.16135800	1.03096100	0.33923500
C	-5.08001800	2.43103400	0.53143600
C	-6.20992700	3.31128000	0.74335200
C	-4.29165400	4.48270900	0.79953600
H	-7.24565200	3.00276500	0.77413800
C	-3.35882700	5.53375600	0.91589200
C	-1.96647100	5.45370000	0.82812000
C	-1.14414600	6.62153700	1.00938300
C	0.11473900	4.76411800	0.65885100
C	1.21870300	3.89185900	0.50000400
N	-2.73545600	0.57619300	0.19347900
N	-3.92142200	3.17953000	0.57310000
N	-1.20805600	4.35169400	0.61627400
C	-5.72048600	4.58093000	0.90513600
C	0.16483200	6.17987400	0.91066100
C	-4.05735600	0.15909700	0.17774400
H	-6.27771500	5.49146900	1.07891000
H	1.06502500	6.76735100	1.01636600
Zn	-1.97144000	2.46315600	0.41009400
C	2.58498900	4.50678200	0.54908600
C	3.43798900	4.27876800	1.64170200
C	3.04065300	5.32823800	-0.49558100
C	4.71008700	4.85592900	1.68991000
H	3.09527200	3.65048700	2.45996100
C	4.31385600	5.90295800	-0.45128400
H	2.39316800	5.50744100	-1.35026800
C	5.15284400	5.66954200	0.64266100
H	5.35332000	4.67109700	2.54684500
H	4.65061600	6.53047600	-1.27297900
H	6.14271000	6.11750100	0.67854800
C	-6.52758000	0.41472100	0.30115800
C	-6.97590800	-0.40573700	1.34975300
C	-7.38737800	0.63996900	-0.78681700
C	-8.24839300	-0.98264600	1.31350900
H	-6.32281400	-0.58469600	2.20012400
C	-8.65937100	0.06184200	-0.82619600
H	-7.05064300	1.26818000	-1.60768100
C	-9.09453200	-0.75134700	0.22464900
H	-8.57829400	-1.61114600	2.13714800
H	-9.30851500	0.24595800	-1.67886900
H	-10.08413400	-1.20042300	0.19556400
C	-3.14534800	7.91342900	1.31811800
C	-1.77439900	7.89126300	1.28038400
C	-0.92606300	9.08465800	1.56163900
C	-0.99421200	9.74766400	2.79779200
C	-0.00072500	9.53771000	0.60596400
C	-0.17230500	10.84457300	3.06531000
H	-1.69497300	9.39836400	3.55126900
C	0.81999600	10.63658800	0.87192900
H	0.06769400	9.03028500	-0.35305400
C	0.73614800	11.29507100	2.10261200
H	-0.23992400	11.34509800	4.02805000
H	1.52516800	10.97702400	0.11761600
H	1.37624300	12.14852700	2.31115400
C	-4.03182300	9.07950800	1.53568200
C	-5.17572300	8.94583200	2.34424300
C	-3.78121200	10.31801800	0.92008200
C	-6.03164000	10.02853500	2.54983000
H	-5.38563000	7.99115800	2.81696700
C	-4.64176700	11.39764500	1.12390900
H	-2.91745900	10.43322200	0.27378700
C	-5.76745800	11.25971500	1.94168500
H	-6.90544600	9.90980800	3.18550800
H	-4.43480600	12.34671600	0.63609800
H	-6.43553400	12.10251300	2.09936000
C	-2.16406000	-2.98320900	-0.34828400
C	-0.79394600	-3.00107900	-0.41446300
C	0.09372000	-4.16538200	-0.63611900
C	1.30492700	-4.26411300	0.07375000
C	-0.22230100	-5.16454300	-1.57315500
C	2.16394200	-5.34432300	-0.13181800
H	1.56572500	-3.49323800	0.79240300
C	0.64093300	-6.24155600	-1.77919700
H	-1.13911900	-5.09301500	-2.14849100

C	1.83477200	-6.33901700	-1.05788700
H	3.09101000	-5.40853000	0.43222800
H	0.38237400	-7.00200900	-2.51157900
H	2.50493100	-7.17926500	-1.22022900
C	-3.00652100	-4.21236700	-0.40168300
C	-2.87182200	-5.22507400	0.56204200
C	-3.99302500	-4.35363900	-1.39236400
C	-3.68862800	-6.35726400	0.52595100
H	-2.12256100	-5.12147500	1.34232800
C	-4.80911200	-5.48716000	-1.43029500
H	-4.11327400	-3.57449300	-2.14097600
C	-4.65873000	-6.49397300	-0.47168800
H	-3.56889600	-7.13118300	1.28009700
H	-5.56232400	-5.58228100	-2.20857100
H	-5.29496100	-7.37493200	-0.49928700
O	-3.89932900	6.77702300	1.14747100
O	-0.04176100	-1.85947000	-0.27222000

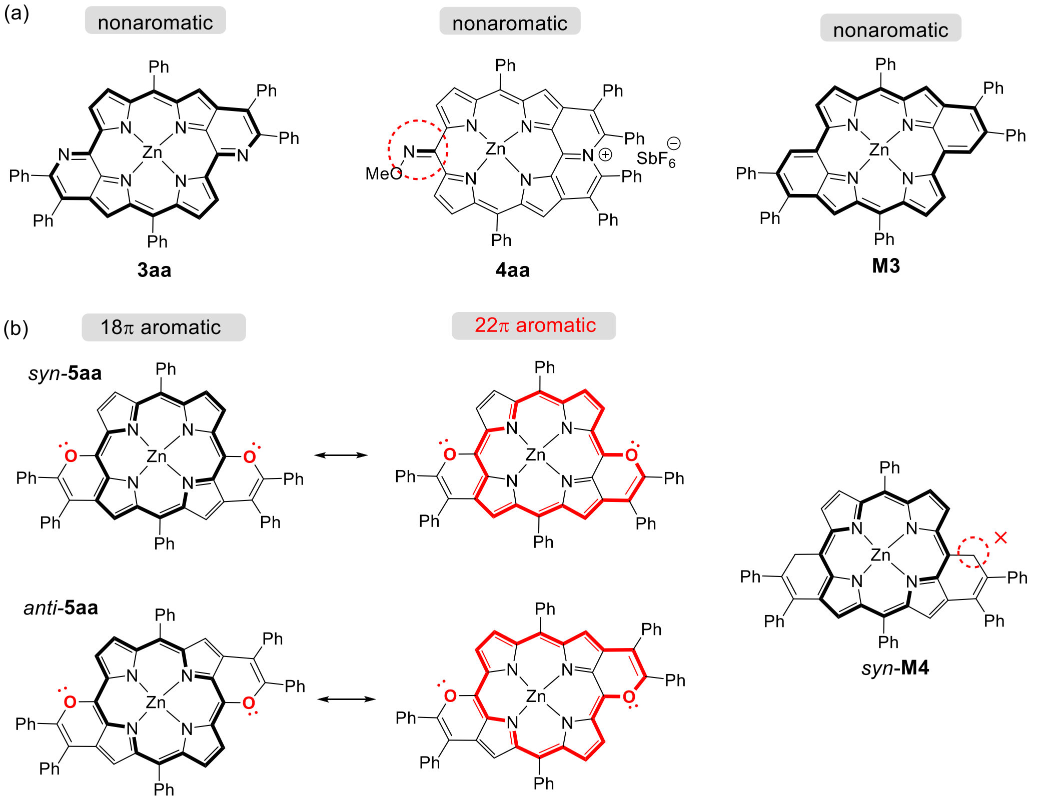

Scheme S1. The aromaticity of **3aa**, **4aa**, simulated **M3** and *syn*-**M4,** and the resonance structures of *syn*-**5aa** and *anti*-**5aa**.

XIII. Optical properties of the representative products

Table S3. UV-*vis*-NIR absorption spectra measured in CH_2Cl_2 solution (4.0 × 10^{-5} M).

Product	λ_{abs} /nm (ε /10^3 M^{-1} cm^{-1})		
	B	Q	
**3aa**	448 (51.6)		631 (28.4)
**3ad**	454 (50.2)	-	642 (32.0)
**3aj**	444 (54.0)	-	624 (30.9)
**3ak**	441 (52.2)	-	616 (24.9)
**3ba**	448 (71.6)	-	632 (39.0)
**4aa**	461 (55.0)	596 (8.4)	730 (5.7)
**4al**	467 (13.4)	629 (3.0)	751 (1.8)
**4ah**	466 (28.3)	625 (5.7)	749 (3.3)
**4an**	460 (11.4)	600 (1.6)	738 (1.0)

Table S4. Photophysical data of *syn*-**5ah**, *anti*-**5ah**, **5ea**, **5eh** and **6** measured in CH_2Cl_2 solution (1.0×10^{-5} M).

Product	λ_{abs} /nm (ε / 10^4 M^{-1} cm^{-1})
syn-**5ah**	456 (7.98), 582 (0.89), 631, (1.93), 741 (0.57)
anti-**5ah**	448 (15.19), 548 (0.99), 587 (1.16), 612 (0.93), 670, (3.70), 881(0.40)
**5ea**	467 (20.26), 593 (2.37), 624 (2.69)
**5eh**	466 (21.40), 592 (2.47), 622 (2.77)
**6**	548 (8.44), 622 (9.02), 799 (2.48), 1005 (2.82), 1113 (5.0)

XIV. Cyclic voltammogram measurements

General procedure: Cyclic voltammogram measurements were conducted on LK2005A using a platinum plate working electrode, an Ag/Ag^+ (0.01 M of $AgNO_3$ in CH_3CN) reference electrode, and a platinum wire counter electrode. Electrochemical experiments were carried out in dry HPLC grade CH_3CN or CH_2Cl_2 using ferrocene/ferrocenium as reference. Unless otherwise stated, the cyclic voltammogram measurements were performed at a scan rate of 50 mV/s and the supporting electrolyte was tetrabutylammonium hexafluorophosphate ($TBAPF_6$, 0.1 M) in all cases.

Table S5. Electrochemical properties of **3aa**, **4aa**, *syn*-**5ah** and *anti*-**5ah**.

Compound	$E_{ox, 1}$	$E_{ox, 2}$	$E_{ox, 3}$	$E_{red, 1}$	$E_{red, 2}$	$E_{red, 3}$	$E_{ox, 1} - E_{red, 1}$
**3aa**	0.30	0.55					
**4aa**	0.34	0.63	0.97	-0.76	-1.06	-1.45	1.10
syn-**5ah**	0.15	0.77	1.18	-1.58	-1.98		1.73
anti-**5ah**	0.07	0.49		-1.50	-1.89		1.57

$E_{ox, 1}$ and $E_{red, 1}$ are the first oxidation and reduction potentials, respectively. $E_{ox, 2}$ and $E_{red, 2}$ are the potentials of the second oxidative and reductive redox wave, respectively, with potentials *versus* Fc/Fc^+ couple. $E_{ox, 3}$ are the third oxidation potential, respectively.

XV. Quantification of singlet oxygen generation

Singlet oxygen generation studies of *syn*-**5al** and methylene blue (**MB**) were detected through monitoring the oxidation of 1,3-diphenylisobenzofuran (DPBF).[11] Specifically, an oxygen-saturated *N,N*-Dimethylformamide (DMF) solution of photosensitizer containing DPBF was prepared in the dark and irradiated with a 680 nm laser at a power of 150 mW cm^{-2} in an interval of 30 s. DPBF oxidation was monitored by UV-Vis-NIR spectrophotometer. *Syn*-**5al** exhibited good ability to sensitize the singlet oxygen ($^1O_2$), leading to obvious decrease in the absorbance of DPBF at 416 nm. The singlet oxygen quantum yields (Φ_Δ) were obtained by the relative method using methylene blue (**MB**) in DMF (Φ_Δ = 0.52) as the standard and calculated with eq. 1,[11]

$$\Phi_\Delta\,(^1O_2)^{bod} = \Phi_\Delta\,(^1O_2)^{MB}\,\frac{m^{bod}F^{MB}}{m^{MB}F^{bod}} \qquad \text{(eq. 1)}$$

where superscripts ‘bod’ and ‘MB’ represent sample and methylene blue (**MB**), respectively. $\Phi_\Delta\,[^1O_2]$ is the quantum yield of singlet oxygen, ‘m’ is the slope of a plot of difference in change in absorbance of DPBF (at 416 nm) with the irradiation time and ‘F’ is the absorption correction factor, which is given by F = 1 – 10^{-OD} (OD represents the optical density of sample and **MB** at or 680 nm).

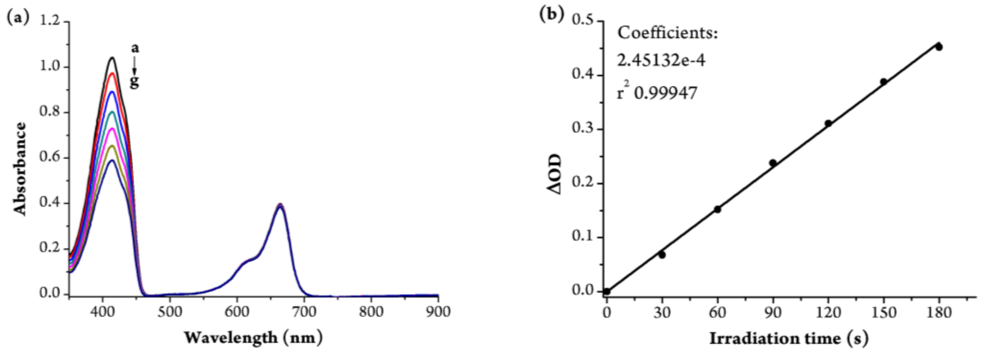

Figure S7. (a) Changes in the absorption spectrum of DPBF at 416 nm upon irradiation in the presence of **MB**. a) 0 and g) 180 s (recorded at every 30 s intervals). (b) Plot of change in absorbance of DPBF at 416 nm vs irradiation time (λ_{irr} = 635 nm) in the presence of **MB.**

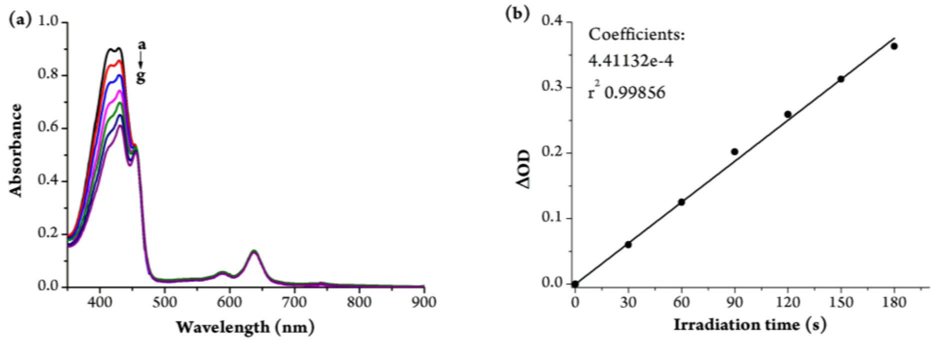

Figure S8. (a) Changes in the absorption spectrum of DPBF at 416 nm upon irradiation in the presence of *syn*-**5af**. a) 0 and g) 180 s (recorded at every 30 s intervals). (b) Plot of change in absorbance of DPBF at 416 nm vs irradiation time (λ_{irr} = 635 nm) in the presence of *syn*-**5af**.

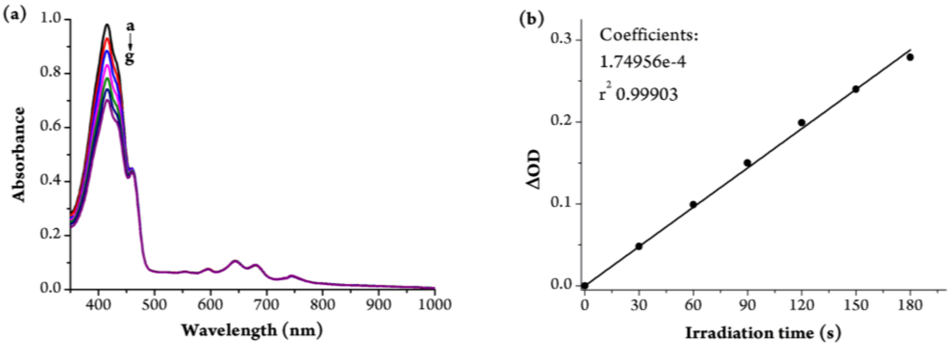

Figure S9. (a) Changes in the absorption spectrum of DPBF at 416 nm upon irradiation in the presence of *syn*-**5al**. a) 0 and g) 180 s (recorded at every 30 s intervals). (b) Plot of change in absorbance of DPBF at 416 nm vs irradiation time (λ_{irr} = 635 nm) in the presence of *syn*-**5al**.

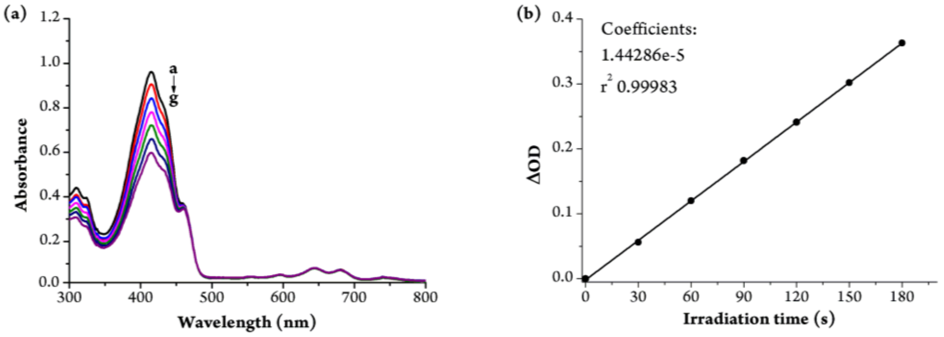

Figure S10. (a) Changes in the absorption spectrum of DPBF at 416 nm upon irradiation (λ_{irr} = 680 nm) in the presence of *syn*-**5al**. a) 0 and g) 150 s (recorded at every 30 s intervals). (b) Plot of change in absorbance of DPBF at 416 nm vs irradiation time (λ_{irr} = 680 nm) in the presence of *syn*-**5al**.

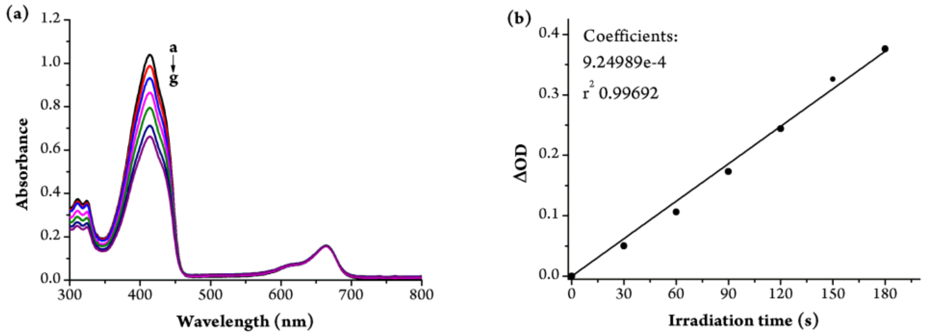

Figure S11. (a) Changes in the absorption spectrum of DPBF at 416 nm upon irradiation (λ_{irr} = 680 nm) in the presence of **MB**. a) 0 and g) 180 s (recorded at every 30 s intervals). (b) Plot of change in absorbance of DPBF at 416 nm vs irradiation time (λ_{irr} = 680 nm) in the presence of **MB**.

XVI. Single crystal X-ray structures of Zn1a, 3aa, 4ah, *syn*-5aa, *syn*-5af, *syn*- and *anti*-5ah

Table S6. Crystal Data and Structure Refinement for **Zn1a**

Identification code	**Zn1a**
Empirical formula	$C_{34}H_{24}N_6O_2Zn$
Formula weight	613.96
Temperature/K	296.4(5)
Crystal system	monoclinic
Space group	C2/c
a/Å	11.1672(4)
b/Å	27.7657(7)
c/Å	9.6241(3)
α/°	90
β/°	114.758(4)
γ/°	90
Volume/Å^3	2709.82(17)
Z	4
ρ_{calc}g/cm^3	1.505
μ/mm^{-1}	1.625
F(000)	1264.0
Crystal size/mm^3	0.45 × 0.2 × 0.1
Radiation	CuKα (λ = 1.54184)
2Θ range for data collection/°	9.284 to 145.704
Index ranges	-13 ≤ h ≤ 13, -32 ≤ k ≤ 34, -7 ≤ l ≤ 11
Reflections collected	7639
Independent reflections	2670 [R_{int} = 0.0263, R_{sigma} = 0.0250]
Data/restraints/parameters	2670/7/199
Goodness-of-fit on F^2	1.040
Final R indexes [I>=2σ (I)]	R_1 = 0.0623, wR_2 = 0.1746
Final R indexes [all data]	R_1 = 0.0706, wR_2 = 0.1856
Largest diff. peak/hole / e Å^{-3}	0.96/-0.61

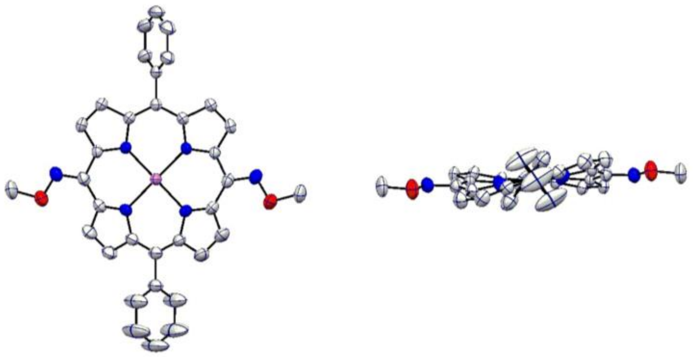

Table S7. Crystal Data and Structure Refinement for **3aa**

Identification code	**3aa**
Empirical formula	$C_{70}H_{46}N_8Zn$
Formula weight	1064.57
Temperature/K	150
Crystal system	triclinic
Space group	P-1
a/Å	10.1189(3)
b/Å	16.2647(5)
c/Å	19.3022(6)
α/°	67.268(3)
β/°	82.291(3)
γ/°	82.584(3)
Volume/Å^3	2893.15(16)
Z	1
ρ_{calc}g/cm^3	1.359
μ/mm^{-1}	2.268
F(000)	1220.0
Crystal size/mm^3	0.6 × 0.3 × 0.3
Radiation	CuKα (λ = 1.54184)
2Θ range for data collection/°	8.85 to 127.382
Index ranges	-11 ≤ h ≤ 11, -18 ≤ k ≤ 18, -22 ≤ l ≤ 18
Reflections collected	28348
Independent reflections	9511 [R_{int} = 0.0469, R_{sigma} = 0.0379]
Data/restraints/parameters	9511/0/751
Goodness-of-fit on F^2	1.352
Final R indexes [I>=2σ (I)]	R_1 = 0.0980, wR_2 = 0.2969
Final R indexes [all data]	R_1 = 0.1104, wR_2 = 0.3133
Largest diff. peak/hole / e Å^{-3}	1.90/-1.16

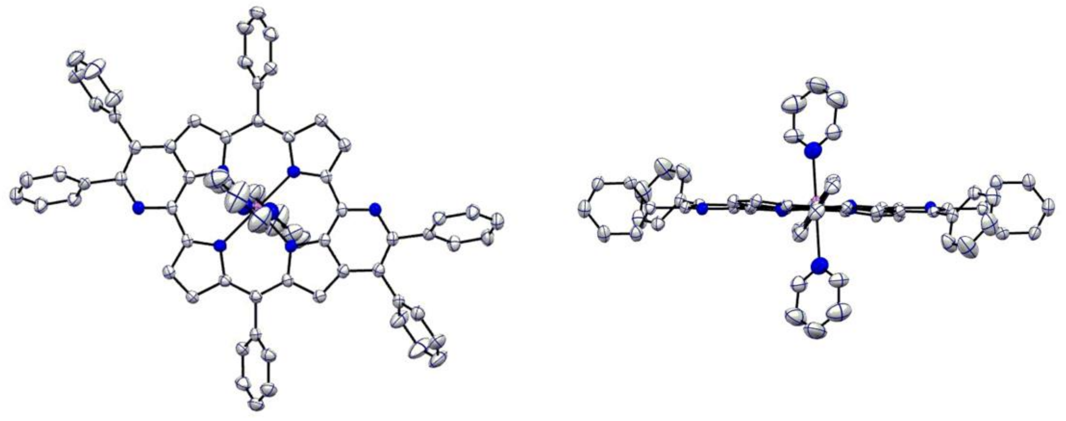

Table S8. Crystal Data and Structure Refinement for **4ah**

Identification code	**4ah**
Empirical formula	$C_{65}H_{39}F_{10}N_6O_2SbZn$
Formula weight	1313.19
Temperature/K	150
Crystal system	monoclinic
Space group	$P2_1/c$
a/Å	20.2843(2)
b/Å	17.94042(16)
c/Å	17.71991(20)
α/°	90
β/°	104.6455(12)
γ/°	90
Volume/Å^3	6238.92(12)
Z	4
ρ_{calc}g/cm^3	1.569
μ/mm^{-1}	5.419
F(000)	2976.0
Crystal size/mm^3	0.3 × 0.2 × 0.1
Radiation	CuKα (λ = 1.54184)
2Θ range for data collection/°	9.012 to 145.488
Index ranges	-24 ≤ h ≤ 23, -18 ≤ k ≤ 21, -15 ≤ l ≤ 21
Reflections collected	32809
Independent reflections	11878 [R_{int} = 0.0386, R_{sigma} = 0.0430]
Data/restraints/parameters	11878/2/860
Goodness-of-fit on F^2	1.026
Final R indexes [I>=2σ (I)]	R_1 = 0.0570, wR_2 = 0.1520
Final R indexes [all data]	R_1 = 0.0694, wR_2 = 0.1619
Largest diff. peak/hole / e Å^{-3}	1.63/-1.47

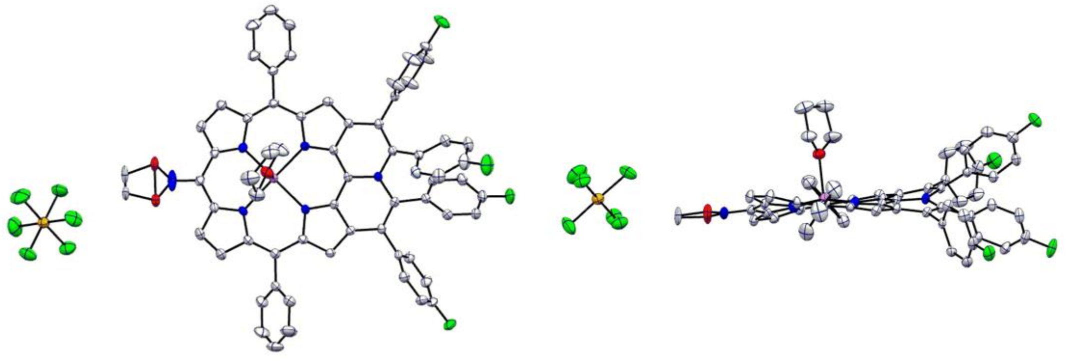

Table S9. Crystal Data and Structure Refinement for *syn*-**5aa**

Identification code	*syn*-**5aa**
Empirical formula	$C_{64}H_{44}N_4O_3Zn$
Formula weight	982.40
Temperature/K	293.75(10)
Crystal system	triclinic
Space group	P-1
a/Å	13.4002(4)
b/Å	13.4625(4)
c/Å	17.1704(5)
α/°	103.438(2)
β/°	101.949(2)
γ/°	98.725(2)
Volume/Å3	2881.73(15)
Z	2
ρcalcg/cm3	1.132
μ/mm-1	0.949
F(000)	1020.0
Crystal size/mm3	0.6 × 0.4 × 0.2
Radiation	CuKα (λ = 1.54184)
2Θ range for data collection/°	8.676 to 145.97
Index ranges	-16 ≤ h ≤ 16, -10 ≤ k ≤ 16, -21 ≤ l ≤ 21
Reflections collected	31404
Independent reflections	11234 [Rint = 0.0613, Rsigma = 0.0615]
Data/restraints/parameters	11234/237/711
Goodness-of-fit on F2	1.016
Final R indexes [I>=2σ (I)]	R1 = 0.0824, wR2 = 0.2351
Final R indexes [all data]	R1 = 0.1050, wR2 = 0.2705
Largest diff. peak/hole / e Å-3	0.86/-0.40

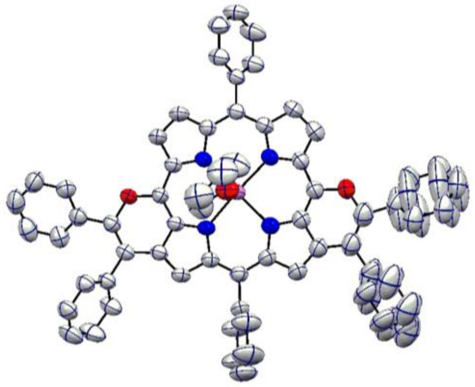

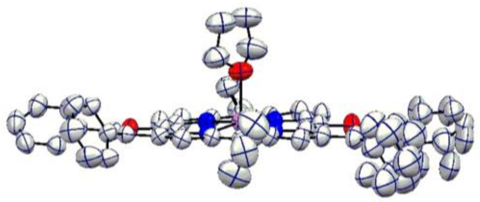

Table S10. Crystal Data and Structure Refinement for *syn*-**5af**

Identification code	*syn*-**5af**
Empirical formula	$C_{65}H_{37}Cl_4N_5O_2Zn$
Formula weight	1127.16
Temperature/K	293.15
Crystal system	monoclinic
Space group	$P2_1/c$
a/Å	13.4669(11)
b/Å	28.4544(20)
c/Å	17.1786(14)
α/°	90
β/°	109.813(9)
γ/°	90
Volume/$Å^3$	6193.0(9)
Z	4
$\rho_{calc}g/cm^3$	1.209
μ/mm^{-1}	0.614
F(000)	2304.0
Crystal size/mm^3	0.4 × 0.35 × 0.3
Radiation	MoKα (λ = 0.71073)
2Θ range for data collection/°	5.798 to 52.744
Index ranges	-16 ≤ h ≤ 16, -35 ≤ k ≤ 20, -21 ≤ l ≤ 20
Reflections collected	29117
Independent reflections	12644 [R_{int} = 0.0356, R_{sigma} = 0.0484]
Data/restraints/parameters	12644/0/694
Goodness-of-fit on F^2	1.052
Final R indexes [I>=2σ (I)]	R_1 = 0.0487, wR_2 = 0.1224
Final R indexes [all data]	R_1 = 0.0712, wR_2 = 0.1345
Largest diff. peak/hole / e $Å^{-3}$	0.52/-0.37

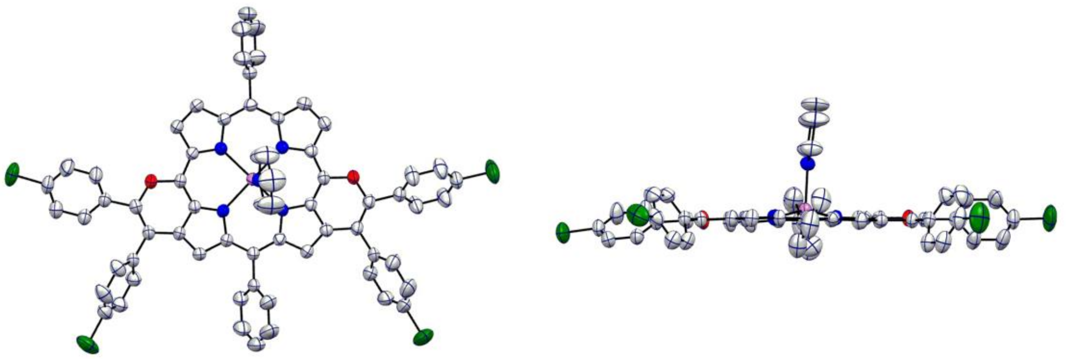

Table S11. Crystal Data and Structure Refinement for *syn*-**5ah**

Identification code	*syn*-**5ah**
Empirical formula	$C_{64}H_{40}F_4N_4O_3Zn$
Formula weight	1054.37
Temperature/K	293.15
Crystal system	triclinic
Space group	P-1
a/Å	13.4252(11)
b/Å	14.2425(13)
c/Å	14.4941(9)
α/°	102.982(7)
β/°	94.647(6)
γ/°	95.015(7)
Volume/Å^3	2675.7(4)
Z	2
ρ_{calc}g/cm^3	1.309
μ/mm^{-1}	0.523
F(000)	1084.0
Crystal size/mm^3	0.35 × 0.03 × 0.03
Radiation	MoKα (λ = 0.71073)
2Θ range for data collection/°	5.8 to 52.74
Index ranges	-16 ≤ h ≤ 15, -16 ≤ k ≤ 17, -17 ≤ l ≤ 18
Reflections collected	22340
Independent reflections	10919 [R_{int} = 0.1005, R_{sigma} = 0.2786]
Data/restraints/parameters	10919/0/685
Goodness-of-fit on F^2	0.862
Final R indexes [I>=2σ (I)]	R_1 = 0.0874, wR_2 = 0.1567
Final R indexes [all data]	R_1 = 0.2308, wR_2 = 0.2051
Largest diff. peak/hole / e Å^{-3}	0.62/-0.47

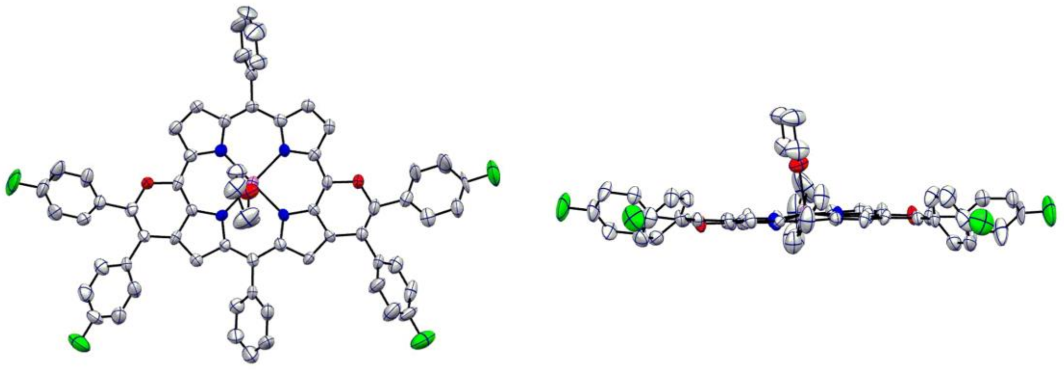

Table S12. Crystal Data and Structure Refinement for *anti*-**5ah**

Identification code	*anti*-**5ah**
Empirical formula	$C_{68}H_{48}F_4N_4O_4Zn$
Formula weight	1126.47
Temperature/K	293.15
Crystal system	triclinic
Space group	P-1
a/Å	9.8040(6)
b/Å	10.8952(7)
c/Å	13.1775(9)
α/°	73.385(6)
β/°	77.153(5)
γ/°	81.794(5)
Volume/Å^3	1310.32(15)
Z	1
ρ_{calc}g/cm^3	1.428
μ/mm^{-1}	0.541
F(000)	582.0
Crystal size/mm^3	0.35 × 0.08 × 0.03
Radiation	MoKα (λ = 0.71073)
2Θ range for data collection/°	5.872 to 52.744
Index ranges	-12 ≤ h ≤ 11, -13 ≤ k ≤ 13, -16 ≤ l ≤ 14
Reflections collected	10060
Independent reflections	5358 [R_{int} = 0.0424, R_{sigma} = 0.1166]
Data/restraints/parameters	5358/0/361
Goodness-of-fit on F^2	1.035
Final R indexes [I>=2σ (I)]	R_1 = 0.0806, wR_2 = 0.1882
Final R indexes [all data]	R_1 = 0.1428, wR_2 = 0.2328
Largest diff. peak/hole / e Å^{-3}	1.09/-1.02

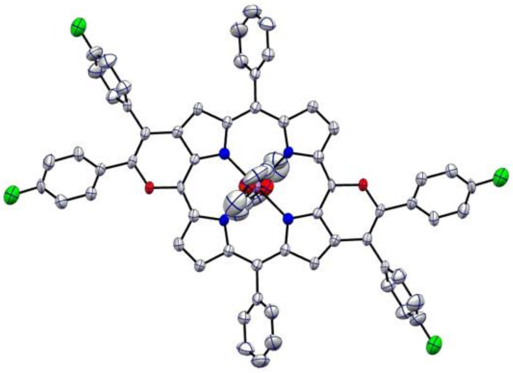

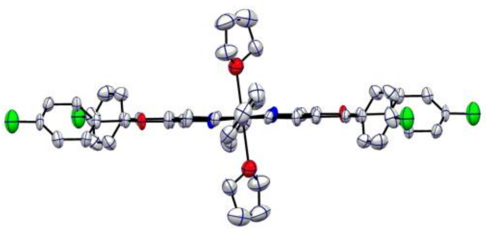

XVII. References

1. J. W. Kang, K. Moseley and P. M. Maitlis, *J. Am. Chem. Soc.* 1969, **91**, 5970.

2. N. L. Bill, M. Ishida, S. Bähring, J. M. Lim, S. Lee, C. M. Davis, V. M. Lynch, K. A. Nielsen, J. O. Jeppesen, K. Ohkubo, S. Fukuzumi, D. Kim and J. L. Sessler, *J. Am. Chem. Soc.* 2013, **135**, 10852.

3. D.-M. Shen, C. Liu, X.-G. Chen and Q.-Y. Chen, *Synlett*. 2009, **6**, 945.

4. Y. Jun-i, N. Fukui, K. Furukawa and A. Osuka, *Chem. Eur. J.* 2018, **24**, 1528.

5. A. Yella, H.-W. Lee, H. N. Tsao, C. Yi, A. K. Chandiran, M. K. Nazeeruddin, E. W.-G. Diau, C.-Y. Yeh, S. M. Zakeeruddin and M. Grätzel, *Science* 2011, **334**, 629.

6. C.-H. Yeh, W.-C. Chen, P. Gandeepan, Y.-C. Hong, C.-H. Shih and C.-H. Cheng, *Org. Biomol. Chem.* 2014, **12**, 9105.

7. M. O. Senge, Y. M. Shaker, M. Pintea, C. Ryppa, S. S. Hatscher, A. Ryan and Y. Sergeeva, *Eur. J. Org. Chem.* 2010, 237.

8. M. J. Frisch, G. W. Trucks, H. B. Schlegel, G. E. Scuseria, M. A. Robb, J. R. Cheeseman, G. Scalmani, V. Barone, B. Mennucci, G. A. Petersson, H. Nakatsuji, M. Caricato, X. Li, H. P. Hratchian, A. F. Izmaylov, J. Bloino, G. Zheng, J. L. Sonnenberg, M. Hada, M. Ehara, K. Toyota, R. Fukuda, J. Hasegawa, M. Ishida, T. Nakajima, Y. Honda, O. Kitao, H. Nakai, T. Vreven, J. A. Jr. Montgomery, J. E. Peralta, F. Ogliaro, M. Bearpark, J. J. Heyd, E. Brothers, K. N. Kudin, V. N. Staroverov, R. Kobayashi, J. Normand, K. Raghavachari, A. Rendell, J. C. Burant, S. S. Iyengar, J. Tomasi, M. Cossi, N. Rega, N. J. Millam, M. Klene, J. E. Knox, J. B. Cross, V. Bakken, C. Adamo, J. Jaramillo, R. Gomperts, R. E. Stratmann, O. Yazyev, A. J. Austin, R. Cammi, C. Pomelli, J. W. Ochterski, R. L. Martin, K. Morokuma, V. G. Zakrzewski, G. A. Voth, P. Salvador, J. J. Dannenberg, S. Dapprich, A. D. Daniels, Ö. Farkas, J. B. Foresman, J. V. Ortiz, J. Cioslowski and D. J. Fox, *Gaussian 09*, Revision

D.01, Gaussian, Inc., Wallingford CT, 2009.

9. (a) A. D. Becke, *J. Chem. Phys*. 1993, **98**, 5648; (b) C. Lee, W. Yang and R. G. Parr, *Phys. Rev. B.* 1988, **37**, 785.

10. (a) P. J. Hay and W. R. Wadt, *J. Chem. Phys.* 1985, **82**, 270; (b) W. R. Wadt and P. J. Hay, *J. Chem. Phys.* 1985, **82**, 284; (c) P. J. Hay and W. R. Wadt, *J. Chem. Phys.* 1985, **82**, 299.

11. J. Tian, L. Ding, H.-J. Xu, Z. Shen, H. Ju, L. Jia, L. Bao and J.-S. Yu, *J. Am. Chem. Soc.* 2013, **135**, 18850.

XVIII. Copies of NMR spectra

^{1}H NMR spectra of **H_2DPP 1c** ($CDCl_3$)

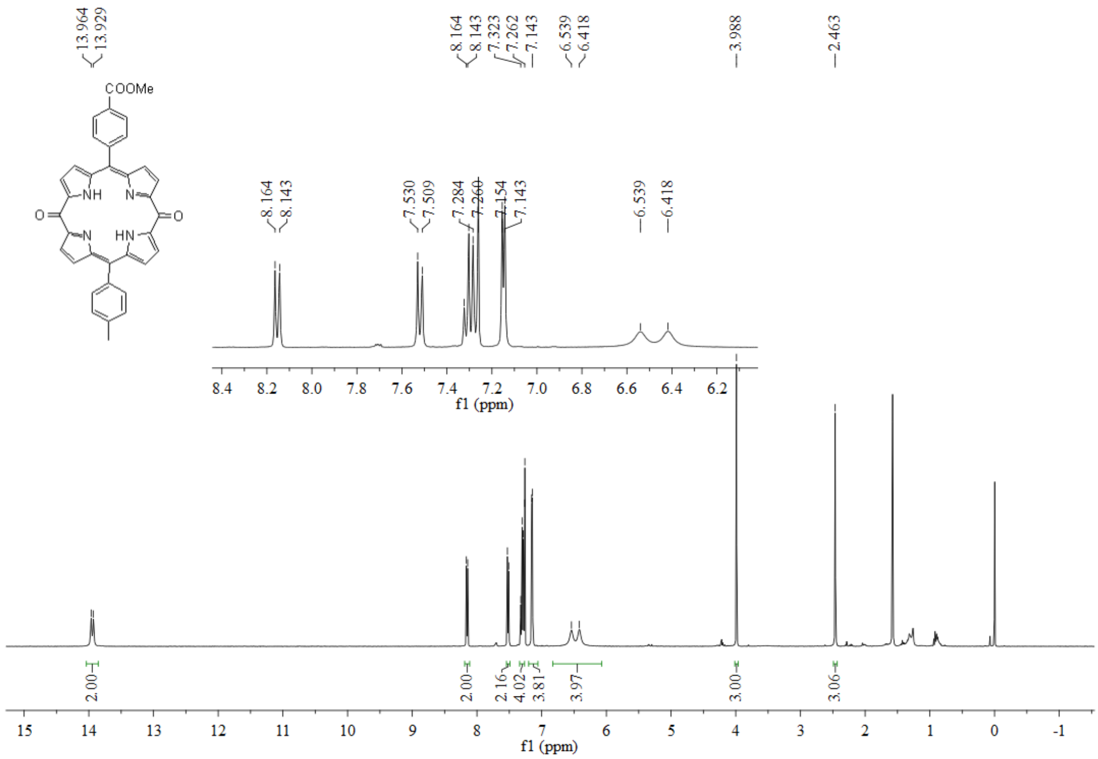

^{1}H NMR spectra of **H_2DPP 2a** ($CDCl_3$)

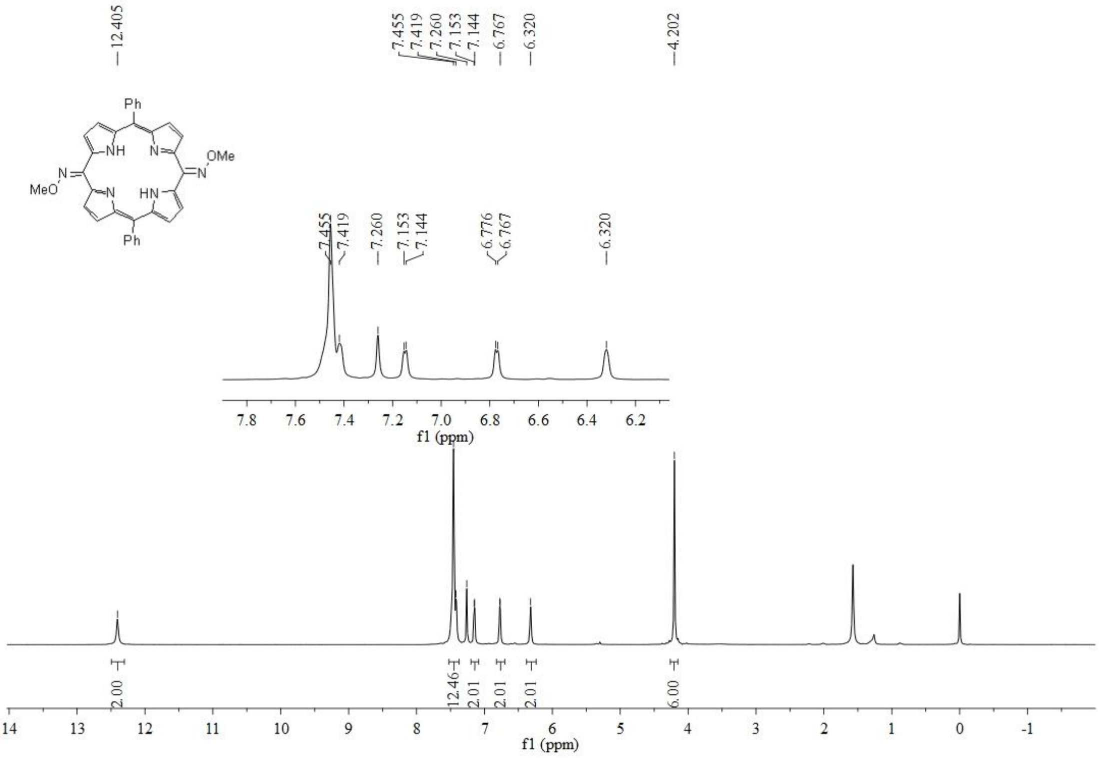

^{1}H NMR spectra of **H_2DPP 2b** ($CDCl_3$)

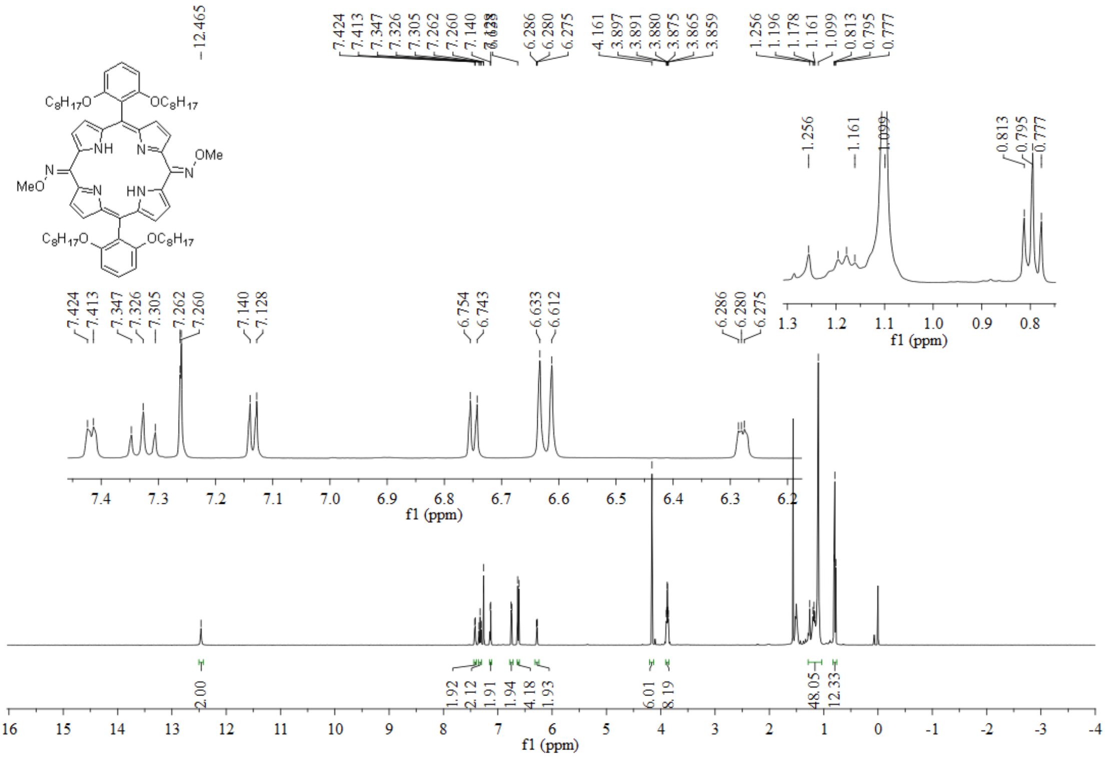

^{1}H NMR spectra of **H_2DPP 2d** ($CDCl_3$)

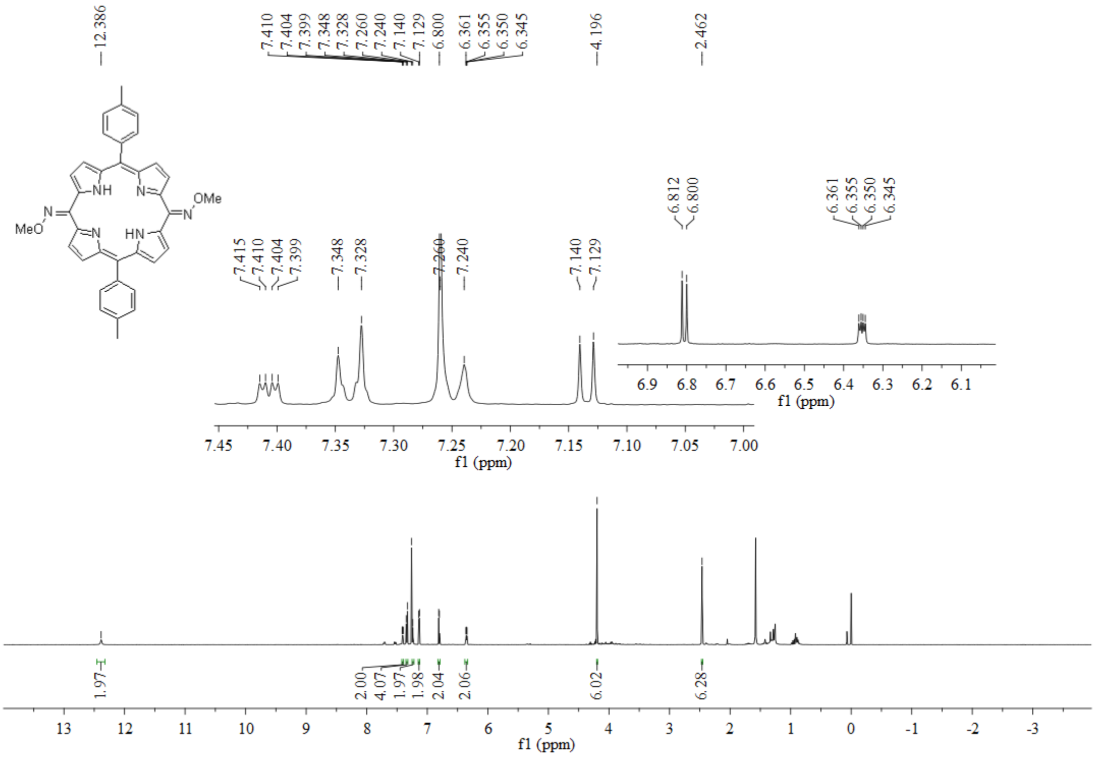

^{1}H NMR spectra of **Zn1a** ($CDCl_3$), * represent solvent peak.

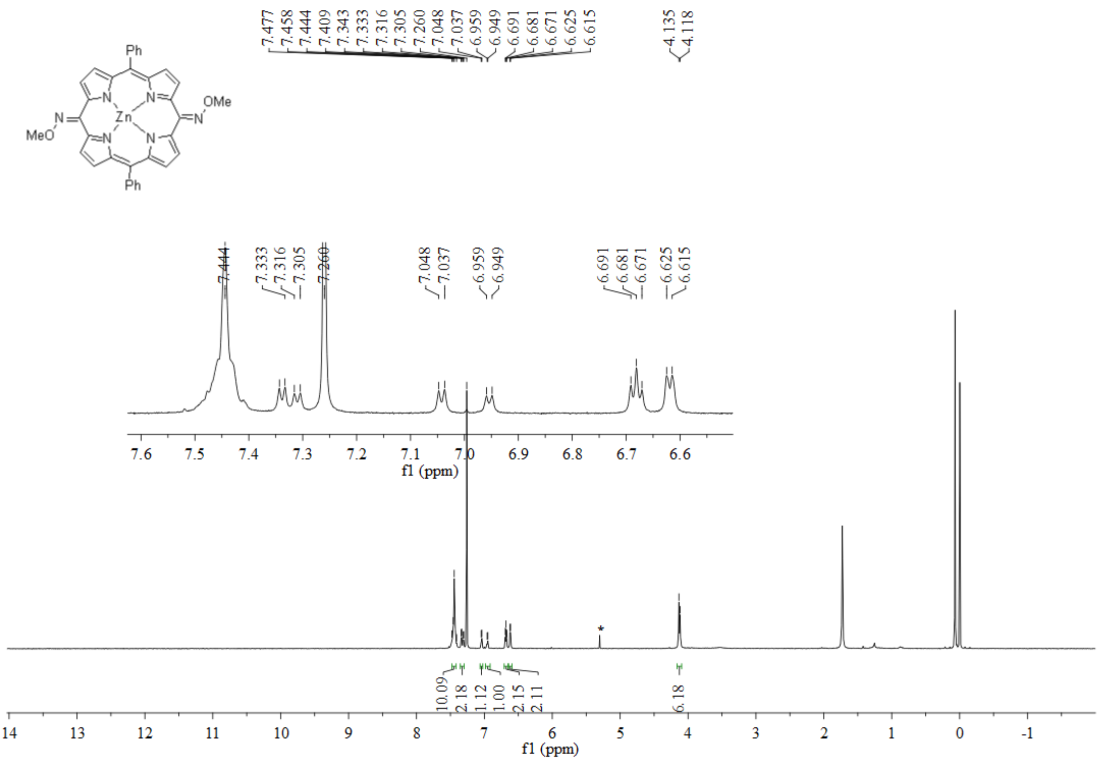

^{1}H NMR spectra of **Zn1b** (DMSO-d_6)

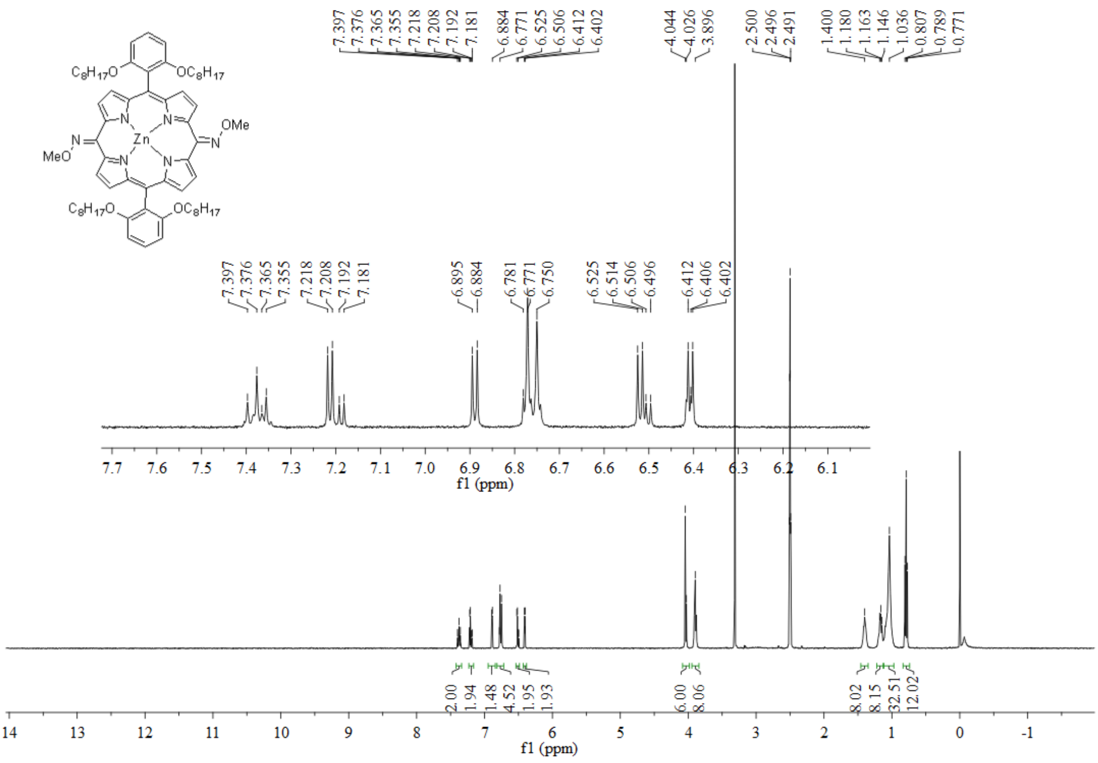

^{1}H NMR spectra of **Zn1c** ($CDCl_3$)

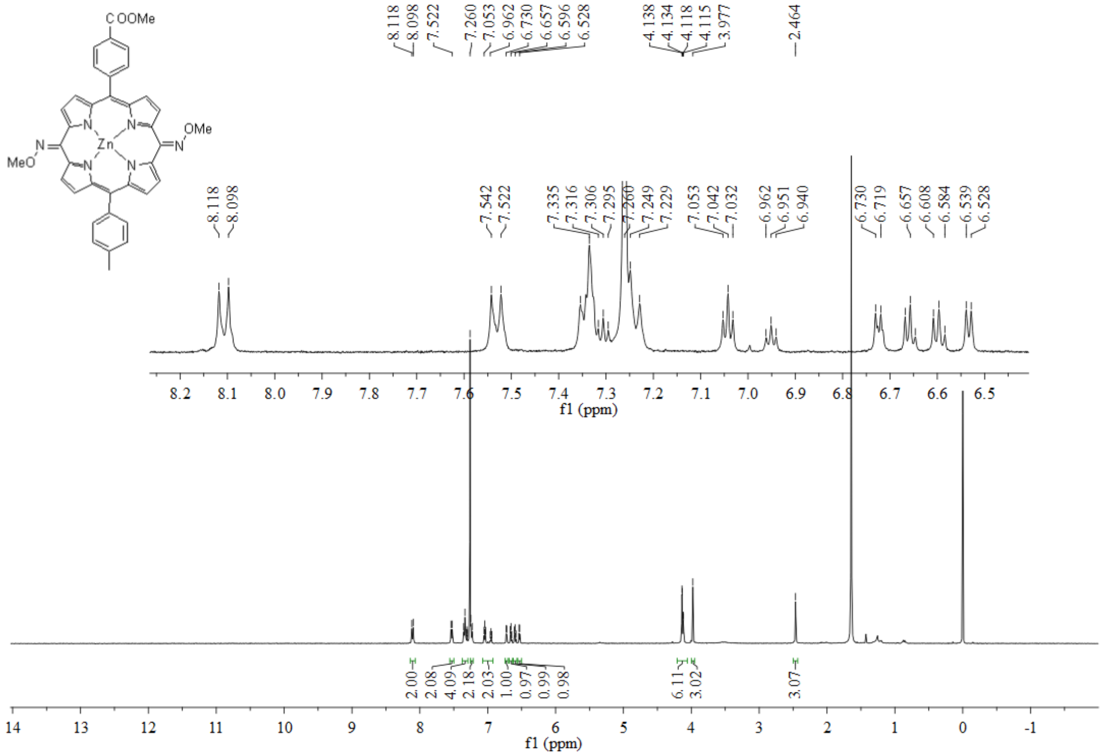

^{1}H NMR spectra of **Zn1d** (DMSO-d_6)

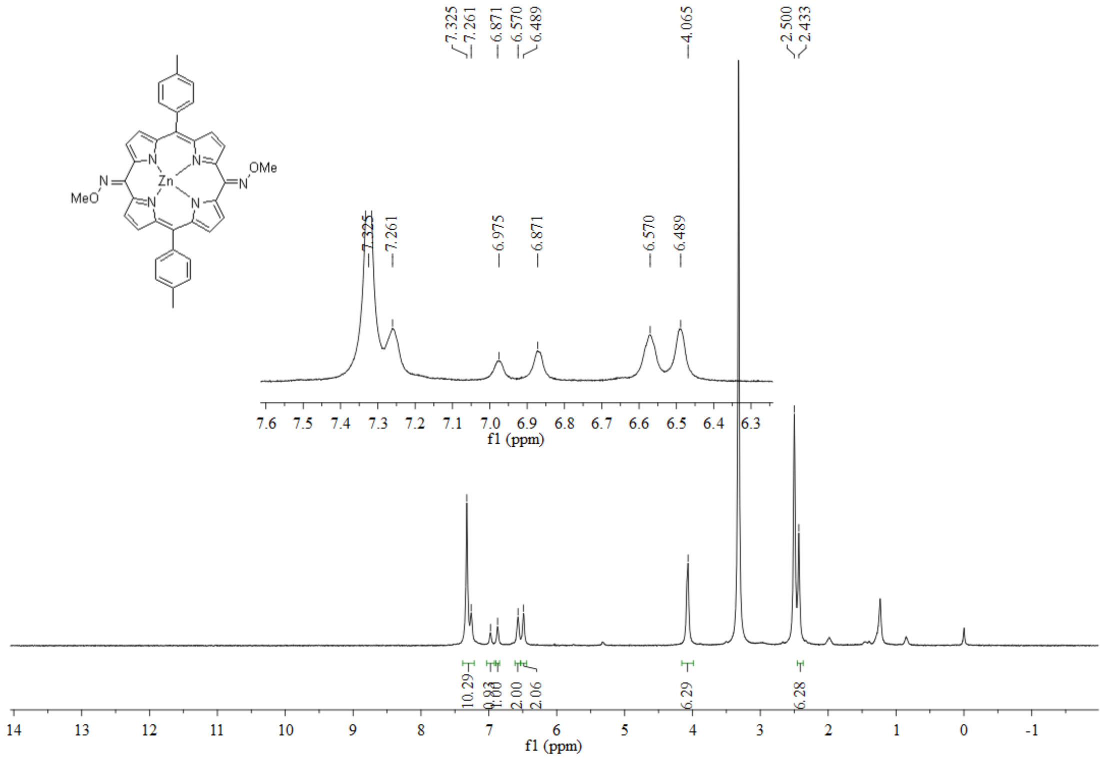

^{1}H NMR spectra of **Zn2b** ($CDCl_3$)

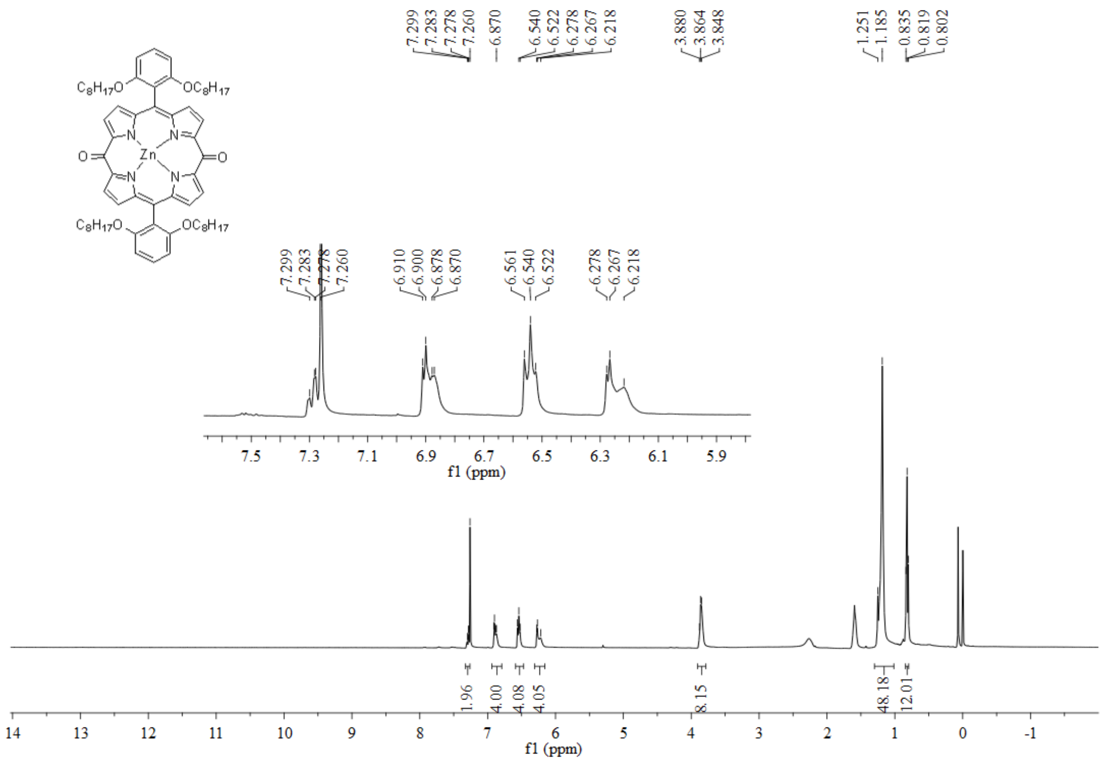

^{1}H NMR spectra of **3aa** (DMSO-d_6), * represent solvent peak.

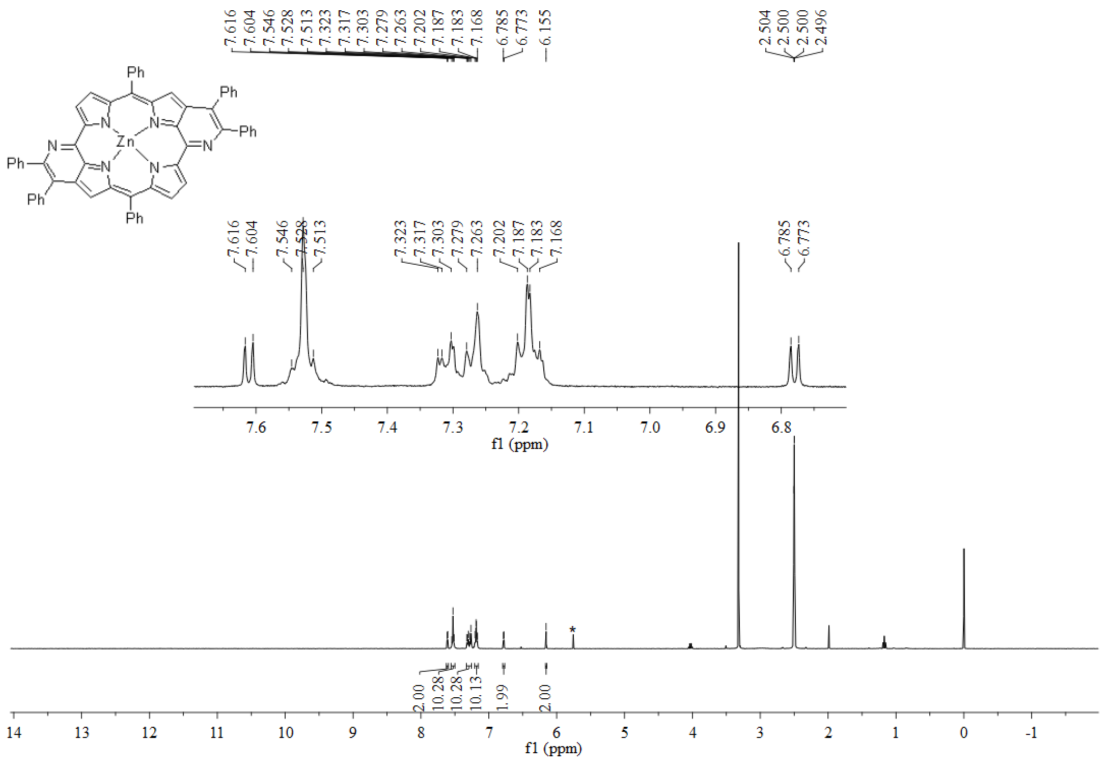

^{1}H NMR spectra of **3ab** (DMSO-d_6)

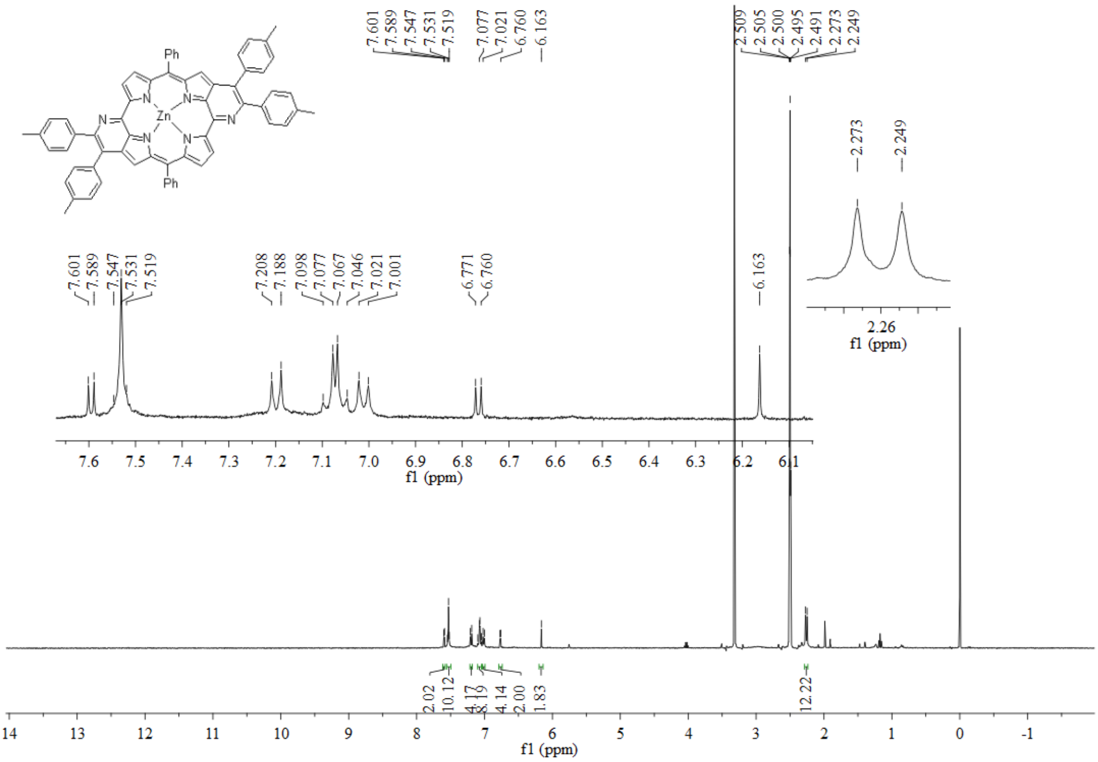

^{1}H NMR spectra of **3ac** (DMSO-d_6)

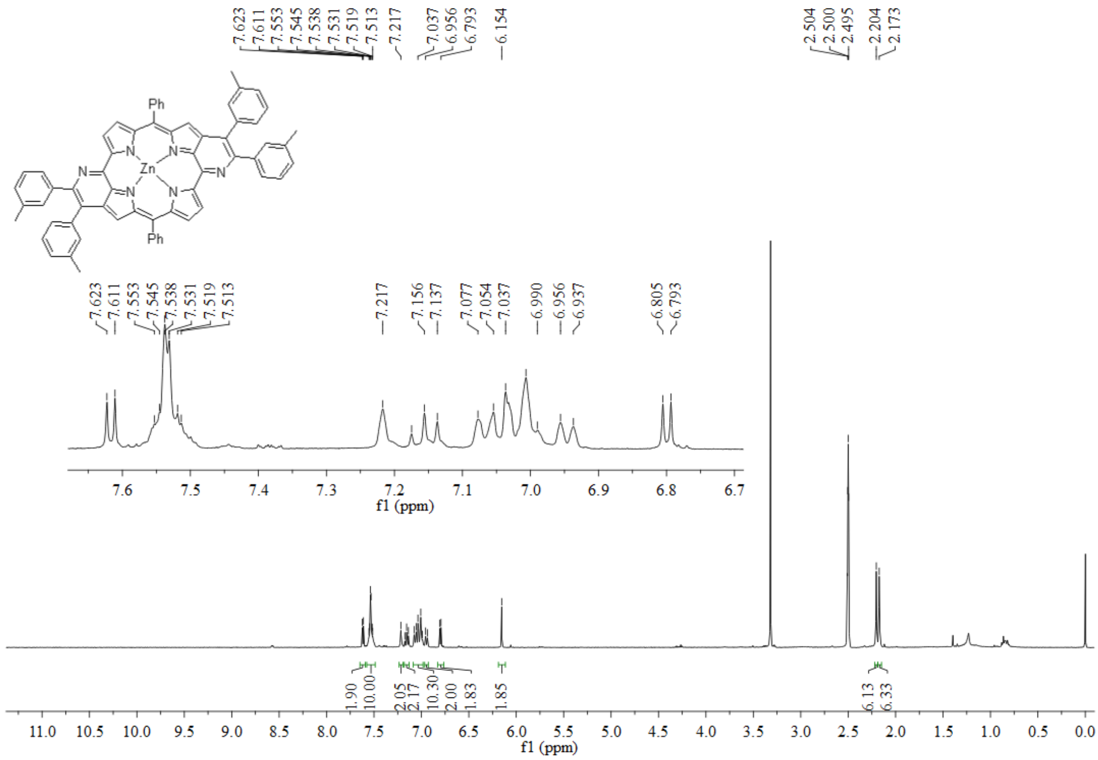

^{1}H NMR spectra of **3ad** (DMSO-d_6), * represent solvent peak.

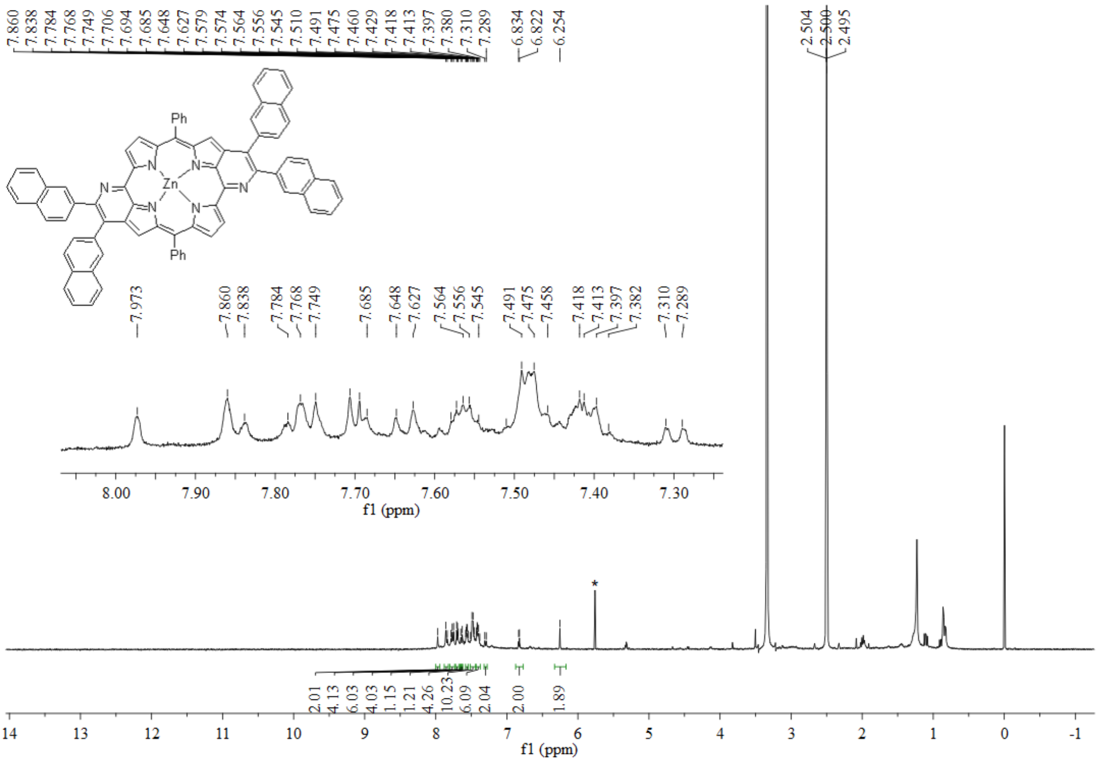

^{1}H NMR spectra of **3ae** ($CDCl_3$)

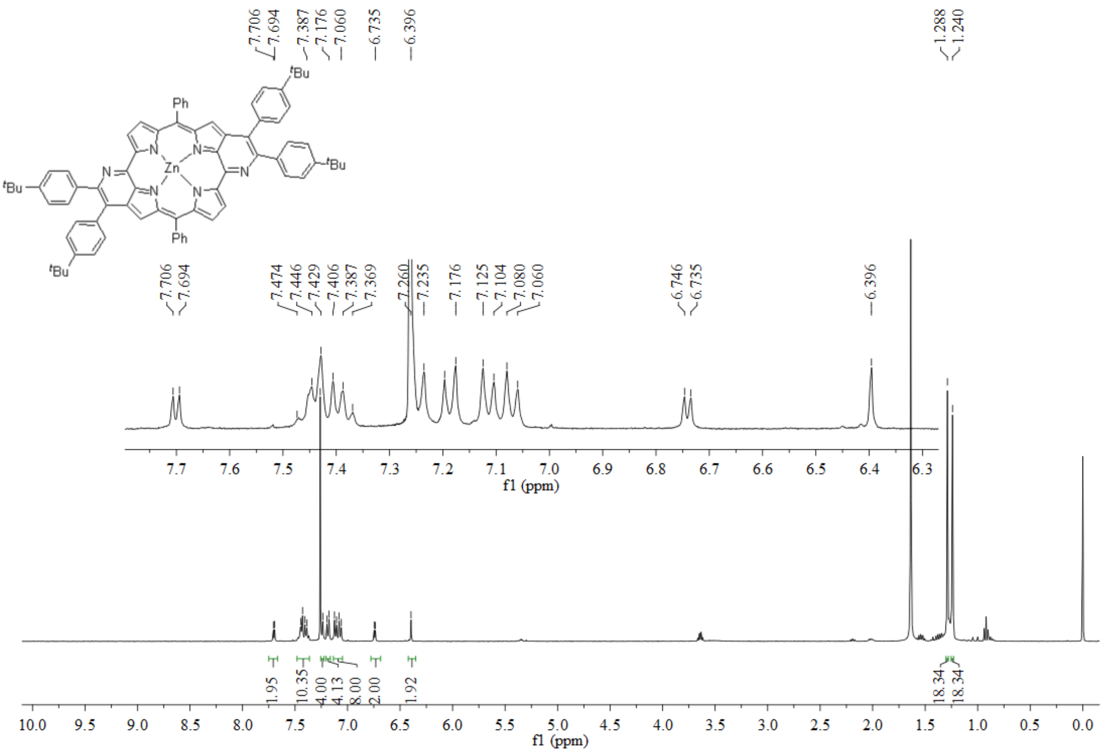

^{1}H NMR spectra of **3af** ($CDCl_3$)

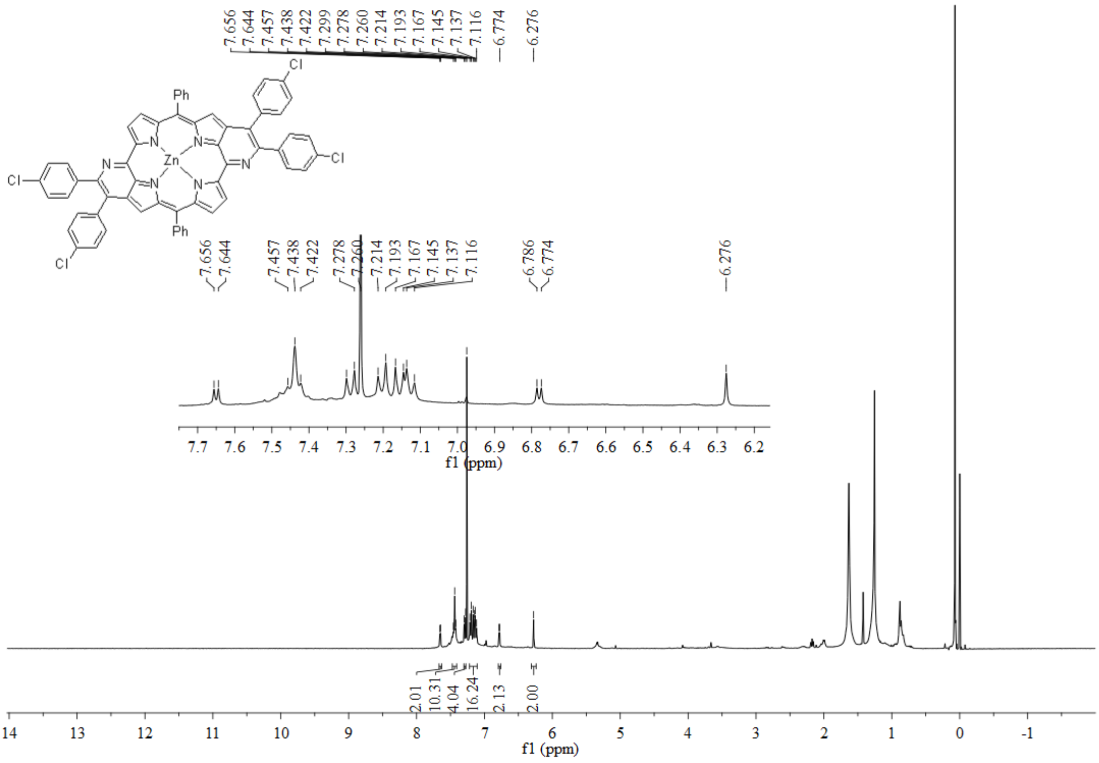

^{1}H NMR spectra of **3ah** (DMSO-d_6)

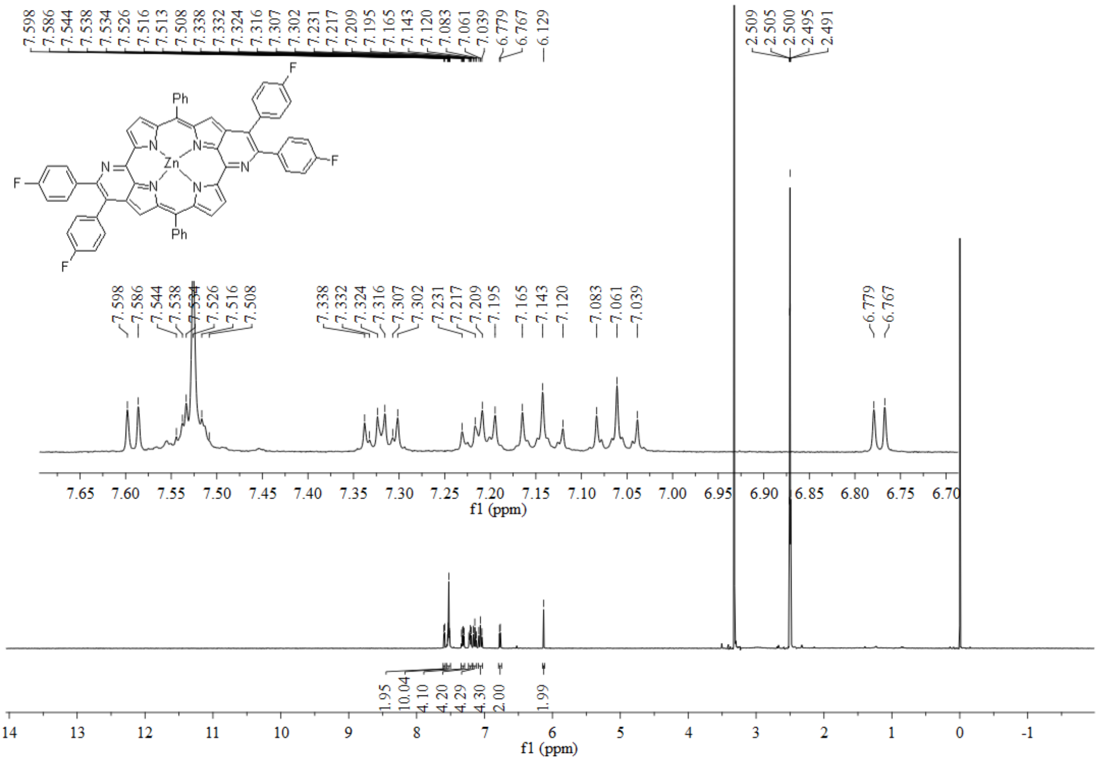

^{1}H NMR spectra of **3ai** (DMSO-d_6)

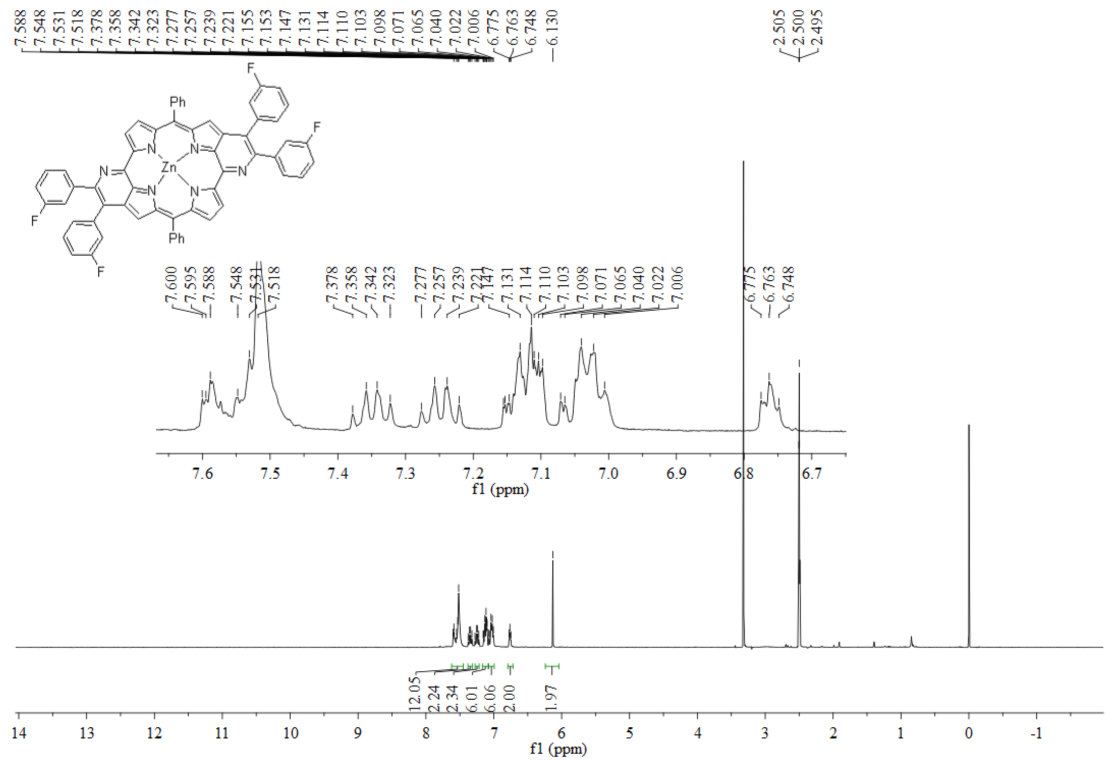

^{1}H NMR spectra of **3aj** (DMSO-d_6)

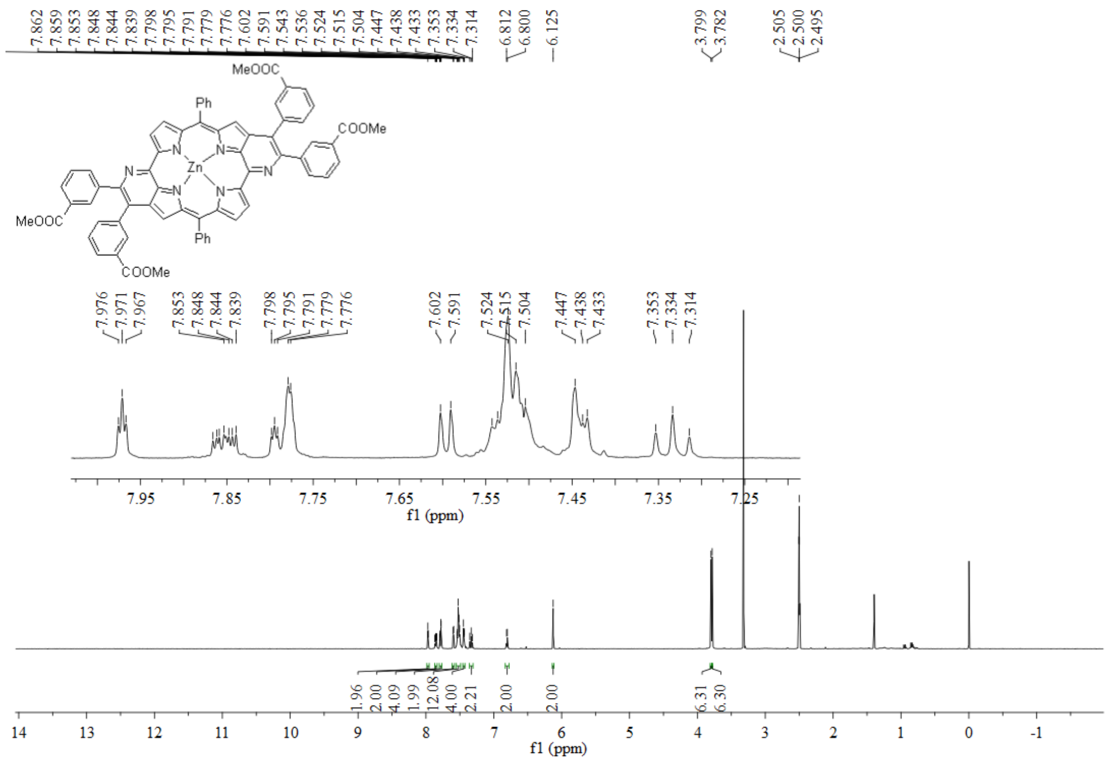

^{1}H NMR spectra of **3ak** (DMSO-d_6)

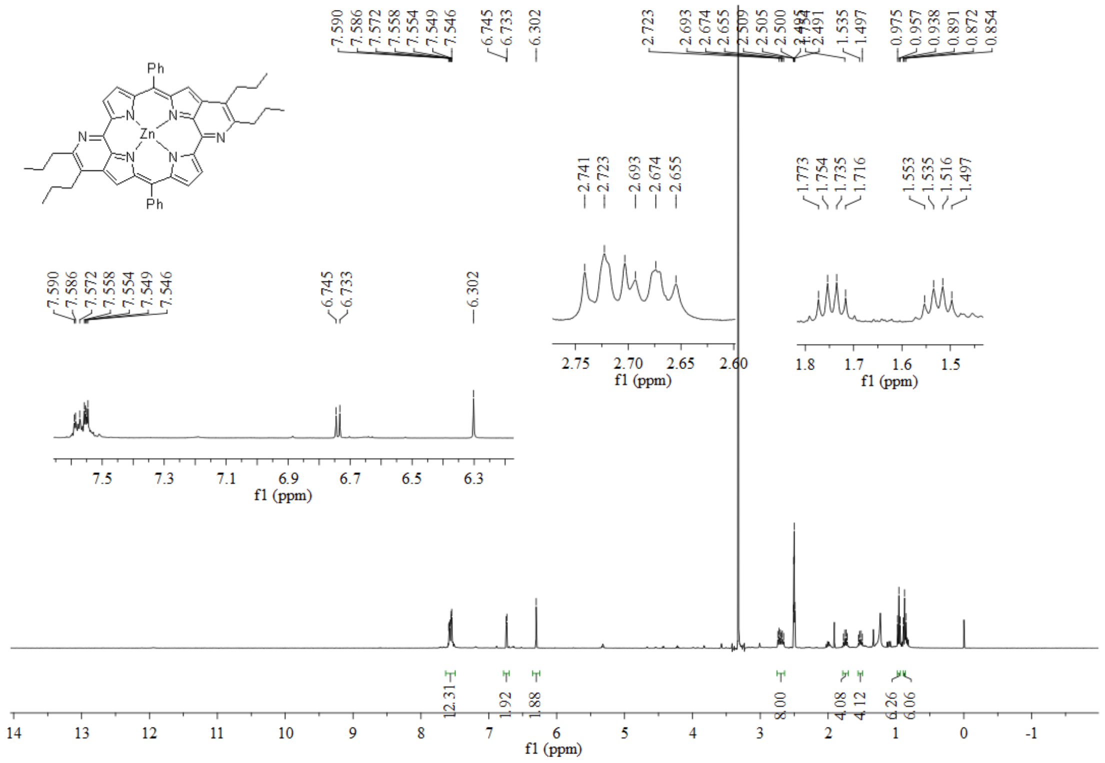

^{1}H NMR spectra of **3ba** ($CDCl_3$), * represent solvent peak.

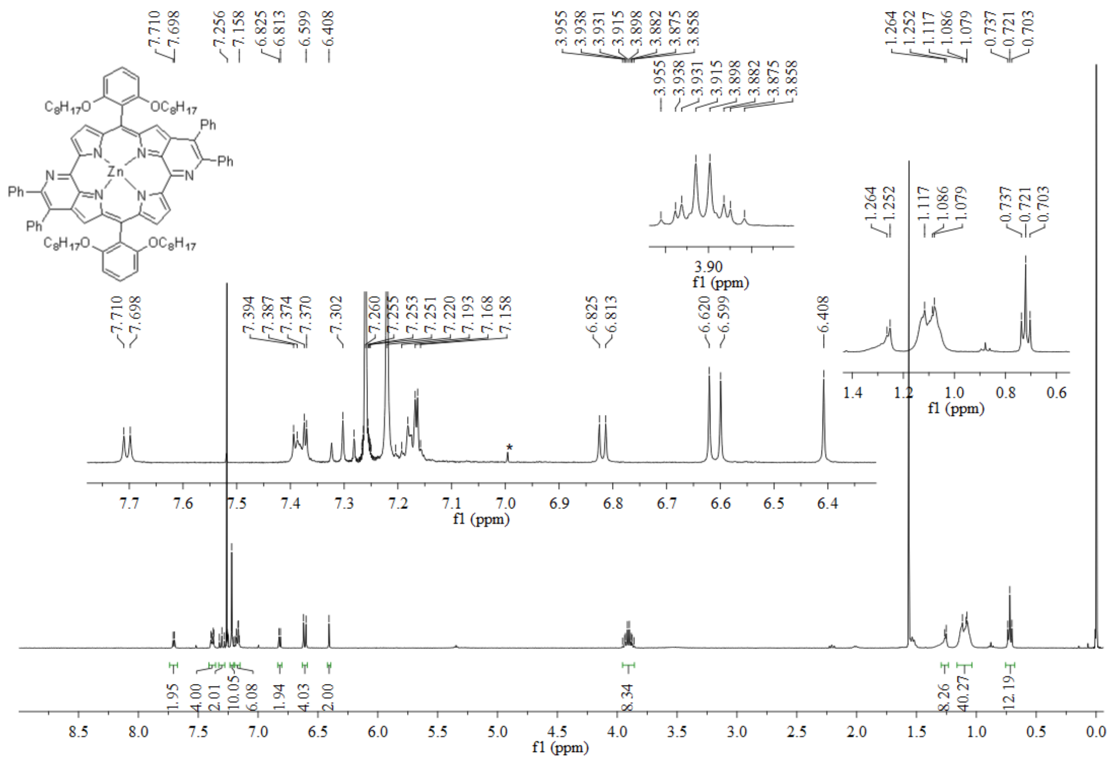

^{1}H NMR spectra of **3ca** ($CDCl_3$)

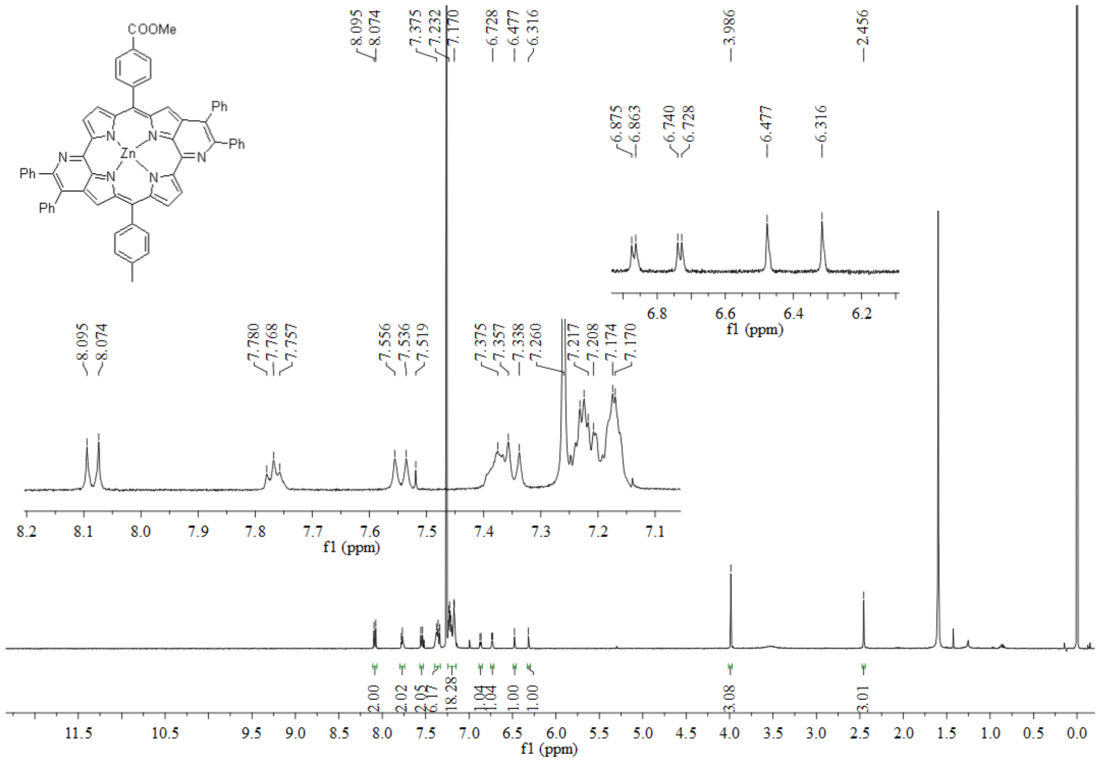

^{1}H NMR spectra of **4aa** (DMSO-d_6)

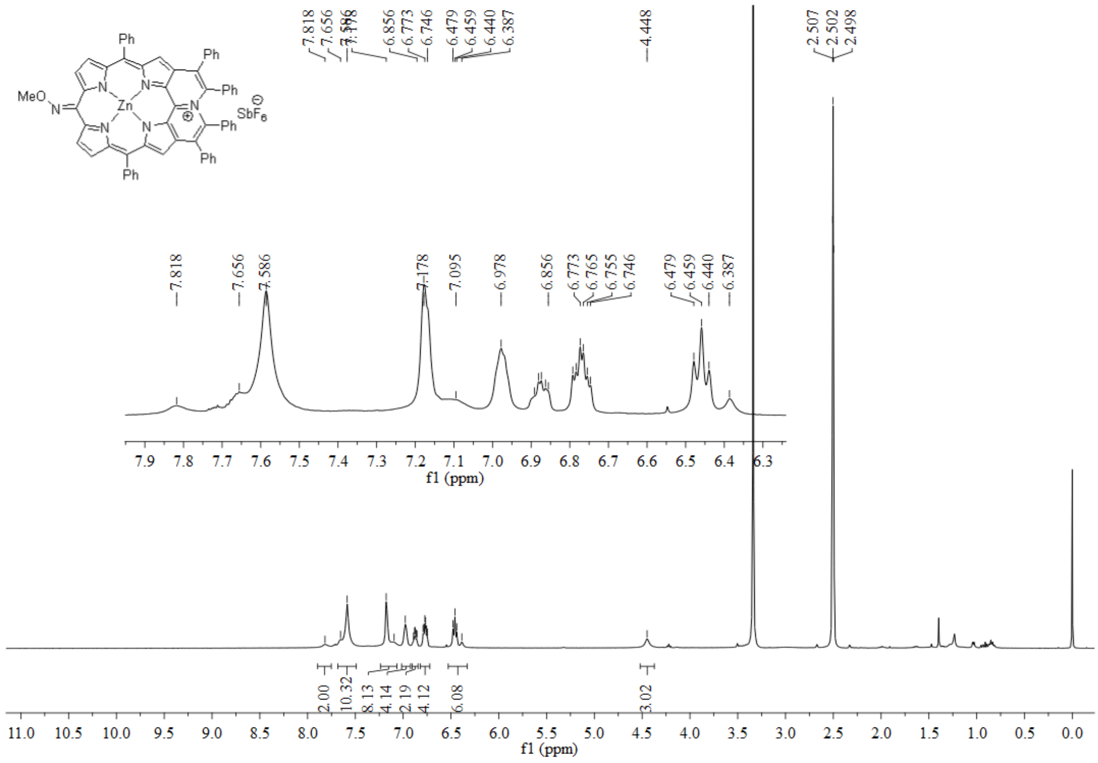

^{1}H NMR spectra of **4ac** (DMSO-d_6), * represent solvent peak.

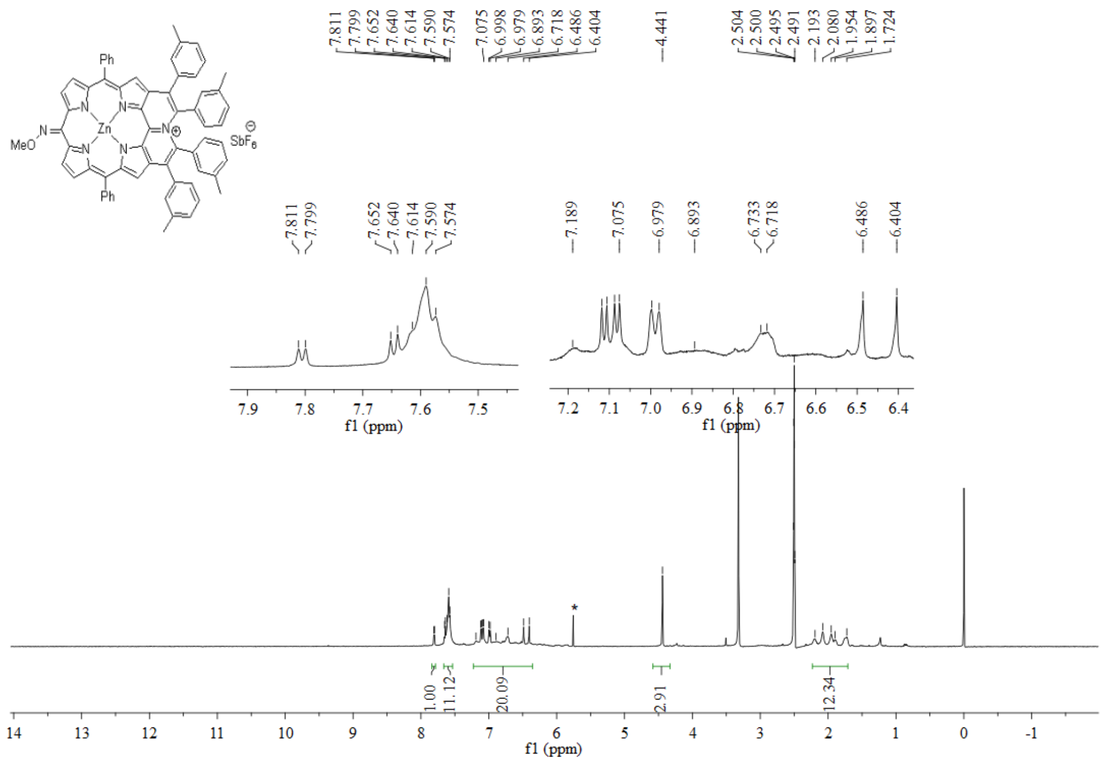

^{1}H NMR spectra of **4af** (DMSO-d_6)

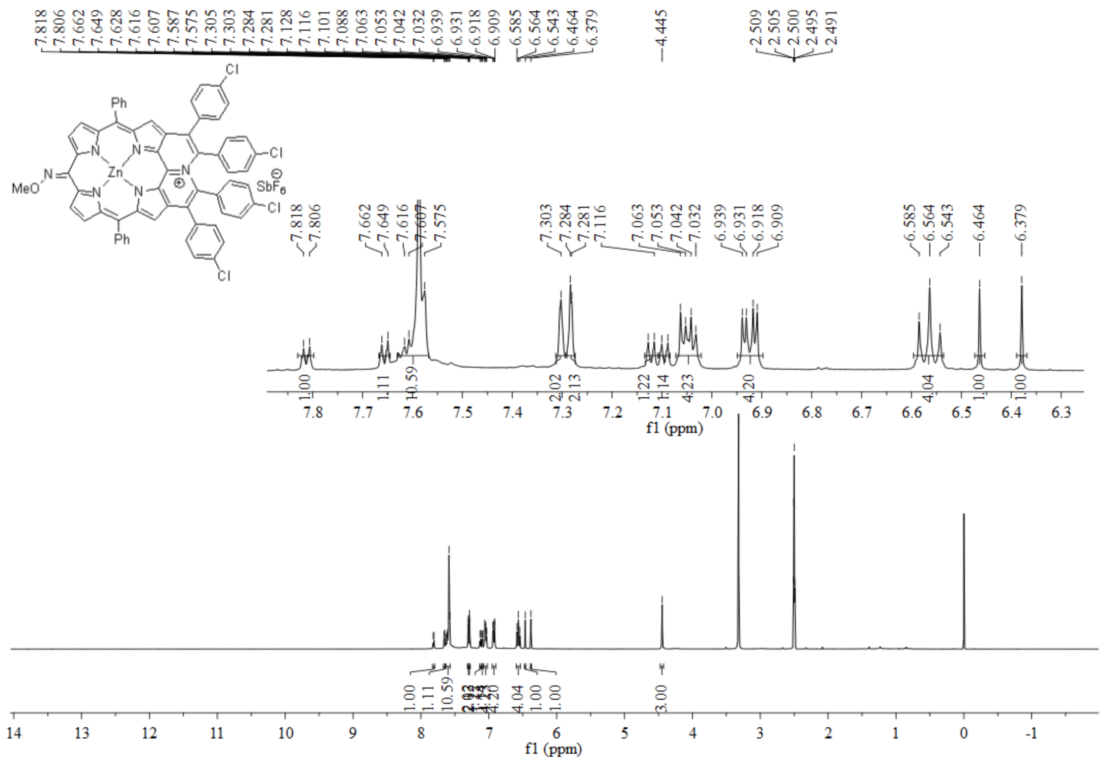

^{1}H NMR spectra of **4ah** (DMSO-d_6)

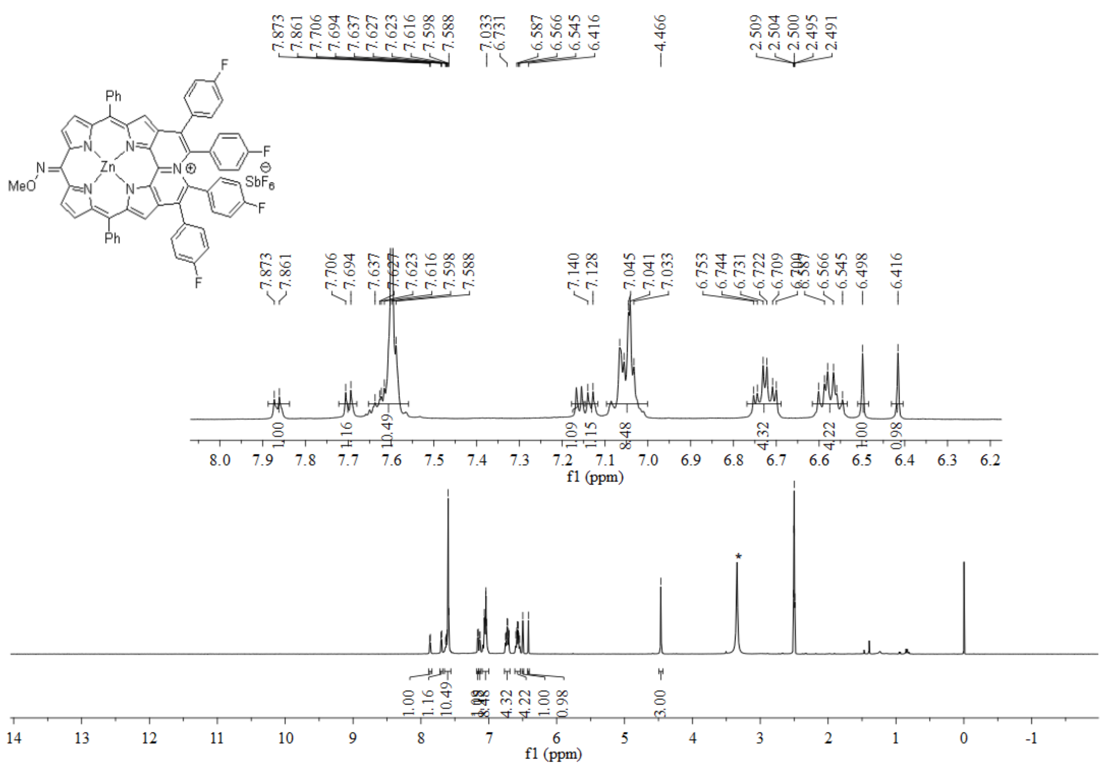

^{1}H NMR spectra of **4ai** (DMSO-d_6)

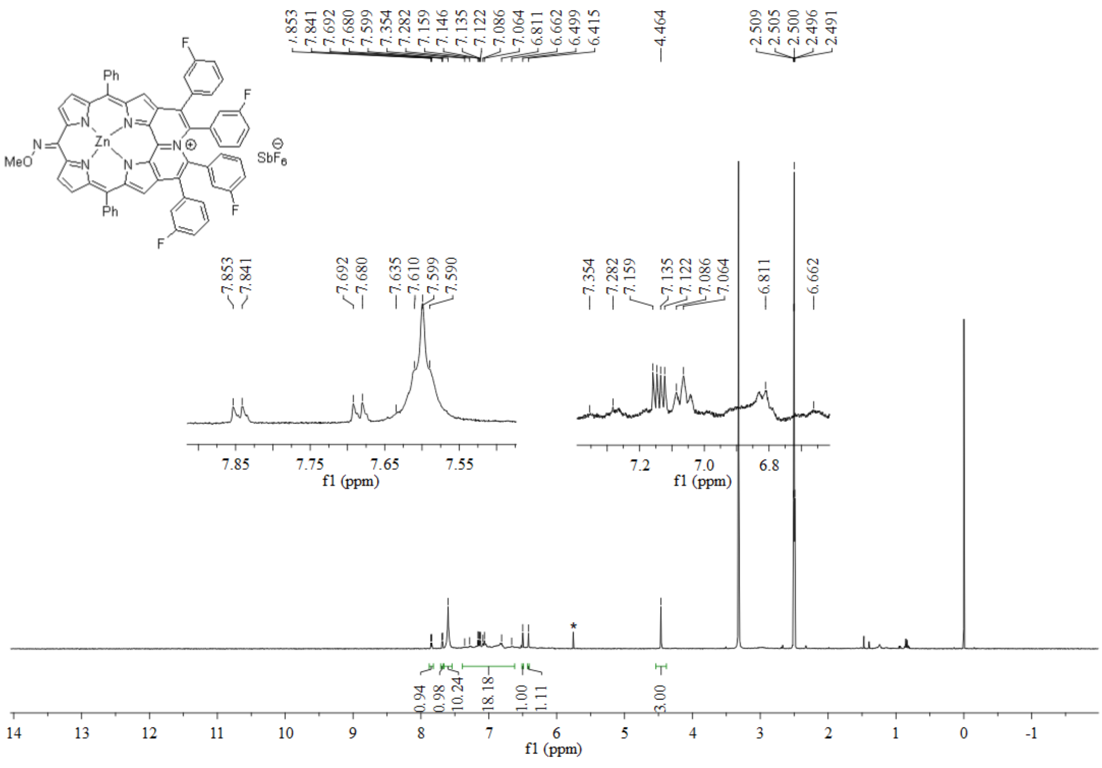

^{1}H NMR spectra of **4al** (DMSO-d_6)

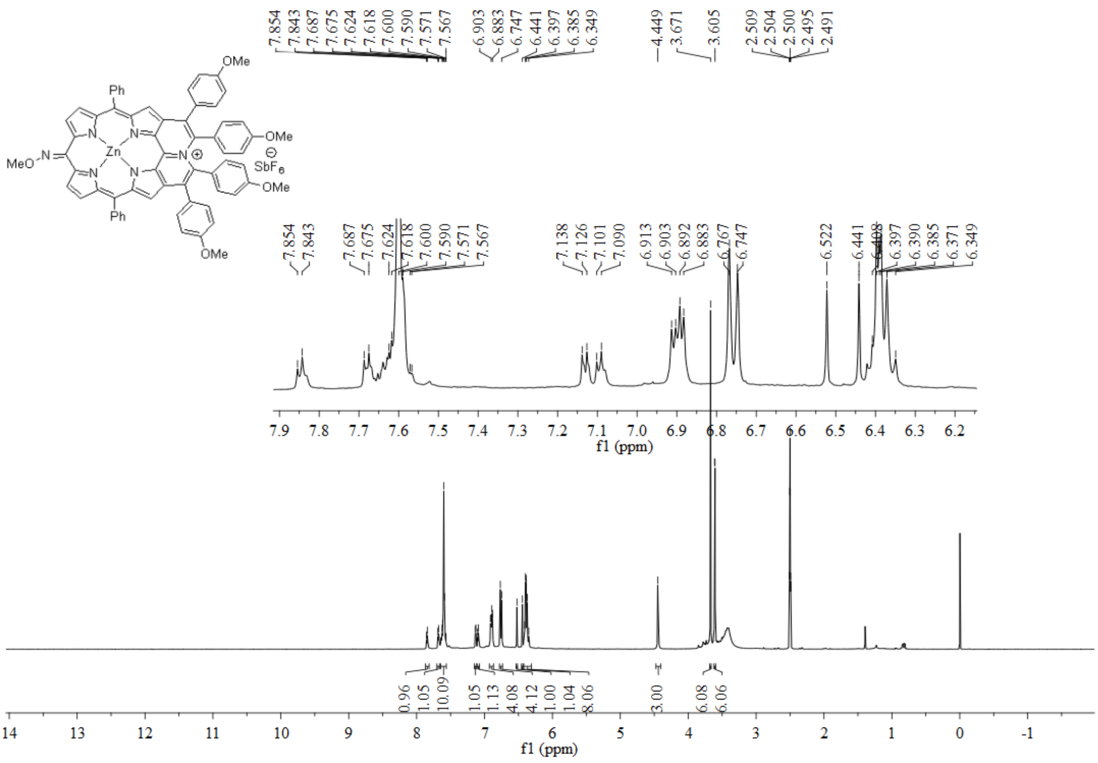

^{1}H NMR spectra of **4am** (DMSO-d_6)

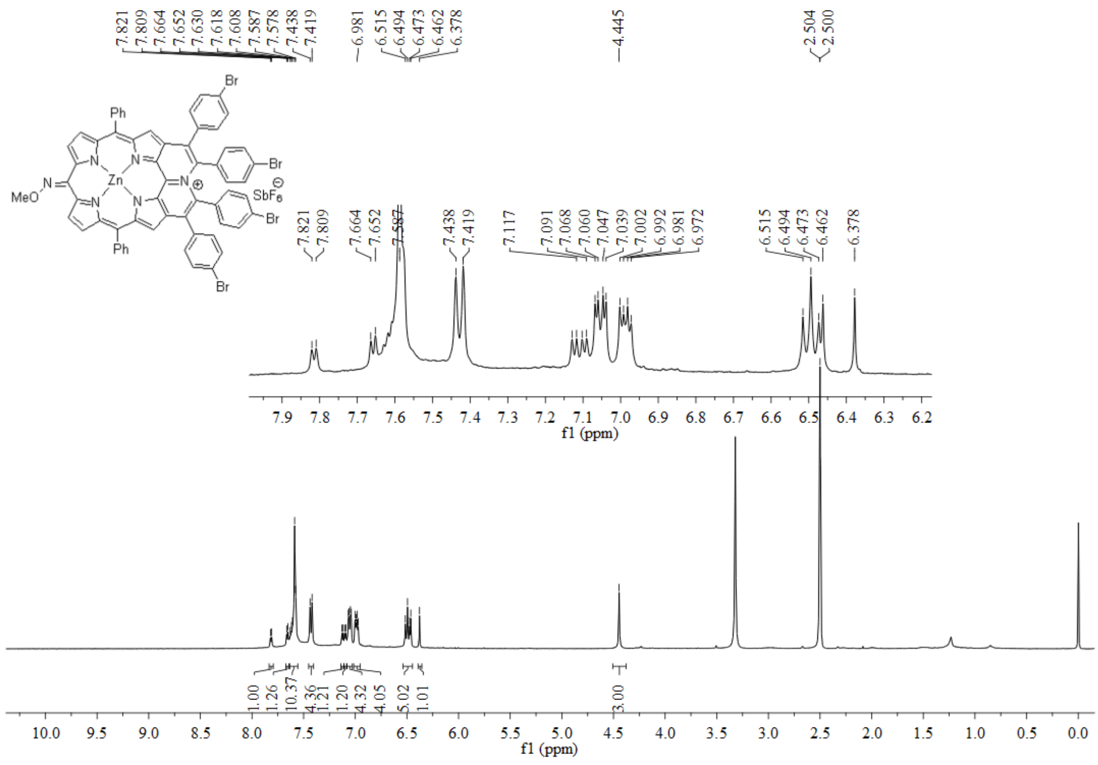

^{1}H NMR spectra of **4an** (DMSO-d_6)

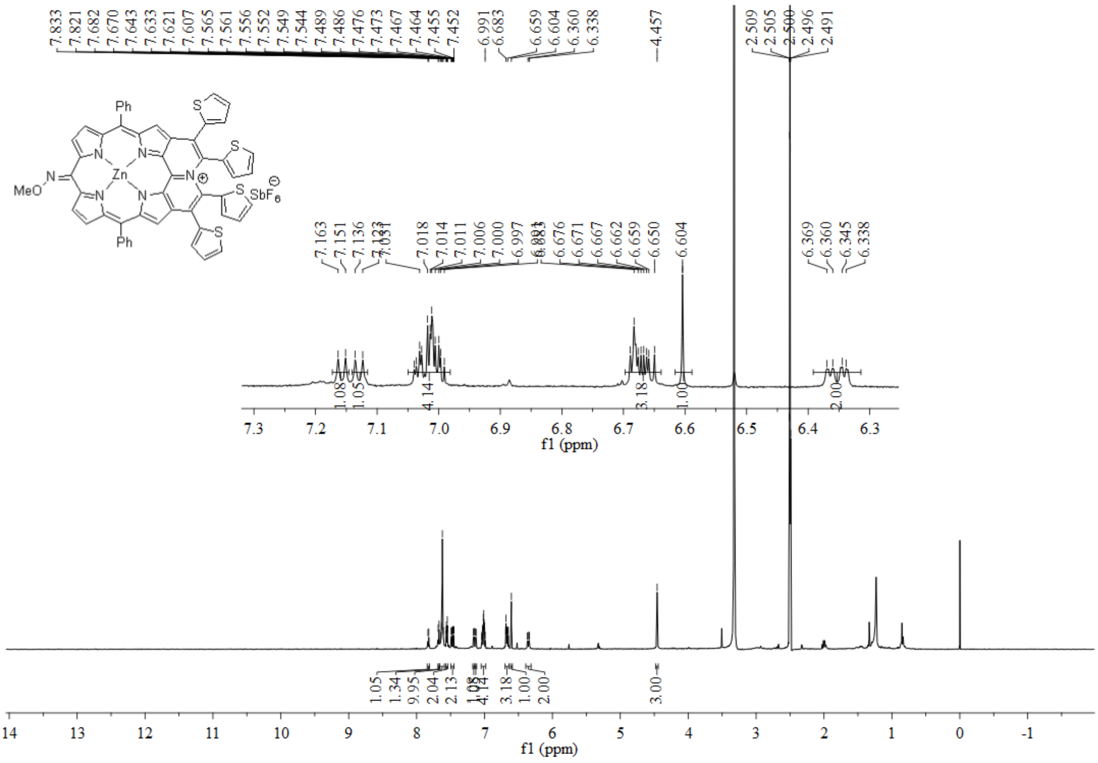

^{1}H NMR spectra of **4da** (DMSO-d_6), * represent solvent peak.

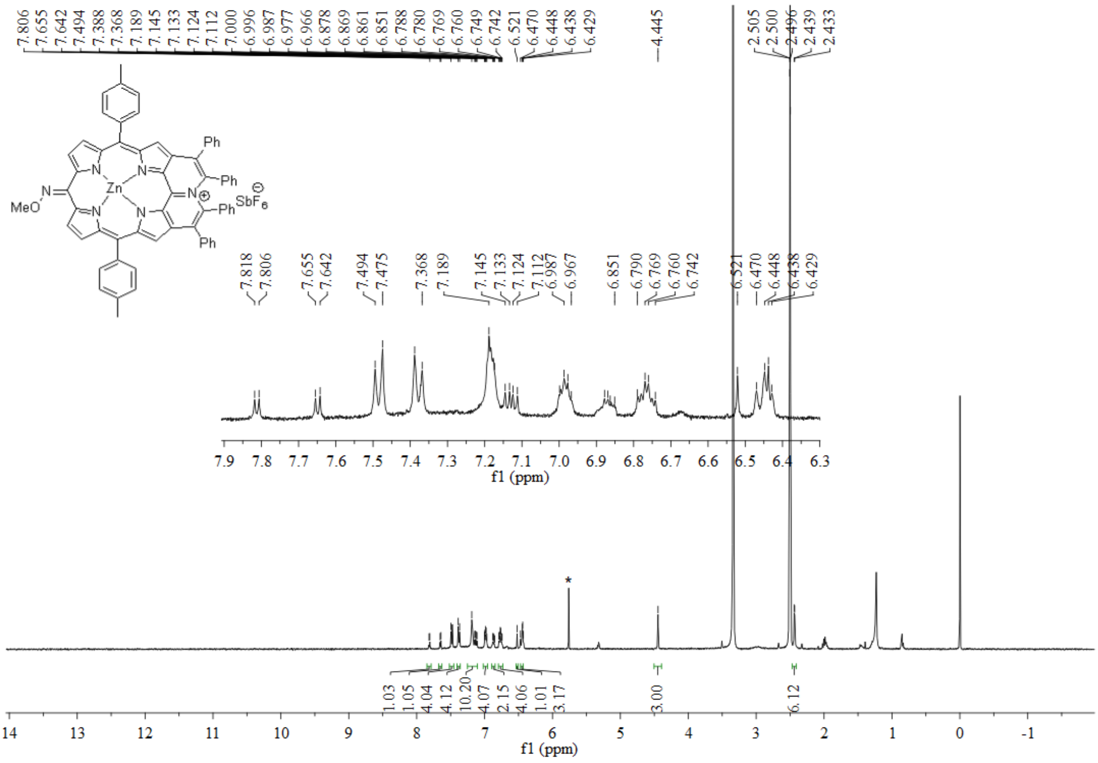

^{1}H NMR spectra of the mixture of **5aa** ($CDCl_3$)

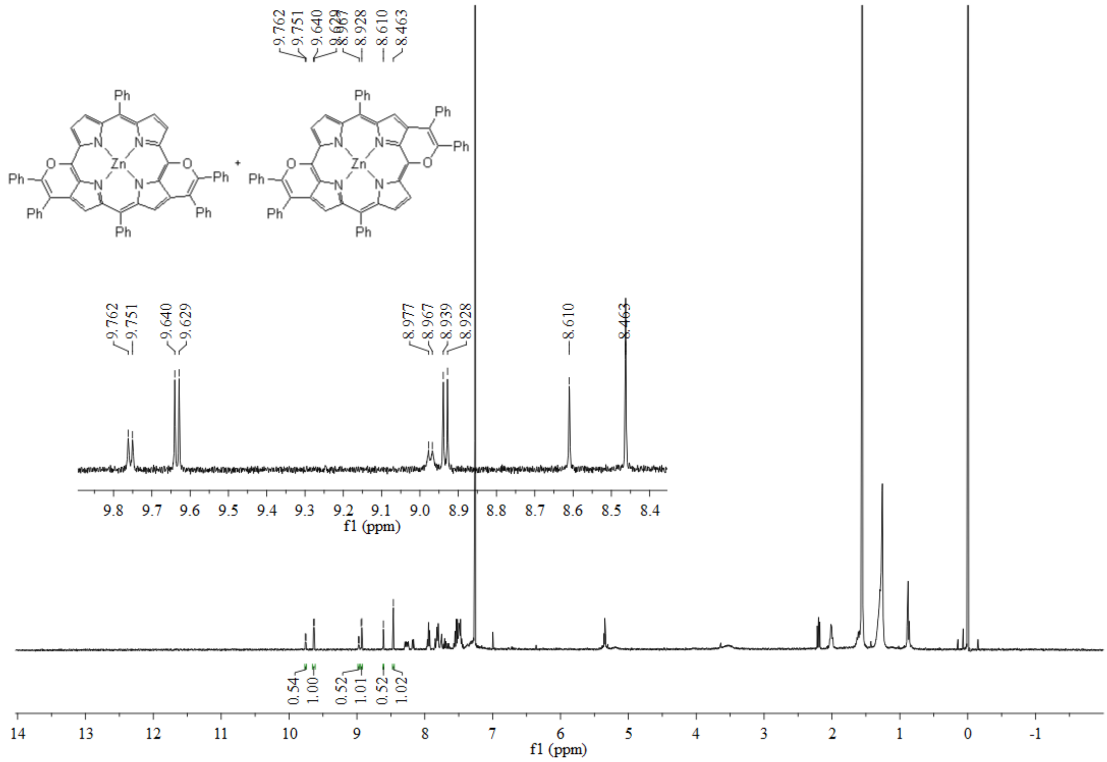

^{1}H NMR spectra of **5ab** ($CDCl_3$)

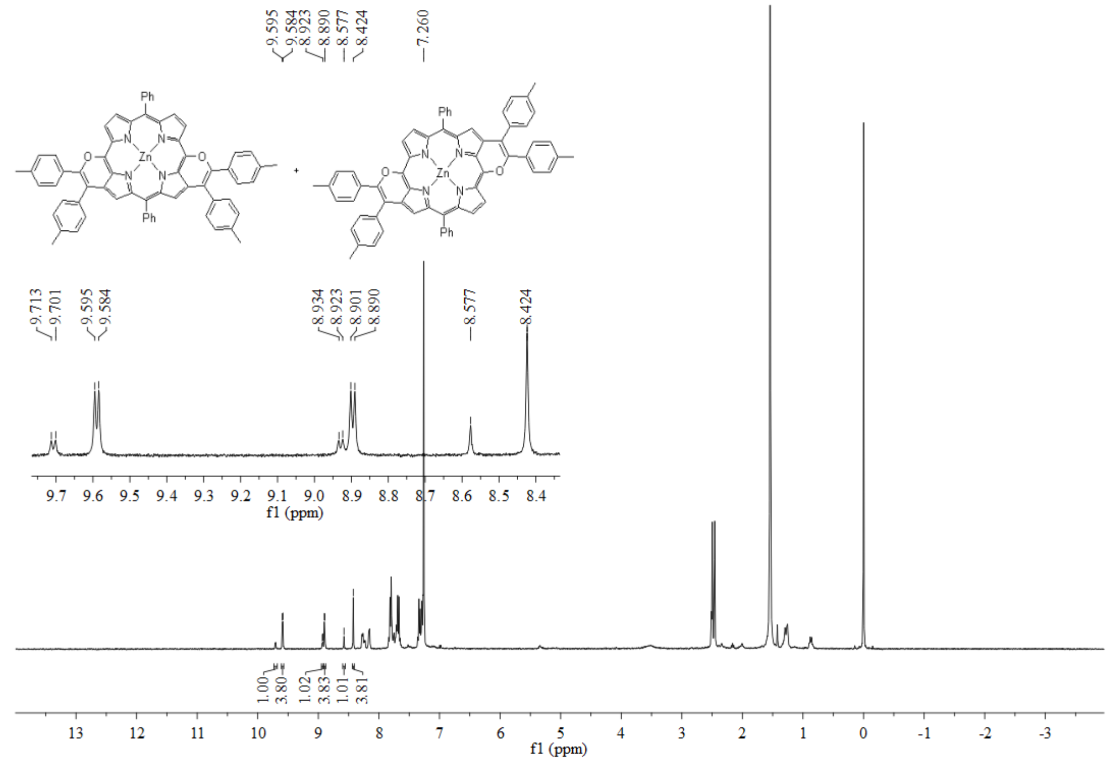

^{1}H NMR spectra of **5ao** ($CDCl_3$)

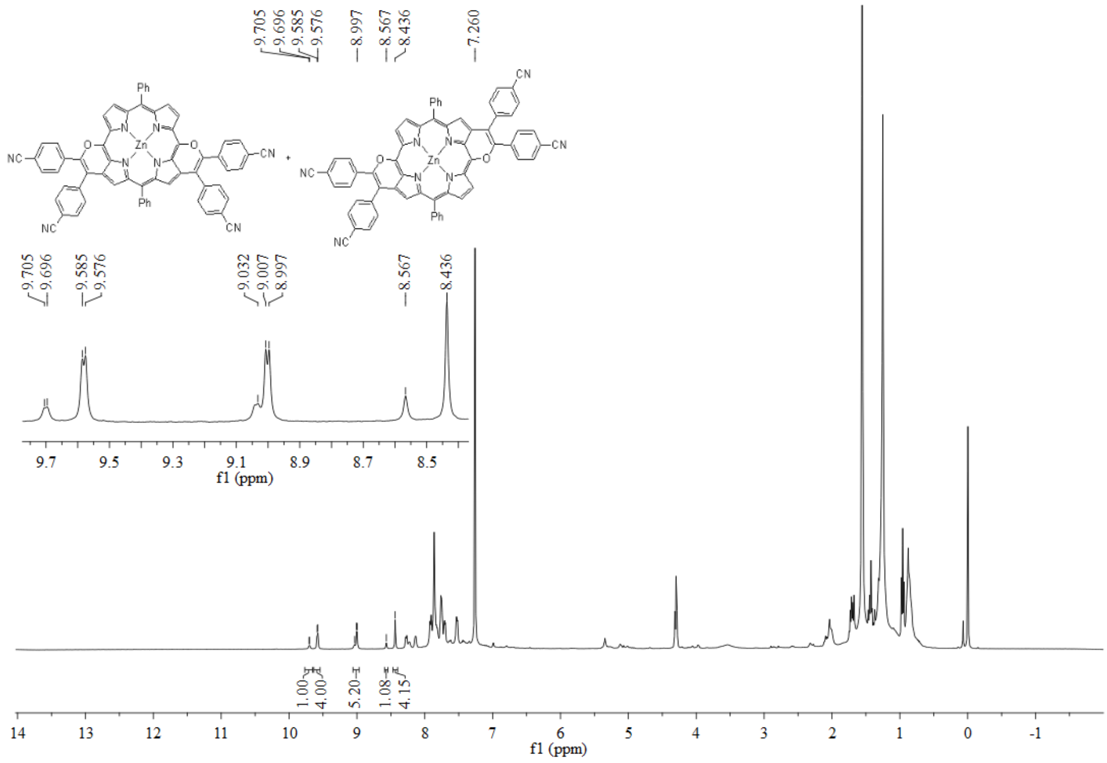

^{1}H NMR spectra of the mixture of **5ah** ($CDCl_3$)

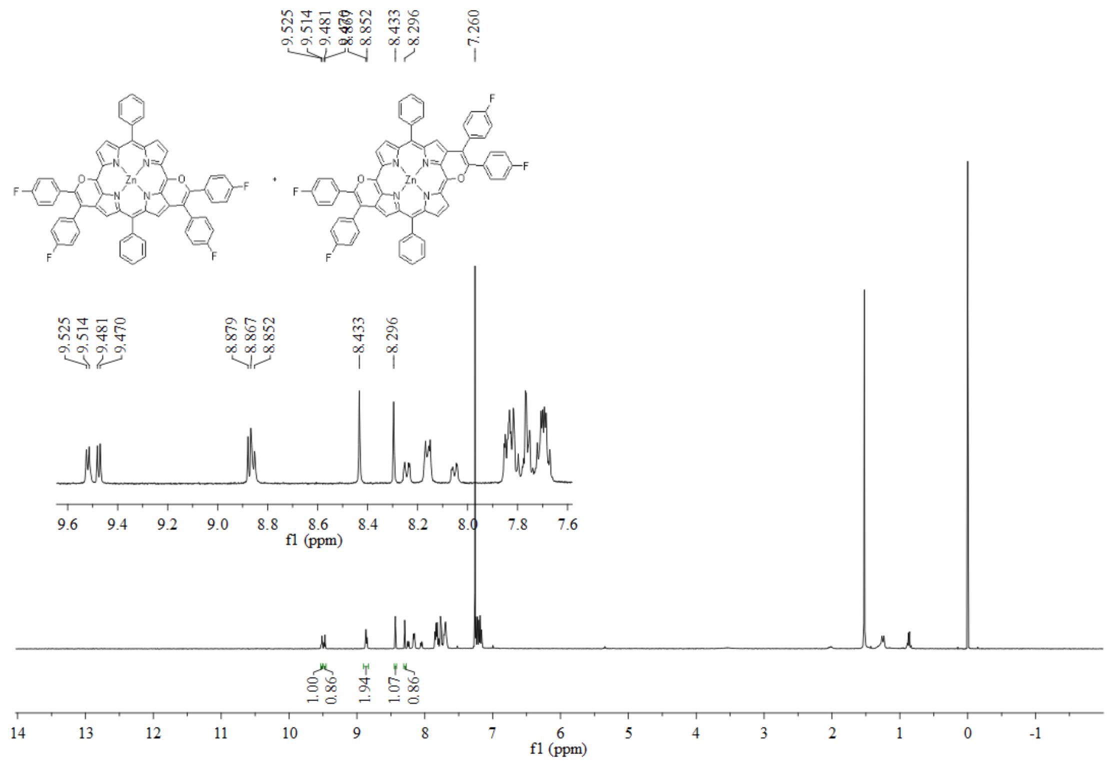

^{1}H NMR spectra of *syn*-**5ah** ($CDCl_3$)

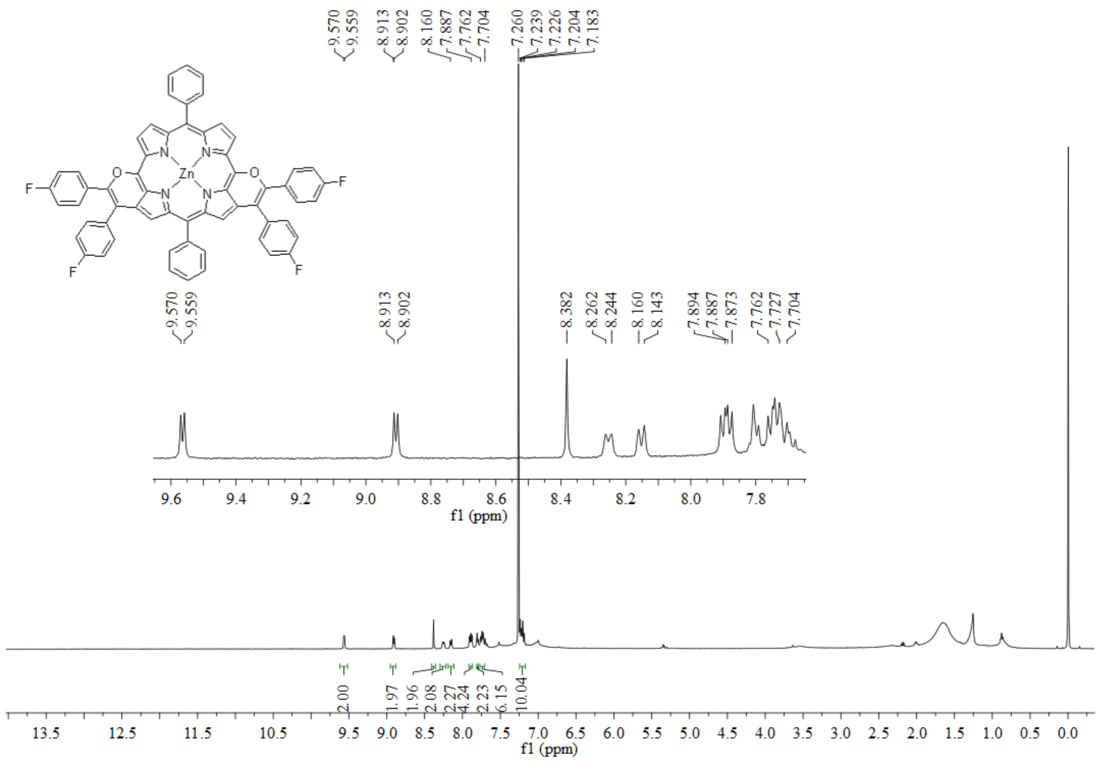

^{1}H NMR spectra of *anti*-**5ah** ($CDCl_3$)

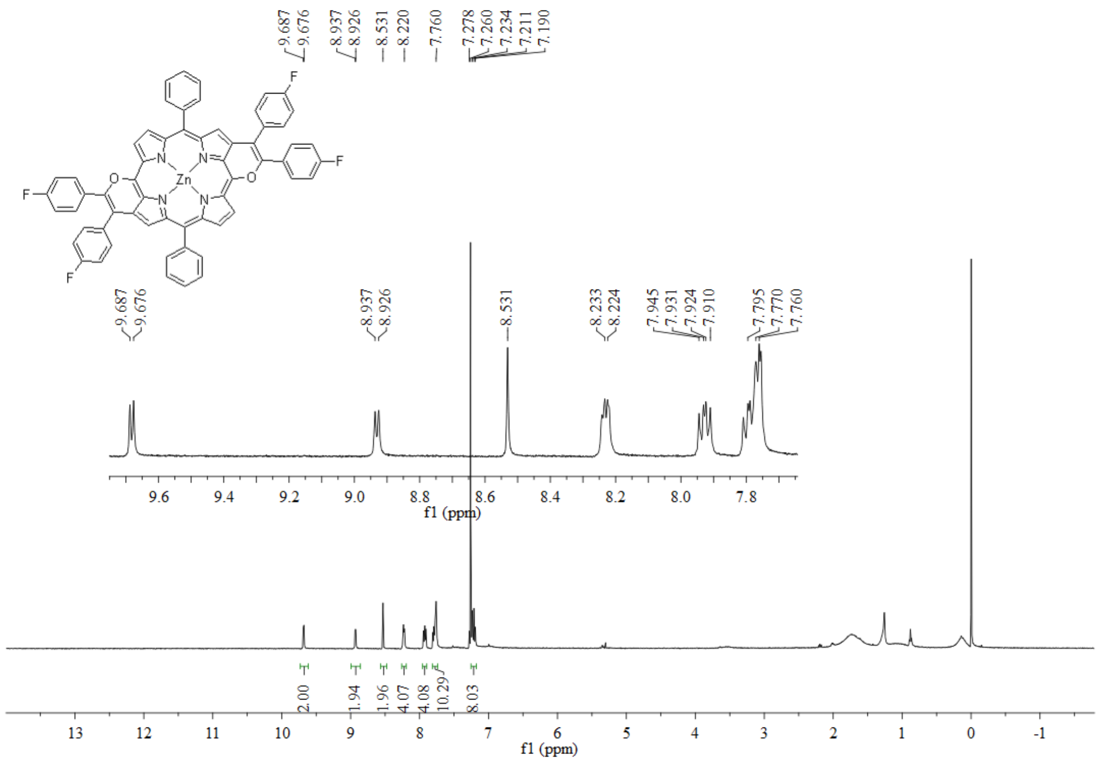

^{1}H NMR spectra of *syn*-**5af** ($CDCl_3$), * represent solvent peak.

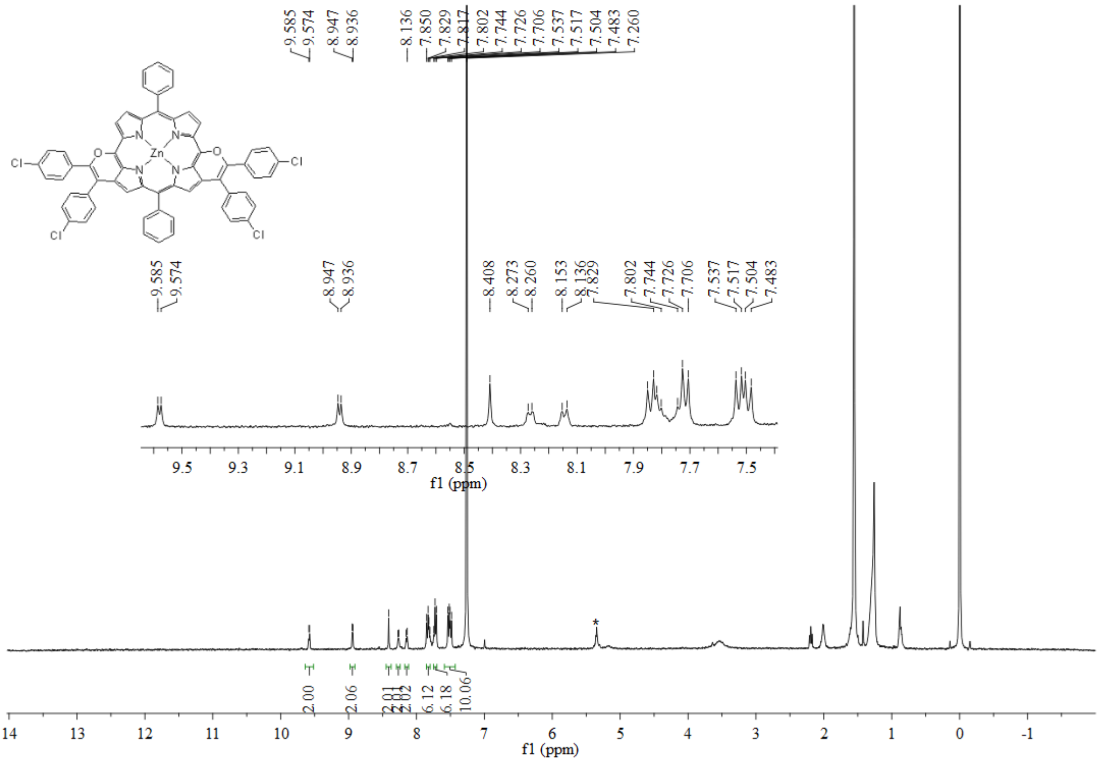

^{1}H NMR spectra of *syn*-**5al** ($CDCl_3$)

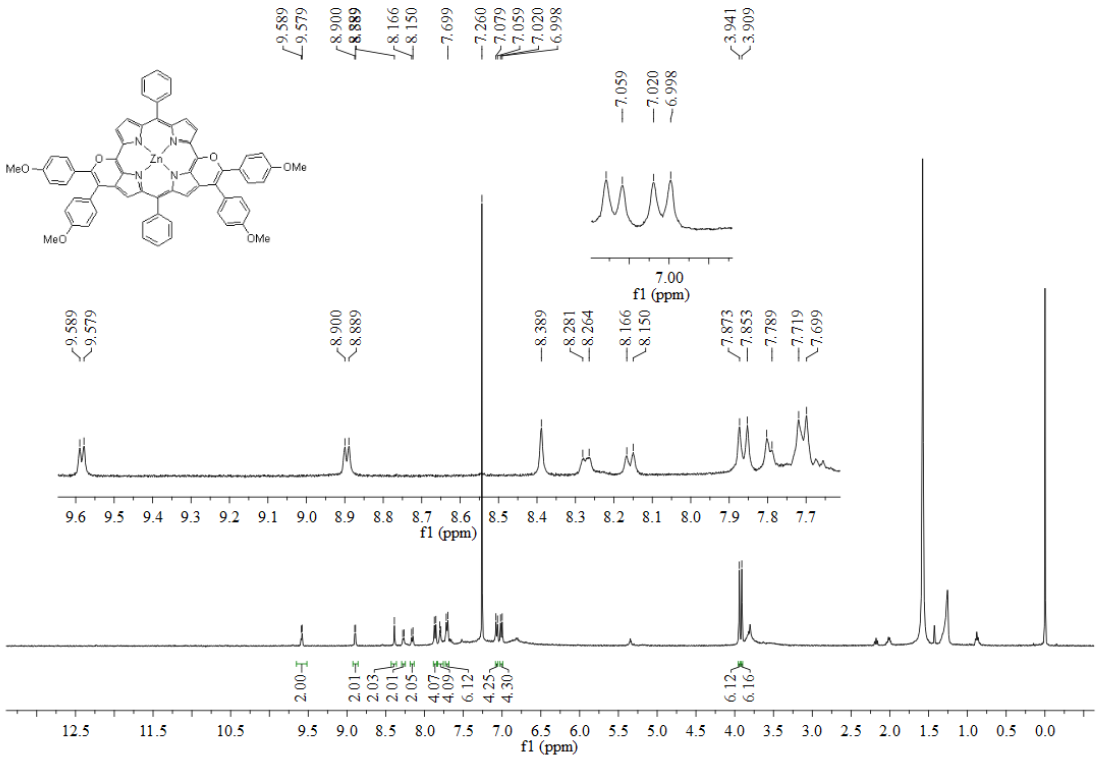

^{1}H NMR spectra of **5ea** ($CDCl_3$), * represent solvent peak.

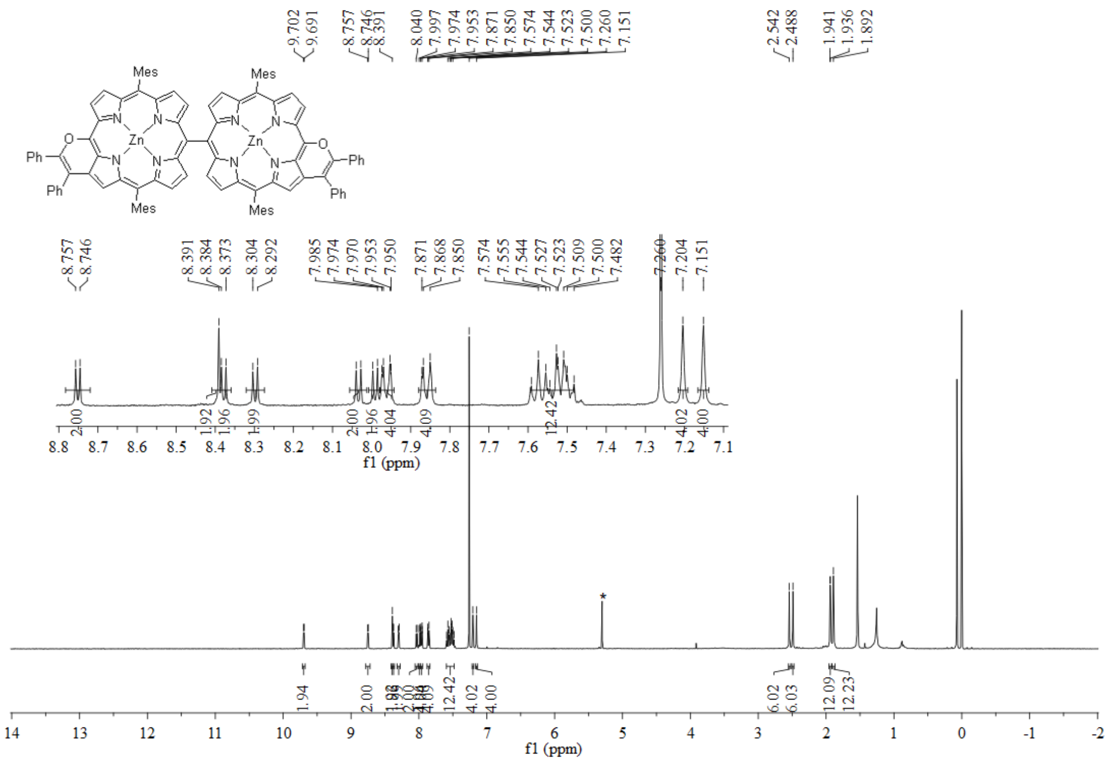

^{1}H NMR spectra of **5eh** ($CDCl_3$)

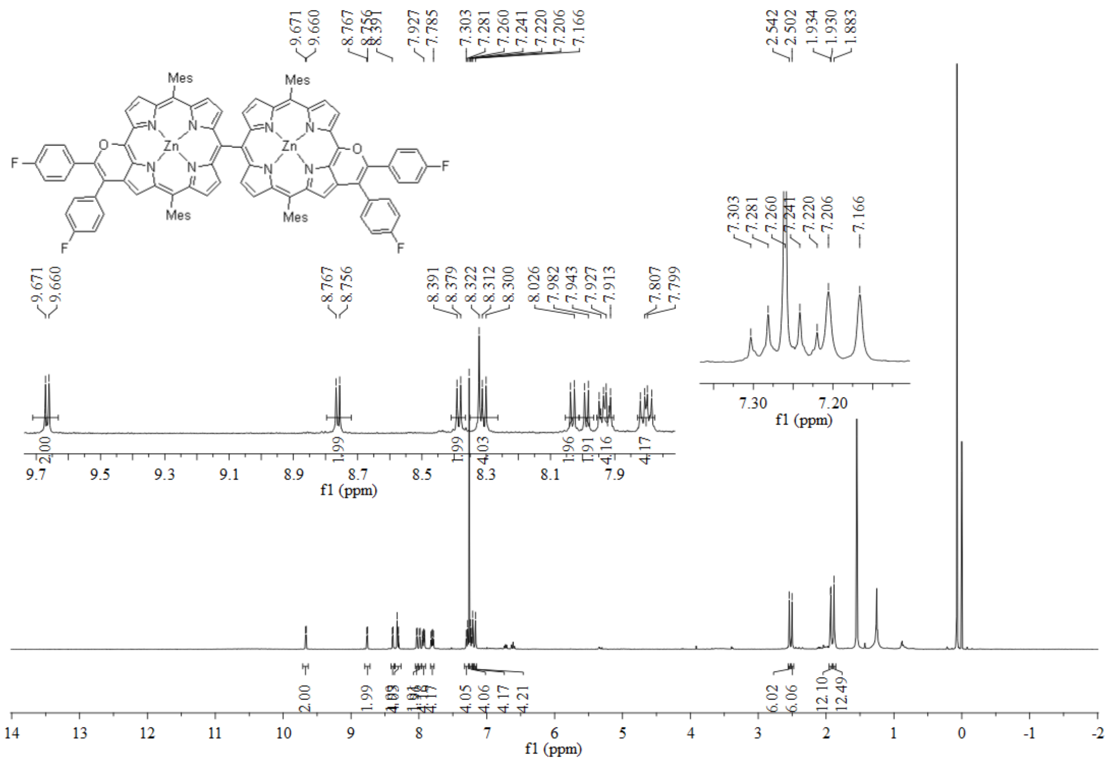

^{1}H NMR spectra of **6** (CD_2Cl_2)

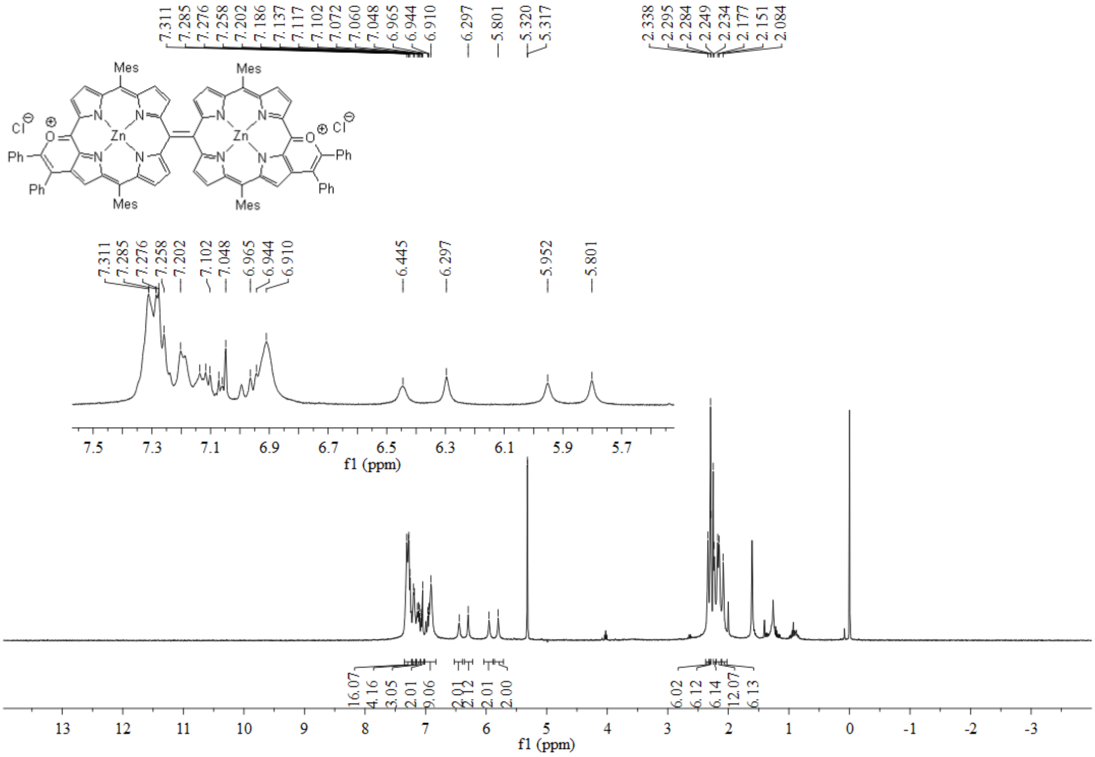

Supplement: Supplementary file 1 [file SC-010-C9SC01596E-s001.pdf]
